# Integration of copy number and transcriptomics provides risk stratification in prostate cancer: A discovery and validation cohort study

**DOI:** 10.1016/j.ebiom.2015.07.017

**Published:** 2015-07-29

**Authors:** H. Ross-Adams, A.D. Lamb, M.J. Dunning, S. Halim, J. Lindberg, C.M. Massie, L.A. Egevad, R. Russell, A. Ramos-Montoya, S.L. Vowler, N.L. Sharma, J. Kay, H. Whitaker, J. Clark, R. Hurst, V.J. Gnanapragasam, N.C. Shah, A.Y. Warren, C.S. Cooper, A.G. Lynch, R. Stark, I.G. Mills, H. Grönberg, D.E. Neal, on behalf of the CamCaP Study Group

**Affiliations:** a Cancer Research UK Cambridge Institute, University of Cambridge, Cambridge CB2 0RE, UK; b Department of Urology, Addenbrooke's Hospital, Cambridge CB2 2QQ, UK; c Academic Urology Group, University of Cambridge, Cambridge, CB2 0QQ, UK; d Department of Medical Epidemiology and Biostatistics, Karolinska Institutet, Stockholm, Sweden; e Department of Oncology–Pathology, Karolinska Institutet, Stockholm, Sweden; f Nuffield Department of Surgical Sciences, University of Oxford, Roosevelt Drive, Oxford, UK; g Molecular Diagnostics and Therapeutics Group, University College London, WC1E 6BT, UK; h University of East Anglia, Norwich Research Park, Norwich NR4 7TJ, UK; i Department of Pathology, Addenbrooke's Hospital, Cambridge CB2 2QQ, UK; j Prostate Cancer Research Group, Centre for Molecular Medicine Norway, Nordic EMBL Partnership, University of Oslo and Oslo University Hospital, N-0318 Oslo, Norway; k Department of Molecular Oncology, Institute of Cancer Research, Oslo University Hospitals, N-0424 Oslo, Norway; l Prostate Cancer UK/Movember Centre of Excellence for Prostate Cancer Research, Centre for Cancer Research and Cell Biology, Queen's University, Belfast, UK

**Keywords:** Prostate cancer, Risk stratification, Genomics, Prognosis, Gene signature, Biochemical relapse, Personalised medicine

## Abstract

**Background:**

Understanding the heterogeneous genotypes and phenotypes of prostate cancer is fundamental to improving the way we treat this disease. As yet, there are no validated descriptions of prostate cancer subgroups derived from integrated genomics linked with clinical outcome.

**Methods:**

In a study of 482 tumour, benign and germline samples from 259 men with primary prostate cancer, we used integrative analysis of copy number alterations (CNA) and array transcriptomics to identify genomic loci that affect expression levels of mRNA in an expression quantitative trait loci (eQTL) approach, to stratify patients into subgroups that we then associated with future clinical behaviour, and compared with either CNA or transcriptomics alone.

**Findings:**

We identified five separate patient subgroups with distinct genomic alterations and expression profiles based on 100 discriminating genes in our separate discovery and validation sets of 125 and 103 men. These subgroups were able to consistently predict biochemical relapse (p = 0.0017 and p = 0.016 respectively) and were further validated in a third cohort with long-term follow-up (p = 0.027). We show the relative contributions of gene expression and copy number data on phenotype, and demonstrate the improved power gained from integrative analyses. We confirm alterations in six genes previously associated with prostate cancer (*MAP3K7*, *MELK*, *RCBTB2*, *ELAC2*, *TPD52*, *ZBTB4*), and also identify 94 genes not previously linked to prostate cancer progression that would not have been detected using either transcript or copy number data alone. We confirm a number of previously published molecular changes associated with high risk disease, including *MYC* amplification, and *NKX3-1*, *RB1* and *PTEN* deletions, as well as over-expression of *PCA3* and *AMACR*, and loss of *MSMB* in tumour tissue. A subset of the 100 genes outperforms established clinical predictors of poor prognosis (PSA, Gleason score), as well as previously published gene signatures (p = 0.0001). We further show how our molecular profiles can be used for the early detection of aggressive cases in a clinical setting, and inform treatment decisions.

**Interpretation:**

For the first time in prostate cancer this study demonstrates the importance of integrated genomic analyses incorporating both benign and tumour tissue data in identifying molecular alterations leading to the generation of robust gene sets that are predictive of clinical outcome in independent patient cohorts.

## Highlights

• Integrated genomic profiling of 259 men with prostate cancer Cambridge discovery cohort and Stockholm validation cohort
• 100-feature gene set that reliably differentiates five subgroups (iClusters) of prostate cancer
• Prognostic gene signature that predicts relapse-free survival

Studies in other cancers have shown the advantage of integrated genomic approaches in stratifying disease (e.g. Curtis et al., 2012, breast cancer) but such approaches have not yet been undertaken in prostate cancer. In this study we conducted a comprehensive integrated analysis of a Cambridge discovery and Stockholm validation cohort to stratify men into 5 molecular clusters of varying clinical risk based on copy number and gene expression profiling of 100 key genes. The study then showed that we could predict disease relapse based on a refined subgroup of this gene set and showed the superiority of this ‘signature’ compared to other available signatures. The study introduces to the prostate cancer research community 259 men who have been profiled with copy number, transcript expression, TMPRSS2:ERG gene fusion status and tissue microarray data.

## 1 Introduction

Disease stratification based on molecular signatures has aided the management of other epithelial cancers such as breast cancer (Curtis et al., 2012). In contrast, prostate cancer treatment decisions are still based almost exclusively on histological architecture (Gleason score) (Gleason, 1966; Gleason and Mellinger, 1974), prostate-specific antigen (PSA) levels (Catalona et al., 1994) and local disease state (TNM, WHO 2009), without attention to molecular characteristics. However, recent studies show that prostate cancer can be stratified according to molecular signatures (Glinsky et al., 2004; Varambally et al., 2005; Tomlins et al., 2007; Irshad et al., 2013; Taylor et al., 2010). Prostate cancer is the most non-cutaneous common cancer in males in the UK and USA (www.cancerresearchuk.org and www.cdc.gov) and genetic changes associated with aggressive disease, when present in early tumours, herald the onset of early biochemical relapse (Ramos-Montoya et al., 2014). Early treatment of primary prostate cancer is very effective, but it is still difficult to identify those patients who are likely to progress and to treat them appropriately.

Here we describe the comprehensive, integrated analysis of genomic and transcriptomic data from 351 tissue and blood samples from 156 British men, including 125 radical prostatectomy (RP) samples, 118 with matched benign tissue; 64 matched germline DNA; 19 castrate-resistant prostate cancer (CRPC) from channel transurethral resection of the prostate (chTURP) samples, 13 with matched germ-line DNA, and 12 independent samples with benign prostatic hyperplasia (BPH). We identify five distinct molecular profiles for primary prostate cancer that are predictive of biochemical relapse, based on the integrative analysis of transcript levels and somatic copy number alterations (CNAs). These findings hold when castrate-resistant prostate cancers are considered, and further replicate in a separate cohort of 206 samples from 103 Swedish primary prostate cancers with long-term follow-up (Table 1). We further validate our prognostic molecular profiles in a well-established American cohort (Taylor et al., 2010), the only published study of similar size with comparable genomic and clinical data at the time (Suppl. Table 1). An overview of the data generation is provided in Table 2. We describe key genetic changes that stratify men into different risk groups and suggest possible therapeutic and prognostic application for these discoveries.

## 2 Methods

### 2.1 Patient samples

Ethical approval for the use of Cambridge samples and data collection was granted by the local Research Ethics Committee under ProMPT (Prostate Mechanisms for Progression and Treatment) “Diagnosis, investigation and treatment of prostate disease” (MREC 01/4/061). The Cambridge discovery cohort comprised 358 fresh frozen samples from 156 men, including 125 primary prostate cancer from radical prostatectomy (RP) with matched benign tissue, 64 matched germline genomic DNA (gDNA), 19 castrate-resistant prostate cancer (CRPC) from channel transurethral resection of the prostate (chTURP), 13 with matched germline gDNA, and 12 independent benign samples from holmium laser enucleation of the prostate (HoLEP). The Stockholm validation cohort comprised 206 samples of 103 primary prostate cancer with matched germline DNA, as previously described and 99 samples of mRNA (Liu et al., 2013), and was selected for 50% rate of relapse. Comprehensive clinical (diagnostic) data were collected for each cohort, including pre-operative and 6-monthly follow-up PSA, TNM staging and Gleason score (Table 1; Suppl. Table 2). In all cases, biochemical relapse was defined according to European Guidelines as a persistent rise above 0.2ng/ml (Mottet et al., 2014) or triggered salvage radiotherapy.

### 2.2 Histopathology

Cambridge samples were prepared as described (Warren et al., 2013). Relative proportions of benign, epithelial, stromal and tumour cells were determined by consultant histopathologist (AW) (Suppl. Table 2); samples with ≥ 20% tumour and matched non-tumour cores (when available) were included (Yuan et al., 2012). Stockholm samples were similarly assessed (LE), and included with ≥ 70% tumour content.

### 2.3 Genomic processing

Cambridge: gDNA and total mRNA were extracted from tissue samples (Qiagen AllPrep), and gDNA from whole blood (Tepnel). All DNAs were assayed on Illumina HumanOmni2.5–8 M bead chip arrays; 16 samples were also assayed on Affymetrix SNP6 arrays (Aros, Denmark). Stockholm gDNA samples were assayed on Affymetrix SNP6 arrays, as previously described (Liu et al., 2012). All mRNAs were profiled on Illumina HT12 v4 BeadChip arrays.

### 2.4 Expression data analysis

For each cohort, bead level data were pre-processed to remove spatial artefacts, log2-transformed and quantile normalized using the *beadarray* package (Dunning et al., 2007) in Bioconductor prior to analysis. The ComBAT method (Johnson et al., 2007), as implemented in the *sva* Bioconductor package, was used address batch effects in the expression data. Like other microarray technologies, Illumina arrays are known to harbour a large number of probes that do not match their intended genomic location, or map to genomic locations that are not useful for gene expression studies (Barbosa-Morais et al., 2010). Furthermore, including such probes in an analysis can be misleading (Dunning et al., 2010). We therefore restricted downstream analyses to ‘perfect’ probes only (Barbosa-Morais et al., 2010), and whenever a gene-centric analysis was required we chose the probe with the highest Inter-quartile range (IQR) to represent each gene. Probes (genes) were ranked by IQR values, and the 100 most variable probes across expression data were selected for clustering, based on k-means method (see Chalise et al., 2014 for a review on clustering methods), where each observation belongs to the cluster with the nearest mean that best describes that cluster. A linear modelling approach was used to estimate the expression of each probe in the five subtypes, and the set of matched benign samples. Differential expression statistics for the comparison of each subtype to benign were then generated following Bayes' shrinkage of variance (Smyth, 2004).

### 2.5 Copy number analysis

Data were pre-processed and quality checked using ‘Call Rate QC’ (AROS), gender calls from PennCNV (Wang et al., 2007); sample pairings were confirmed using BADGER (Lynch et al., 2012). SNP6 data were analysed with ASCAT (Van Loo et al., 2010), where Cambridge & Stockholm data were mapped to hg19, and Taylor et al. (2010) data were mapped to hg18. Discovery cohort samples included all available tumour and matched benign pairs. Copy number-related figures are coloured accordingly with intensity illustrating degree of gain/loss. CN = 2 is diploid, 0/1 indicates homo- or heterozygous loss; 3/4 indicates hetero- or homozygous gain; Discovery cohort was analysed in OncoSNP (Yau et al., 2010), using only rank1 & 2 calls (out of ranks1–5) for CNA or LOH at each genomic location; this captures larger changes and/or highest confidence. This collapses CN segments to all local genes with the same CN state; OncoSNP reports a separate segment when it sees a gene with a different CN state. All loci altered (CN ≠ 2) in at least 10% of samples were included, so a total of 117 primary RP tumour samples were included in this analysis. Stockholm validation cohort was analysed in ASCAT (Van Loo et al., 2010), with all calls displayed. Genome plots were generated using ggbio package in Bioconductor (Yin et al., 2012). Percentage genomic alteration (PGA) was calculated by summing the number of bases with CN ≠ < 2, and dividing by 3 billion. Only OncoSNP rank1 calls (highest confidence) were included.

### 2.6 Integrative clustering & iCluster comparison

See Supplementary Methods.

### 2.7 Pathway analysis

Gene ontology pathway enrichment was determined using GeneGo MetaCore (Thomas Reuters) and http://www.pantherdb.org/ using default settings and FDR p ≤ 0.05. All genes with log_2_FC ≥ + 1 or log_2_FC ≤ − 1 difference in expression were used in each analysis, to ensure a meaningful number of targets were included for gene-set enrichment analyses, to determine whether any particular pathways or nodes were specific to different clusters.

### 2.8 ERG gene status

*TMPRRS2-ERG* gene fusion status was determined for Cambridge RP samples using a break-apart FISH assay on purpose-made tissue microarrays as previously described (Clark et al., 2008) from tissue cores adjacent to those included in the genomics study — see below. Categories used were based on those with known clinico-pathological associations; when mixed signals were identified, the sample was assigned to the deletion or split category with the most significant clinical implications, according to Attard et al. (2008); i.e. according to increasing order of severity 2 N, N, ESPLIT/2ESPLIT, EDEL/2EDEL.

### 2.9 Study TMA

Single 3 mm cores from the immediately adjacent slice of each patient tumour and benign core used for genomic analysis (RNA and DNA); i.e. if ‘vial 6’ was used for genomic study (based on percentage tumour cellularity), then a core was punched from the parrafin megablock in the region immediately neighbouring this vial on the prostate map (see Suppl. Table 2).

## 3 Results

In previous studies of breast (Curtis et al., 2012) and prostate cancer (Taylor et al., 2010), it has been suggested that either copy number or transcriptomic profiling alone provides superior clinical prediction. We therefore considered these data separately, before carrying out an integrated analysis combining CN data with associated mRNA transcript profiles of gene targets in an eQTL approach.

### 3.1 Copy number profiling

**Fig. 1:**
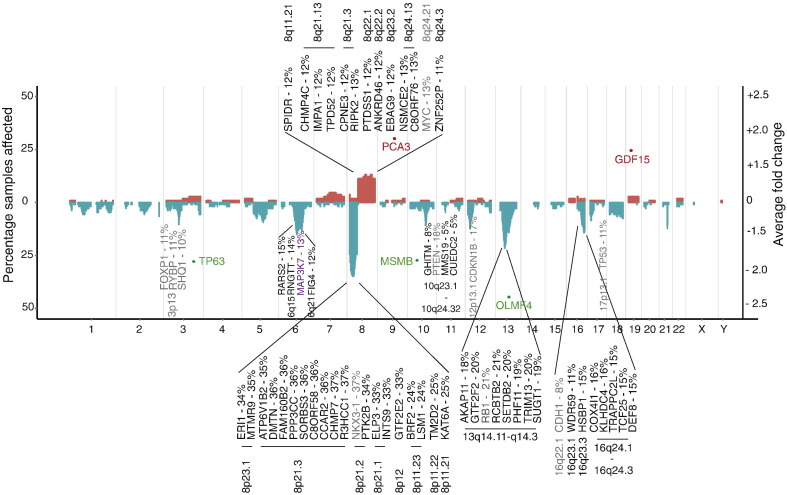
A copy number profile of the prostate cancer genome. The percentage of samples containing copy number aberrations (CNA) at each locus is shown by gain/loss (red/blue); left hand y-axis. Established prostate cancer risk genes commonly disrupted by CNAs (from Williams et al. (2014) meta-analysis) are indicated in grey (gene name and frequency altered in this cohort are shown, see also Suppl. Table 3); only those affected in > 10% samples are annotated. Novel CN changes identified in this cohort (> 10% samples) also in our 100-gene set are indicated in black type. *MAP3K7* is highlighted in purple as the only previously known CN-altered risk gene included in our 100-gene signature. Data were generated on high-density Illumina OMNI2.5 M arrays and analysed using OncoSNP (Yau et al., 2010); only highly stringent calls are shown (see Methods). Chromosome ends are delineated by grey, vertical stripes. Representative genes with large average fold changes (tumours versus matched benign) are shown by red (up-regulation) and green spots (down-regulation); right-hand y-axis. With the exception of *OLFM4* (19%, chr13q14.3), these do not coincide with CN alterations.

The overall genome-wide copy number (CN) profile of the Cambridge (discovery) cohort is consistent with previous findings, with chromosome 8p loss/8q gain evident (Fig. 1). The detection of known prostate cancer risk CN changes (Williams et al., 2014; Sun et al., 2007) in our cohort is broadly consistent with other studies (Suppl. Table 3), with key tumour suppressor *NKX3-1* (8p21.2) deleted in 40% of samples, and *RB1* (24%), *PTEN* (18%), and *TP53* (11%) deletions also easily detected even with very stringent call ranking selection criteria applied (see Methods). A similar overall profile is evident in the Stockholm validation data set (Suppl. Fig. 1), despite being assayed and analysed on different platforms (see Methods). These findings also show that well-established molecular changes in prostate cancer are detectable even at relatively low percentage tumour core content (Table 1).

The prostate cancer genome is dominated by wide-spread deletions: we found 986 genes affected by CN loss, compared to 508 genes with CN gains; only alterations affecting ≥ 10% of the cohort were considered. Indeed, only chromosome 8q harbours frequent gains, involving many genes across the whole region. Most lie outside 8q24, although amplification at *MYC* (13%) was confirmed. In addition to somatic copy number alterations (CNA) in *NKX3-1*, we identified nineteen additional genes across chromosome 8p with at least as many alterations (24–37%). Only genes with CNAs in more than 10% of samples and also relevant in the subsequent integrative analysis are highlighted (Fig. 1). All other genes with CN changes in ≥ 10% of the cohort are listed in Suppl. Table 4.

We confirmed previously identified (Taylor et al., 2010; Lalonde et al., 2014) CNAs in *MAP3K7* (15%; chr6q15), and further refined the original 58 Mb signal across the 6q12–6q22 region, with CNAs identified in *RARS2* (15%) and *RNGTT* (14%) 1- and 2-Mb upstream of *MAP3K7* respectively, as well as CNAs 18.8 Mb downstream, at *FIG4* (12%; chr6q21). In contrast to previous work (Taylor et al., 2010), we did not see any significant correlation with local transcript expression of *RARS2* or *RNGTT* and deletions at *MAP3K*7 locus (see below). In addition to known tumour suppressor *RB1* (24%, chr13q14.2), we identified six other genes with comparable rates of CN change (18–21%), including *AKAP11*, *GTF2F2*, *SETDB2*, *PHF11*, *TRIM13*, and *SUGT1*. These span an 8 Mb region, and are therefore probably distinct signals. Although we confirmed known deletions around *CDH1* (8%, chr16q22.1), these were less frequent than in other studies (Williams et al., 2014; Sun et al., 2007). However, twice as common in this data set were CN losses spanning seven additional genes in 16q23.1–16q24.3 (11-16%) not yet fully characterised, including *TCF25*, *TRAPPC2L*, *DEF8*, *WDR59*, *HSBP1*, *COX4I1*, and *KLHDC4*. Conversely, a frequently deleted region on chr5q near *CHD1*/*APC* (Sun et al., 2007) was not that common in our dataset (38% vs 7%; Suppl. Table 4).

To determine the value of using CN data alone to stratify patients, we clustered primary tumours using all loci altered (see Methods), and identified five patient groups with distinct genomic profiles (Suppl. Fig. 2A). Despite this, clustering on the basis of CN profiling alone was not significant in a survival analysis (logrank p = 0.063; Suppl. Fig. 2B), where time to biochemical relapse (BCR) was used as the primary outcome (see Methods). This is in contrast to previous findings that suggested CN state alone can be a prognostic indicator (Taylor et al., 2010; Lalonde et al., 2014).

### 3.2 mRNA transcript profiling

High levels of variability in gene expression between tumours have recently been shown to be more useful in identifying prostate, breast, colorectal and lung cancer risk genes (Gorlov et al., 2014) than traditional tumour versus normal tissue differential expression approaches, where genuine genes driving aggressive behaviour can be obscured by more abundant but less biologically relevant effects. Furthermore, such inter-tumour variability in gene expression has been shown to have strong association with clinically useful features (Gorlov et al., 2012), such as Gleason score (prostate cancer) and tumour histology (lung cancer). To consider only the most informative genes, we applied k-means clustering to the top 100 mRNA transcript probes with the highest levels of inter-tumour variability in primary prostate cancer tissues. This partitioned the cohort into five patient groups with distinct transcript profiles (Suppl. Fig. 3A; see Methods).

Although this analysis identified known prostate cancer risk genes *AMACR*, *PCA3*, *GDF15* and *MSMB*, as well as several possible candidate genes showing high levels of inter-tumour variability (Suppl. Fig. 3A & Suppl. Table 5), survival analysis showed that transcript-only clustering was not predictive of prognosis (logrank p = 0.11, Suppl. Fig. 3B). In fact, this approach was only informative on inclusion of a small group (n = 6) of outliers which dominated the test statistic (Suppl. Fig. 3C). For completeness, we also assessed sample clustering based on clinical risk factors. As expected, surgical Gleason score was a reliable prognostic indicator (logrank p = 5 × 10^− 6^; Suppl. Fig. 4A), but no other clinical covariate tested was statistically significant (Suppl. Fig. 4B–F). To check our approach, we also considered a traditional fold-change analysis, which confirmed, for example, *PCA3*, *GDF15* and *MSMB* transcript expression as disrupted but unrelated to underlying changes at the DNA level (Fig. 1). We also identified putative tumour suppressor *OLFM4* to have the highest fold-change in expression (all tumour versus matched benign), an effect recently ascribed to CN loss (19% in our data set) and linked to prostate cancer progression (Li et al., 2013).

### 3.3 Integrative analysis identifies distinct patient subtypes with characteristic molecular profiles

Previous studies have shown that most heritable gene expression traits are predominantly controlled by *cis*-acting proximal loci (< 1 Mb), and that these signals are consistently more abundant and stable than more distal *trans* effects (Curtis et al., 2012). For an integrative analysis – combining copy number and gene expression data – we selected features displaying linear correlations between CN state and local transcript expression levels, to identify genome-wide expression quantitative trait loci (eQTL) in the discovery data set (see Methods).

**Fig. 2:**
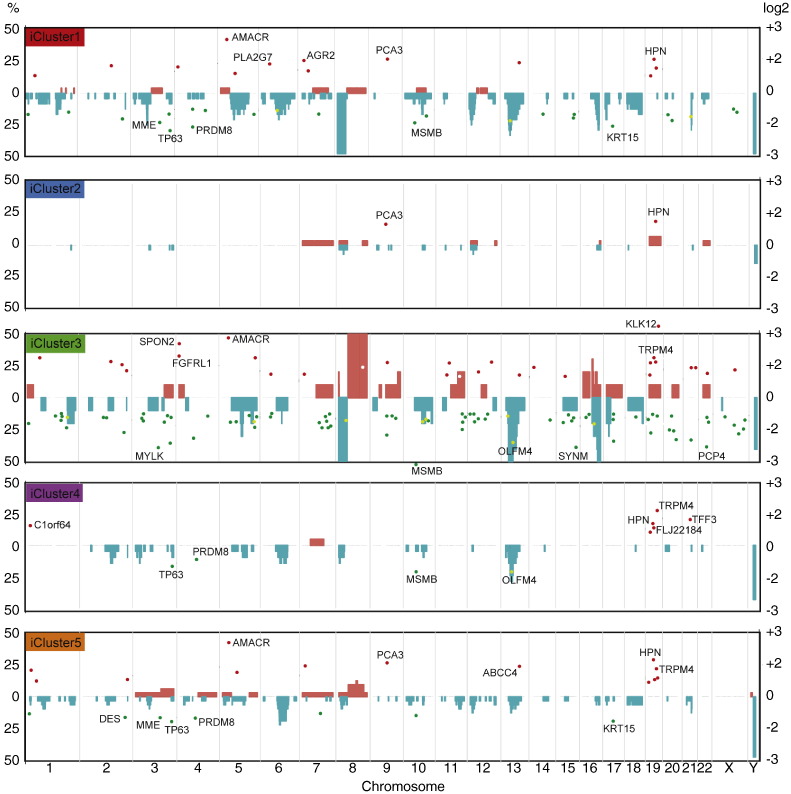
Integrative subgroups have characteristic molecular profiles. Genome-wide frequencies of somatic copy number alterations (CNAs) presented as a percentage of samples (left y-axis) in each integrated Cluster (iCluster). Regions of copy number gain are indicated in red and regions of loss in blue. Subgroups were identified by integrated hierarchical clustering (as described in Methods) of the discovery cohort (n = 125). For the validation cohort (n = 103), men were allocated to these same clusters as described (see Suppl. Fig. 6). Differentially expressed genes (DEG) are superimposed for each cluster; only genes with log2 fold change > 1.5 or < − 1.5 are shown (tumour versus matched benign; right y-axis). The top ten strongest DEGs in each cluster are annotated (see Suppl. Table 8 for full list).

These eQTL features were used in a joint latent variable framework for integrative analysis (iClusterPlus (Mo et al., 2013); see Methods), which identified five distinct molecular subtypes (iCluster1–5) in the Cambridge cohort with characteristic copy number and gene expression profiles (Fig. 2). These were driven by a core set of 100 genes that had both CN and mRNA level changes (Suppl. Table 6). We confirmed this by comparing the results for alternative numbers of clusters (2–11) and features (100 to 1000) (see Suppl. Fig. 5A–C; Suppl. methods). These five clusters (*k* = 4; 100 features) describe 60% of the total observed variance (Suppl. Fig. 5A). These same 100 gene features were used to train a classifier, and partition the Stockholm data set into five patient subtypes with characteristic profiles (Suppl. Fig. 6), similar to those described in the discovery cohort.

**Fig. 3:**
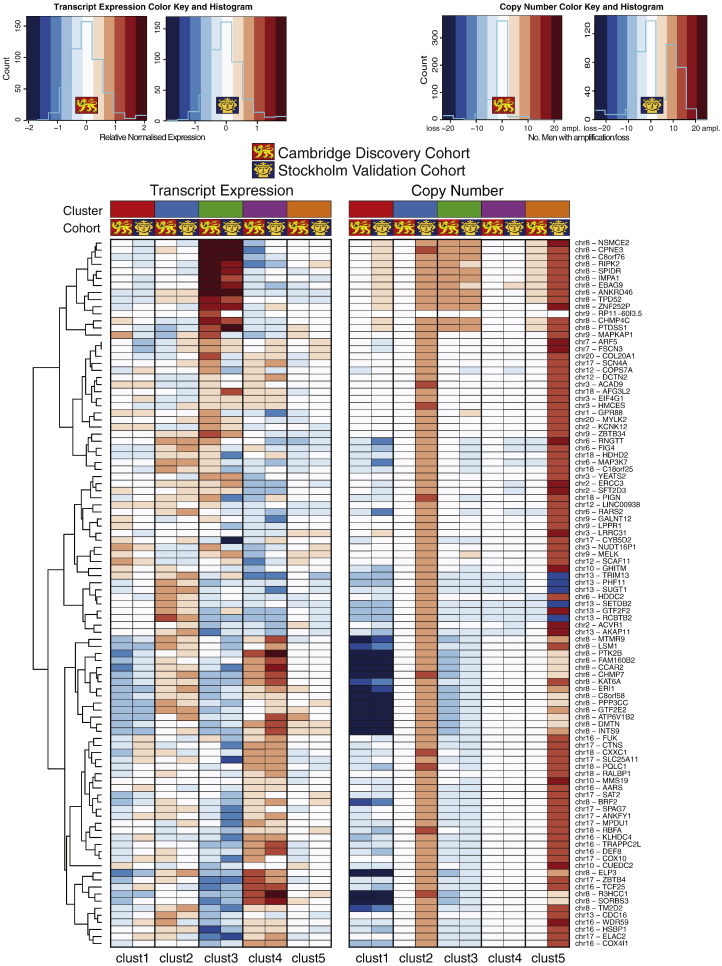
Copy number and expression levels for 100 clustering genes in each integrated cluster. Mean mRNA expression levels are shown as a heatmap for each of the 100 genes used to differentiate the integrated clusters. Copy number is displayed as the number of men with a gain or loss in copies of that gene in that cluster. Chromosome location is also given (see Fig. 2). Scaling as shown.

We assessed transcript and copy number levels for these 100 classifying genes for both the discovery and validation cohorts (Fig. 3). There was clear consistency of expression and copy number aberrations in trained clusters with the exception of subsets of genes in iCluster 2 and 5, which displayed marked copy number amplification in the Stockholm cohort. There was consistent copy number loss and downregulation of expression of genes on chromosome 8 (e.g. *MTMR9*, *LSM1* and *ER1*) in two particular subgroups iCluster 1 and 3, while iCluster 3 was characterised uniquely by copy number gain and upregulation of neighbouring genes on chromosome 8 (e.g. *RIPK2*, *SPIDR* and *IMPA1*). By contrast, iCluster 4 had consistent copy number loss and downregulation of genes on chromosome 13 (e.g. *TRIM13*, *PHF11* and *SUGT1*).

**Fig. 4:**
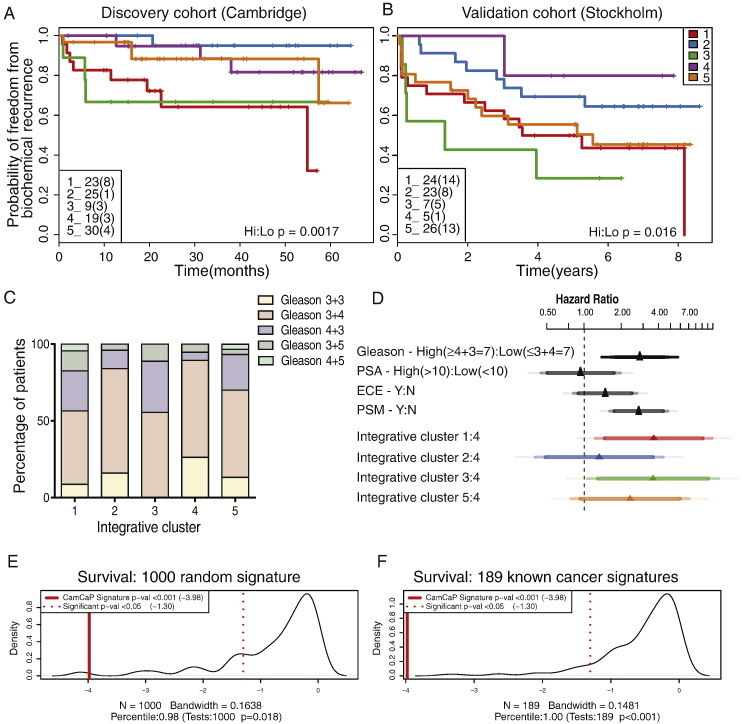
Integrative subgroups have distinct clinical outcomes and are powerful predictors of relapse. A. Kaplan–Meier plot of relapse-free survival over 60 months for the five molecular subtypes in the Cambridge discovery cohort (p = 0.0017 for the two highest versus two lowest risk groups). For each cluster, the total number of samples is indicated (total relapses in brackets). B. Kaplan–Meier plot of relapse-free survival over 96 months in the Stockholm validation cohort (p = 0.016). Further validation was undertaken in a third dataset (Taylor et al. (2010); Suppl. Fig. 9). C. Distribution of Gleason grade across subtypes (Cambridge discovery cohort); no Gleason score predominates in any one subtype (Kruskal–Wallis p = 0.6194). D. Cox proportional hazard ratios with 95% confidence intervals for high vs low Gleason score (≥ 4 + 3 = 7 vs ≤ 3 + 4 = 7), and every other integrative cluster vs best prognosis cluster4. Cambridge and Stockholm datasets were combined to ensure sufficient events per variable (biochemical relapses per cluster) for robust statistical testing (Peduzzi et al., 1995). Confidence intervals shown are 0.9, 0.95 and 0.99. E&F. Refined 100-gene set tested for power to predict relapse in the Stockholm validation set against 1000 random signatures (p < 0.001) and 189 oncological signatures (Subramanian et al., 2005; p < 0.001). Comparison was also made with other prostate cancer signatures (Suppl. Table 11).

Finally, we considered the sample groups identified by our integrative analysis (Fig. 4A) as ‘true’ clusters with clinical relevance, and compared these ‘true’ clusters to the sample groupings suggested by *either* copy number (Suppl. Fig. 2) *or* gene expression data alone (Suppl. Fig. 3). We used two different approaches to determine the similarity of the alternative clustering methods to the ‘true’ clusters. Based on both the *Adjusted Rand Index* (ARI) (Hubert and Arabie, 1985) and the *Variation of Information Index* (VII) (Meilă, 2007), sample clustering based on CN-data is more similar to integrative (‘true’) clustering than is expression-based clustering (Suppl. Table 7; Suppl. methods).

### 3.4 The five subgroups are prognostic in multiple clinical cohorts

Survival analysis showed that these patient subgroups, driven by the 100-gene set, were predictive of outcome in the Cambridge cohort, and clearly distinguished patient groups with better (e.g. blue line; iCluster2) and worse prognosis (e.g. red line; iCluster1), based on BCR-survival data over 60 months (logrank p = 0 · 015; Fig. 4A). Clinical characteristics of the primary tumours in each cluster are given in Suppl. Fig. 7, which also shows the distribution of CRPC samples across the clusters. Tumours with poor prognosis Gleason scores (≥ 4 + 3) are distributed across clusters (Fig. 4C; Kruskal–Wallis p = 0.6194), showing that the molecular subtypes identified are not driven solely by tumour grade. Except for explicable differences in BCR between clusters (chi-squared p = 0.0462), and also extra-capsular extension (chi-squared p = 0.029), these molecular subgroups are not obviously due to other known prostate cancer risk factors (Suppl. Fig. 8), suggesting that these molecular profiles describe additional biological detail that has important prognostic significance.

These five patient clusters were similarly prognostic in our second, novel Stockholm validation cohort (logrank p = 0.048, Fig. 4B), where the extended BCR data (100 months) allowed us to further assess the reliability of the sub-groups in predicting relapse. For example, we found that 56% and 78% of men in iClusters1 and 3 respectively, progressed to relapse disease. Finally, this 100-gene set also replicated in a third, published cohort with similar long-term follow-up (Taylor et al., 2010) (logrank p = 0.027) (Suppl. Fig. 9A; see Suppl. Table 8 & Suppl. Fig. 9B for clinical features of this cohort). Our method assigned 109 patients to clusters with good or poor outcome, similar to the analyses in our discovery (n = 125) and replication (n = 103) cohorts. This further demonstrates our gene set's utility and reproducibility in consistently identifying patient groups.

Finally, we determined Cox proportional hazard ratios (PHR) for Gleason score (≥ 4 + 3 = 7 vs ≤ 3 + 4 = 7), PSA levels (high >10ng/ml *vs* low <10ng/ml), presence of extra-capsular extension (ECE), and positive surgical margins (PSM). We also compared each subgroup to the best outcome cluster (iCluster4) (Fig. 4D), in a combined analysis of the Cambridge and Stockholm data sets, to ensure sufficient events for robust statistical testing (Peduzzi et al., 1995). We found that iClusters1 and 3 identified men with the highest risk of relapse more effectively than either elevated Gleason score (≥ 4 + 3), high PSA, ECE or PSM.

### 3.5 Genomic features of the molecular subtypes identified

In both the Cambridge and Stockholm data sets, the two groups with the best outcomes (iClusters2 and 4) were notable for having relatively few changes in copy number or expression. Conversely, the two groups with poor outcome (iClusters1 & 3) showed significant genomic instability, with large regions of CN gain and loss evident, as well as many more differentially expressed genes (log_2_FC < − 2 or >+ 2) (Fig. 2) in both cohorts. All cluster-specific differentially expressed genes (DEGs) with log_2_FC > 1.5 or < − 1.5 are given in Suppl. Table 9. Notably, dramatic CN changes were not always associated with changes in gene expression level. For example, the marked CN losses and gains at chr8p and 8q respectively, particular to poor-prognosis iCluster3 subtype, were not correlated with marked changes in the expression levels of local genes. Conversely, chromosome 19q harbours multiple genes that were consistently over-expressed in all clusters, but not obviously associated with CN gains. These include *KLK12* (log_2_FC 3.2 iCluster3; Fig. 2) and *HPN* (log_2_FC 1.6–2.2), which have both been linked with prostate cancer aggressiveness (Lose et al., 2013; Holt et al., 2010). Distal gene *FLJ22184* at chr19p encodes a conserved calcium-channel protein most highly expressed in iCluster4 (log_2_FC 1.7), but is as yet largely uncharacterised.

The previously reported eQTL at tumour suppressor gene *OLFM4* (Li et al., 2013) (chr13q) showed no change in CN or associated expression level in iCluster2. However, a clear deletion at *OLFM4* correlated with a marked reduction in transcript levels in iCluster3 (log_2_FC 2.6) and, to a lesser extent, in iClusters1 and 4 (log_2_FC 1.9 and 1.7, respectively). Genes typically associated with prostate cancer are evident: *PCA3* and *GDF15* levels are elevated, and *TP63* and *MSMB* levels are reduced across all tumour subgroups, and are not subtype-specific. However, *AMACR* shows marked over-expression only in the poor outcome iClusters1, 3 and 5 (Fig. 2; Suppl. Table 9). Basal stem cell marker cytokeratin 15 (*KRT15*; chr17q) was also notably down-regulated in these clusters, and may therefore be a useful biomarker of more aggressive disease, as has previously been reported in high-grade squamous epithelial neoplasms (Khanom et al., 2012). In addition, *TRPM4* (chr19q), encoding a calcium-activated ion channel, is up-regulated in all clusters except best-prognosis iCluster2, and was recently identified as a driver gene in the progression to androgen-independent prostate cancer (Schinke et al., 2014), possibly via its role in cell proliferation and β-catenin signalling (Armisén et al., 2011). Loss of *MSMB* expression shows the same pattern – markedly reduced in all but good outcome iCluster2 – consistent with its reported role in prostate cancer (Grisanzio et al., 2012). Principal components analysis (PCA) of gene signatures that may predict early BCR (Ramos-Montoya et al., 2014; Massie et al., 2011; Sharma et al., 2013; Mendiratta et al., 2009) on the Cambridge mRNA data set identified associations between key pathways in prostate cancer and molecular subtype (Suppl. Fig. 10). A summary of probe expression by iCluster is given (Suppl. Fig. 11). Together, these data suggest differences in the expression of transcripts associated with transcription factors such as *AR*, *ERG* or *HES6* (Lamb et al., 2014) within certain prostate cancer subtypes, as well as certain changes in the expression of specific transcripts such as *NKX3*-*1* upregulation (iCluster 5), *CDH1*-up (iCluster 3), cyclin-D1 down (iCluster 2) and *TP53* downregulation (iCluster 3), consistent with previous findings (Markert et al., 2011).

Recent reports have also shown that genomic instability itself – as measured by the percentage of the genome affected (PGA) by copy number changes – is also highly prognostic of PSA recurrence (Taylor et al., 2010; Lalonde et al., 2014; Hieronymus et al., 2014). We therefore determined the PGA for each patient iCluster in both discovery and validation cohorts (see Methods), and found that genomic instability differs significantly between each genomic subtype in both datasets (Suppl. Fig. 12): Kruskal–Wallis p = 4.463 e − 11 (Cambridge); p = 0.0009246 (Stockholm). In each case, iCluster3 showed the highest percentage of genome affected, in keeping with our observations that this subgroup has the most genomic ‘activity’ (Fig. 2).

### 3.6 Gene signature pathway analysis

The 100 gene set includes six protein kinases (*MAP3K7*, *MYLK2*, *RIPK2*, *PTK2B*, *MELK*, *ACVR1*) and five transcription factors (*TRIM13*, *GTF2E2*, *PHF11*, *ERCC3*, *GTF2F2*). Gene ontology analysis revealed pathways relating to RNA and DNA processing, specifically sequence-specific transcription factor and nucleic acid binding, as well as the phosphorylation of proteins were strongly associated with this set (http://www.pantherdb.org/ & Zhang et al., 2005) (Suppl. Fig. 13).

We assessed whether there were any genes in common between our gene set and the top 100 genes with the most variable expression between tumours (presumed therefore to be the most informative in clustering based on expression (Gorlov et al., 2012, 2014) Suppl Fig. 3, Suppl. Tables 5 & 6), but found no overlap. Of the 1493 common CNAs identified in our discovery cohort (Suppl. Table 4), 49 (3.3%) were in our gene set (Suppl. Fig. 14A). We found further limited overlap between our gene set and any other published genes associated with risk in prostate cancer, based either on recurrent CN changes (Williams et al., 2014; 1 gene in common, out of 24), or transcript profiling (Gorlov et al., 2014; 5 genes in common, out of 135) (Suppl. Fig. 14B). As such, our eQTL-based approach has identified 94 additional gene targets that would not have been identified using copy number or transcript profiling data alone.

Within our 100-gene set, only *MAP3K7* (6q15) is known to harbour recurrent CNAs in prostate cancer (Suppl. Fig. 13B & Suppl. Table 3). Deletions in *MAP3K7* have recently been associated with early PSA recurrence, and in tumours that do not contain the *TMPRSS2-ERG* gene fusion, a tumour-suppressor role for *MAP3K7* has been proposed (Kluth et al., 2013). Overlapping the 100-gene set with differentially expressed genes from iCluster4 (best prognosis) and all other sample subgroups combined (all non-iCluster4 DEGs), identified two targets within the 100-gene set with the potential to distinguish subgroup iCluster4 from any other patient subtype with respect to gene expression levels (≥ 2-fold expression change; Suppl. Fig. 14C). These were *CHMP4C* (8q21, CNAs in 12%; 2-fold expression change in cluster3), a chromatin-modifying protein the promoter of which contains transcription factor binding sites for multiple cell cycle control genes, and receptor-interacting protein kinase 2 (*RIPK2*) (8q21, CNAs in 14%, 2.5-fold change in cluster3). Ubiquitinated RIPK2 binds with *MAP3K7*/*TAK1*, translocates to the nucleus and activates a transcriptional cascade involving genes controlling cellular growth, protection against apoptosis and inflammatory response via NF-κB activation (Hasegawa et al., 2008).

Our 100-gene set also showed ‘genomic alteration’ (amplifications, deletions, missense or truncating mutations, and mRNA and protein level changes) in 249/257 prostate cancer samples with complete data at TCGA (the Cancer Genome Atlas; www.cbioportal.org). *CHMP7* and *CCAR2* were the most frequently genomically altered genes in the signature, in 47% and 42% of samples, respectively. *EIF4G1* was also notable in that it was the only gene altered at the protein level in 14% of TCGA prostate cancer samples tested, measured by reverse-phase protein arrays (RPPA), which suggests it may be a useful histological biomarker.

### 3.7 The gene signature is prognostic

We tested the prognostic usefulness of the 100 CN and expression features contained within our integrated gene-set on the Stockholm cohort (Table 1 & Methods). We found that the 100-gene signature showed significant power to separate out a poor prognosis patient group with quicker time to recurrence (chi-squared 0.017) in this large validation cohort (Suppl. Fig. 15A, B). We sought to refine this set further and identified subsets of approximately 50 genes (including copy number and expression features) that best defined each of the five iCluster groups (see Suppl. methods). We selected the most informative subset of genes that predicted biochemical relapse to take forward as a signature (iCluster 4; Suppl. Fig. 16, Suppl. Table 10).

Since there are several cancer gene signatures available, we tested the performance of our refined signature against other signatures. First, we compared it against 1000 randomly selected sets of comparable number of genes (Suppl. methods, Suppl. Fig. 17) (Venet et al., 2011). Our gene signature was in the 98th percentile of performance (p < 0.001), with only 18 out of 1 000 random 50-gene signatures doing better in predicting relapse in the Stockholm data set (Fig. 4E, F). Then we tested how well previously identified oncogenic signatures were able to predict prognosis in our validation set. Initially, we considered 189 known oncogenic signatures from multiple cancer types in MSigDB (Subramanian et al., 2005). A number of the known signatures exhibit prognostic power; however our signature outperforms each one (p < 0.001; Suppl. methods) (Suppl. Fig. 16). Next, we compared our gene signature to previously published signatures for prostate cancer (Irshad et al., 2013; Ramos-Montoya et al., 2014; Lalonde et al., 2014; Sharma et al., 2013; Cuzick et al., 2011) as well as the Oncotype Dx Prostate Cancer assay (see Suppl. Table 11; Suppl. methods) to determine if our genes have prognostic power beyond other possible gene sets. It should be noted that there was limited overlap between the signatures (Suppl. Fig. 18). Our 100-gene signature outperformed all other gene sets in identifying patients with early time to biochemical relapse in the Stockholm cohort (p = 0.0001; Table 3).

## 4 Discussion

We have demonstrated that the integration of copy number and transcript profiling data provides effective risk stratification of men with localised prostate cancer in two novel, distinct cohorts totalling 259 men.

Previous approaches to partitioning samples have concentrated mainly on mRNA biomarkers (Suppl. Table 11). We have clearly shown that combining these two approaches in an eQTL analysis is more powerful in predicting outcome (Fig. 4), as well as in identifying new gene targets also likely to be functionally relevant (Suppl. Table 7). This insight may be particularly helpful to future studies involving large data sets with multiple classes of genomic information, for example the Cancer Genome Atlas (TCGA) or the International cancer Genome Consortium (ICGC) that are relying on ever higher-resolution next-generation sequencing approaches to stratify patients and identify disease-specific driver mutations. In such studies it will be important to determine which genomic modifications are redundant and which are functionally relevant to the disease.

Our prostate cancer gene signature is associated with a distinct set of processes (nucleic acid processing, TF-binding and phosphorylation of proteins) compared to others that relate to cell-cycle control (Ramos-Montoya et al., 2014; Cuzick et al., 2011) or lipid metabolism (Lalonde et al., 2014). Nonetheless, there appears to be scant overlap between prostate cancer gene signatures generally (Suppl. Fig. 17), possibly due to the trend in generating ever smaller gene signatures. Despite this, our refined 100-gene set seems, in our analysis, to be more informative than any other published signature to date (Suppl. Table 12), and as such presents a practically useful, robust tool to help clinicians distinguish good and poor outcome disease. Furthermore, we propose that that it may help tackle the confounding effects of multi-focal heterogeneity highlighted recently in an in-depth whole genome sequencing study of three patients with prostate cancer (Cooper et al., 2015).

Three recent studies have addressed the question of heterogeneity within prostate cancer, identifying multiple foci of differing clonal origin in each case (Cooper et al., 2015; Gundem et al., 2015; Hong et al., 2015) and suggesting that single tissue samplings of a prostate risks missing information about the most important tumour clone. We are unable to address this directly in our study. However, we ensured that our specimens contained a minimum 20% tumour (range 20–90%), and further validated our findings in a more strictly defined, mature validation cohort (Stockholm; ≥ 70% tumour content; mean 78 month follow-up). The robust replication of our initial findings suggests that single sampling is adequate when translated to a large enough cohort of patients. We also had the opportunity to compare matched tumour and benign samples to germline DNA in order to assess field effects (n = 64 triple matched samples). This will, no doubt, be of interest to the aforementioned larger studies (TCGA, ICGC) which also depend mainly on single samples taken from men with prostate cancer. We suspect that additional information will also be provided by sampling of genomic alterations from the cell-free DNA in the blood, and look forward to future publications investigating this approach in prostate cancer (see Murtaza et al. (2013)).

Our data showed that tumours with poor prognosis Gleason sums (≥ 7) were distributed across all five clusters, suggesting that the molecular subtypes identified are not solely driven by histology. Other biomarkers (elevated PSA, *TMPRSS2-ERG* deletion) were also represented (Fig. 3C, Suppl. Fig. 8) across the five patient subtypes. Furthermore, the most powerful subtype as regards prognosis was patient subtype iCluster3, associated with the most marked changes at the molecular level, and predictive of early biochemical relapse. Further analysis of this subgroup for mutations not detectable by copy number or SNP analysis could identify additional, prognostic molecular markers. Importantly, we demonstrate that we can predict disease relapse based on a refined subgroup of our classifying gene set, and show the superiority of this signature compared to other available signatures (Table 3). Nonetheless, we acknowledge the shortcomings of using biochemical relapse as a surrogate for survival. We anticipate that these prognostic clusters will be further tested alongside other parameters as more mature cohorts become available with reliable disease-specific and overall survival data.

Our findings are clinically significant because they will assist urologists in recommending different treatment approaches for those men who are classified as being in low, intermediate or high risk categories according to conventional clinical criteria. To this end, we present the case of a man within iCluster3 subgroup who had low/intermediate risk disease on clinical assessment (Gleason 3 + 4, PSA < 10, T stage < T2c) but who relapsed early (Suppl. material). We propose that in future, men will be assigned membership to molecular groups such as this, and that this profiling will greatly assist their clinical management. Molecular signatures associated with the most aggressive disease will provide a rationale for early adjuvant treatment immediately after prostatectomy, or indeed after initial biopsy when such technologies can be applied to an 18 mm core biopsy. Further functional analyses of the gene targets presented here will also help us to better understand the biological consequences of tumour-associated molecular alteration. As such, we present a triple-matched resource of prostate cancer copy number and expression profiling data, with matched benign tissue and blood, as well as a fully annotated TMA as an invaluable tool for further translational research into the mutational landscapes of primary and castrate-resistant prostate cancers.

## Author contributions

HRA, ADL, JC, RH, ARM, JK, HW conducted experimental work; ADL, VG, NLS, NCS, CSC, HG, DEN were responsible for patient recruitment and sample collection; ADL, JL, HRA, NLS were responsible for clinical follow-up; HRA, ADL, HG, IGM, DEN contributed to study design; AYW, LAE, JC performed histopathology; MJD, SH, SLV, RS, CMM, AGL, HRA, RR performed analyses; HRA, ADL, MJD, RS, wrote the manuscript. All authors read and approved the final manuscript submission.

## Declaration

The authors declare that they have no competing interests.

## Data and material availability

Study data are deposited in NCBI GEO (unique identifier number GSE70770). Associated sample TMAs are available on request.

## CamCaP Study Group

**Co-Chairs** Alastair Lamb and Helen Ross-Adams.

**Tissue Handling and Clinical Data Collection: *Addenbrookes Hospital/University of Cambridge*** David Neal (Principal Investigator), Alastair Lamb, Helen Ross-Adams, Naomi Sharma, Greg Shaw, Satoshi Hori, Ajoeb Baridi, Antonio Ramos-Montoya, Maxine Tran, Karan Wadhwa, Adam Nelson, Keval Patel, Benjamin Thomas, Hayley Whitaker, Jonathan Kay, Hayley Luxton, Nimish Shah, Vincent Gnanpragasam, Andrew Doble, Christof Kastner, Tevita 'Aho, Anne Warren, Beverley Haynes, Wendy Partridge, Elizabeth Cromwell, Asif Sangrasi, Jo Burge, Anne George, Sara Stearn, Marie Corcoran, Hansley Coret, Gillian Basnett, Indu Francis; ***Karolinska Institutet*** Henrik Gronberg (Principal Investigator), Lars Egevad, Thomas Whitington, Johan Lindberg.

**Genomic/transcriptomic/histopathology analysis: *Cancer Research UK Cambridge Institute*** David Neal (Principal Investigator), Ian Mills, Helen Ross-Adams, Alastair Lamb, Mark Dunning, Rory Stark, Charlie Massie, Andy Lynch, Roslin Russell, Silvia Halim, Antonio Ramos-Montoya, Sarah Vowler, Hayley Whitaker, Jonathan Kay, Yinyin Yuan, Oscar Rueda, James Hadfield, Will Howat, Jodi Miller; ***University of East Anglia, Norwich*** Colin Cooper (Principal Investigator), Jeremy Clark, Rachel Hurst, Daniel Brewer; ***Karolinska Institutet*** Henrik Gronberg (Principal Investigator), Lars Egevad, Thomas Whitington, Johan Lindberg.

The following are the supplementary data related to this article.

Supplementary Materials

Supplementary Table 2

## Figures and Tables

**Table 1:** Summary of clinical characteristics of discovery (Cambridge) and validation (Stockholm) cohorts.

	Cambridge				Stockholm	
Primary tumour — RP		CRPC — chTURP		Primary tumour — RP	
n = 125	%	n = 19	%	n = 103	%
Age (years)						
Mean	60.9		72.4		63.9	
Range	41–73		59–93		54–75	
Pre-operative PSA (ng/ml)						
< 4	3	2%	0		7	7%
4–10	87	70%	3	16%	60	58%
> 10	34	27%	16	84%	28	27%
Unknown	1	1%	–		8	8%
Gleason Grade (RP)						
5	–		–		2	2%
6	18	14%	–		20	19%
7 (3 + 4)	76	61%			58	56%
7 (4 + 3)	21	17%	1	5%		
8	8	6%	2	11%	6	6%
9	2	2%	9	47%	9	9%
10	0	0%	2	11%	1	1%
Neuroendocrine	–		1	5%	–	
Small cell	–		1	5%	–	
Ungraded/unknown	–		1	5%	7	7%
Pathology stage						
pT2	38	30%	–		52	50%
pT3a	76	61%	–		28	27%
pT3b	9	7%	–		15	15%
pT4	2	2%	–			
Unknown					6	6%
Follow-up (months)						
Mean	37		–		78	
Range	2–67		–		2–122	
Biochemical relapse	21	17%	–		48	47%
% tumour cellularity						
Mean	52%		65%		tissue selected for ≥ 70%	
Range	20%–90%		20%–95%			
Positive surgical margins	30	24%	–		44	43%
Extra-capsular extension	87	70%	1	5%	43	42%
Metastases	1	1%	2	11%	4	4%
ERG status⁎						
2EDEL	8	6%	–		–	
2ESPLIT	12	10%	–		–	
EDEL	20	16%	–		–	
ESPLIT	17	14%	–		–	
N	64	51%	–		–	
Unknown	4	3%	–		–	

⁎ According to Attard et al. (2008).

**Table 2:** Number and type of tissue analysed by each platform.

Cambridge discovery							Stockholm validation			Total
Platform	Primary (RP)	Benign (RP)	Germline (RP)	Benign (HoLEP)	CRPC (chTURP)	Germline (chTURP)	Platform	Primary (RP)	Benign (RP)	
OMNI2.5 M (CN)	125	118	64 matched 85 total	4	16	13	SNP6 (CN)	103	103	482
HT12 (mRNA)	115	67	–	12	19	–	HT12 (mRNA)	99	–	312
TMA	125	125	N/A	6	12	N/A	TMA	–	–	268
CN & mRNA	115	67	–	4	16	–	CN & mRNA	99	–	301
CN & mRNA &TMA	115	67	–	4	12	–	CN & mRNA &TMA	–	–	198

CN = copy number.

RP = radical prostatectomy.

HoLEP = holmium laser enucleation of the prostate.

chTURP = channel transurethral resection of the prostate.

OMNI2.5 M = Illumina OMNI2.5 M Genotype Beadchip.

SNP6 = Affymetrix SNP6 Genotype array.

HT12 = Illumina HT12 Expression Beadchip.

**Table 3:** Performance of signatures in predicting relapse in Stockholm validation cohort.

Signature	Gene #	Log rank p-value
100-gene set	100	0.0330

iCluster1	32	0.0295
iCluster2	44	0.0185
iCluster3	36	0.0263
iCluster4	50	0.0001
iCluster5	45	0.1560

Sharma et al. (2013)^29^	16	0.1744
Ramos-Montoya et al. (2014)^10^	222	0.1892
Cuzick et al. (2011)^36^	31	0.2631
Irshad et al. (2013)^8^	19	0.8525
Lalonde et al. (2014)^13^	100	0.4953
OncoType Dx	17	0.7323
