# Corrigendum to “Integration of Copy Number and Transcriptomics Provides Risk Stratification in Prostate Cancer: A Discovery and Validation Cohort Study” [EBioMedicine 2 (9) (2015) 1133–1144]

**DOI:** 10.1016/j.ebiom.2017.03.010

**Published:** 2017-03-08

**Authors:** H. Ross-Adams, A.D. Lamb, M.J. Dunning, S. Halim, J. Lindberg, C.M. Massie, L.A. Egevad, R. Russell, A. Ramos-Montoya, S.L. Vowler, N.L. Sharma, J. Kay, H. Whitaker, J. Clark, R. Hurst, V.J. Gnanapragasam, N.C. Shah, A.Y. Warren, C.S. Cooper, A.G. Lynch, R. Stark, I.G. Mills, H. Grönberg, D.E. Neal, on behalf of the CamCaP Study Group

**Affiliations:** a Cancer Research UK Cambridge Institute, University of Cambridge, Cambridge CB2 0RE, UK; b Department of Urology, Addenbrooke's Hospital, Cambridge CB2 2QQ, UK; c Academic Urology Group, University of Cambridge, Cambridge, CB2 0QQ, UK; d Department of Medical Epidemiology and Biostatistics, Karolinska Institutet, Stockholm, Sweden; e Department of Oncology–Pathology, Karolinska Institutet, Stockholm, Sweden; f Nuffield Department of Surgical Sciences, University of Oxford, Roosevelt Drive, Oxford, UK; g Molecular Diagnostics and Therapeutics Group, University College London, WC1E 6BT, UK; h University of East Anglia, Norwich Research Park, Norwich NR4 7TJ, UK; i Department of Pathology, Addenbrooke's Hospital, Cambridge CB2 2QQ, UK; j Prostate Cancer Research Group, Centre for Molecular Medicine Norway, Nordic EMBL Partnership, University of Oslo and Oslo University Hospital, N-0318 Oslo, Norway; k Department of Molecular Oncology, Institute of Cancer Research, Oslo University Hospitals, N-0424 Oslo, Norway; l Prostate Cancer UK/Movember Centre of Excellence for Prostate Cancer Research, Centre for Cancer Research and Cell Biology, Queen's University, Belfast, UK

The author wishes to point out that there is an error of author affiliation in this article, whereby author H. Grönberg should be affiliated to “Department of Medical Epidemiology and Biostatistics, Karolinska Institutet, Stockholm, Sweden”, and not “Academic Urology Group, University of Cambridge, Cambridge, CB2 0QQ, UK”.

In the Introduction, first paragraph, “Prostate cancer is the most non-cutaneous common cancer…” should read “Prostate cancer is the most common non-cutaneous cancer…”

