# Supplementary material for: Integration of copy number and transcriptomics provides risk stratification in prostate cancer: A discovery and validation cohort study
Source: eBioMedicine. 2015 Jul 29;2(9):1133–44. doi: 10.1016/j.ebiom.2015.07.017 (PMC4588396; doi:10.1016/j.ebiom.2015.07.017)
Supplement: Supplementary file 1 — Supplementary Materials [file mmc1.pdf]

INVENTORY OF ALL SUPPLEMENTARY INFORMATION

Integration of copy number and transcriptomics provides risk stratification in prostate cancer: a discovery and validation cohort study

H Ross-Adams[1]*, AD Lamb[1,2,3]*, MJ Dunning[1*], S Halim[1], J Lindberg[4], CM Massie[1], LA Egevad[5], R Russell[1], A Ramos-Montoya[1], SL Vowler[1], NL Sharma[6], J Kay[1,7], H Whitaker[1,7], J Clark[8], R Hurst[8], VJ Gnanapragasam[2,3], NC Shah[2], AY Warren[9], CS Cooper[8], AG Lynch[1], R Stark[1], IG Mills[1,10,11,12], H Grönberg[4]†, DE Neal[1,2]† on behalf of the CamCaP Study Group‡

† * These authors contributed equally to this work.

‡ List of participants and affiliations appears at the end of the paper

1 Cancer Research UK Cambridge Institute, University of Cambridge, Cambridge, CB2 0RE, UK

2 Department of Urology, Addenbrooke’s Hospital, Cambridge, CB2 2QQ, UK

3 Academic Urology Group, University of Cambridge, Cambridge, CB2 0QQ, UK

4 Department of Medical Epidemiology and Biostatistics, Karolinska Institutet, Stockholm, Sweden

5 Department of Oncology-Pathology, Karolinska Institutet, Stockholm, Sweden

6 Nuffield Department of Surgical Sciences, University of Oxford, Roosevelt Drive, Oxford, UK

7 Current address: Molecular Diagnostics and Therapeutics Group, University College London, W1W 7EJ

8 University of East Anglia, Norwich Research Park, Norwich, NR4 7TJ, UK

9 Department of Pathology, Addenbrooke’s Hospital, Cambridge, CB2 2QQ, UK

10 Prostate Cancer Research Group, Centre for Molecular Medicine Norway, Nordic EMBL Partnership, University of Oslo and Oslo University Hospital, N-0318 Oslo

11 Department of Molecular Oncology, Institute of Cancer Research, Oslo University Hospitals, N-0424 Oslo, Norway

12 Movember Centre of Excellence for Prostate Cancer Research, Centre for Cancer Research and Cell Biology, Queen’s University, Belfast, UK

Supplementary Figure 1-18

Supplementary Methods

Supplementary Case Example

Supplementary Tables 1,3-12 (Supplementary Table 2 is a separate file)

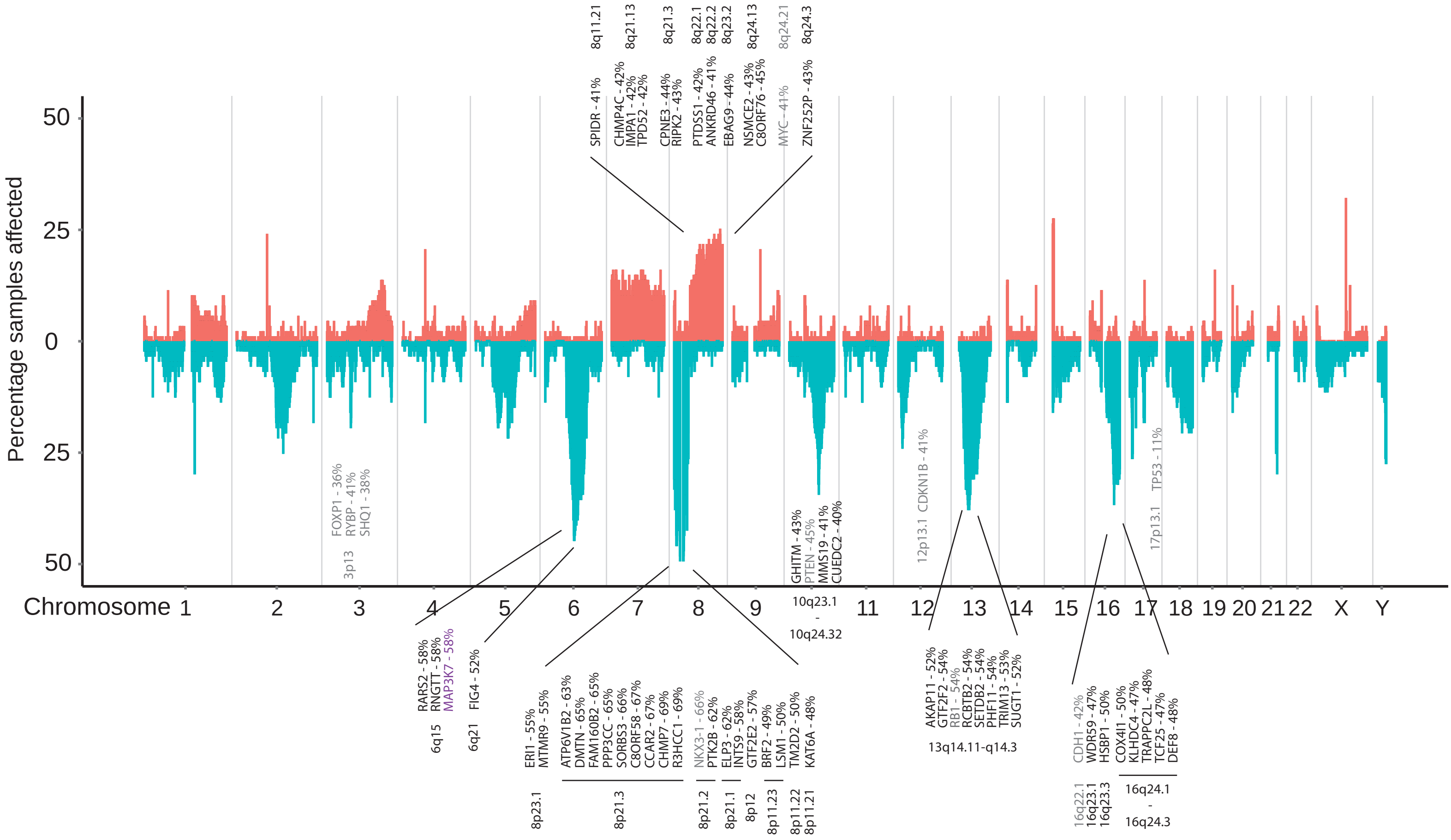

Supplementary Figure 1. A genome-wide copy number profile of the Stockholm validation cohort. The percentage of samples containing copy number aberrations (CNA) at each locus is shown by gain/loss (red/blue); left hand y-axis. Established prostate cancer risk genes commonly disrupted by CNAs (from Williams et al. (2014) meta-analysis) are indicated in grey (gene name and frequency altered in this cohort are shown, see also Suppl. Table 3); only those affected in >10% samples are annotated. Novel CN changes identified in this cohort (>10% samples) also relevant in our 100-gene set are indicated in black type. The same general patterns of gains/losses identified in the discovery cohort are seen in this dataset, which was assayed on lower-density Affymetrix SNP6 arrays and analysed using ASCAT (van Loo et al., 2010) (see Methods), with no filtering of results. Chromosome ends are delineated by grey, vertical stripes. No gene expression data are available for this cohort, as no matched normal tissues were available.

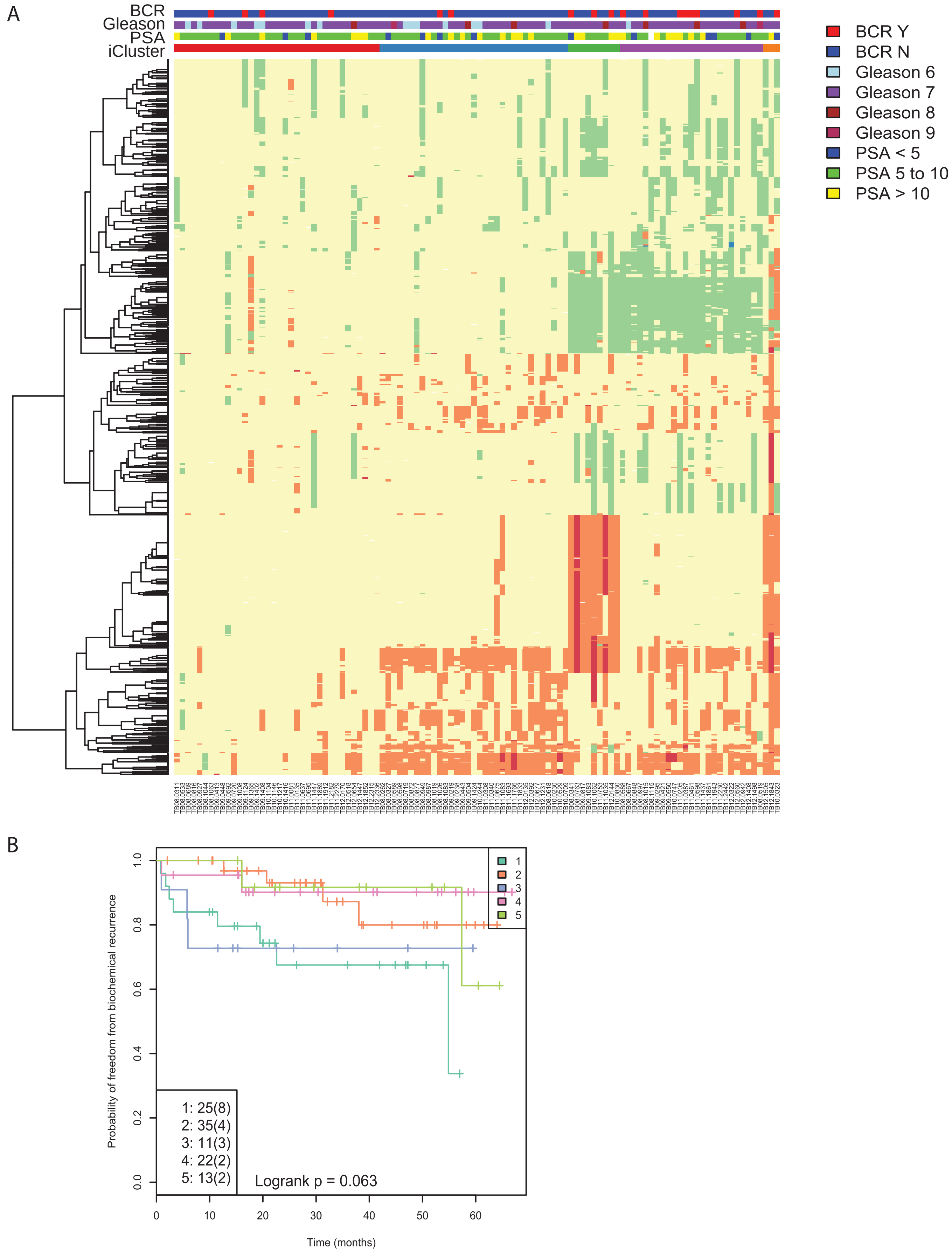

Supplementary Figure 2. **Clustering based on CN alone.**
A. Genome-wide profiles identify five groups of patients with distinct CN profiles (see Methods). Gains are shown in pink (heterozygous; +1) and red (homozygous; +2), losses are indicated in green (heterozygous; -1) and blue (homozygous; -2). The distribution of clinical characteristics is shown. PSA = pre-operative PSA (ng/ml). Gleason = Prostatectomy Gleason Score. BCR = biochemical relapse.
B. Kaplan-Meier biochemical relapse curves using clusters generated above. These CN profiles are not prognostic (logrank p=0.063). Copy number data were analysed in OncoSNP (Yau et al., 2010); all rank calls were used to identify patient clusters to increase signal and ensure even small CNAs were captured (ranks3/4/5) and not just larger copy number alterations (ranks1/2).

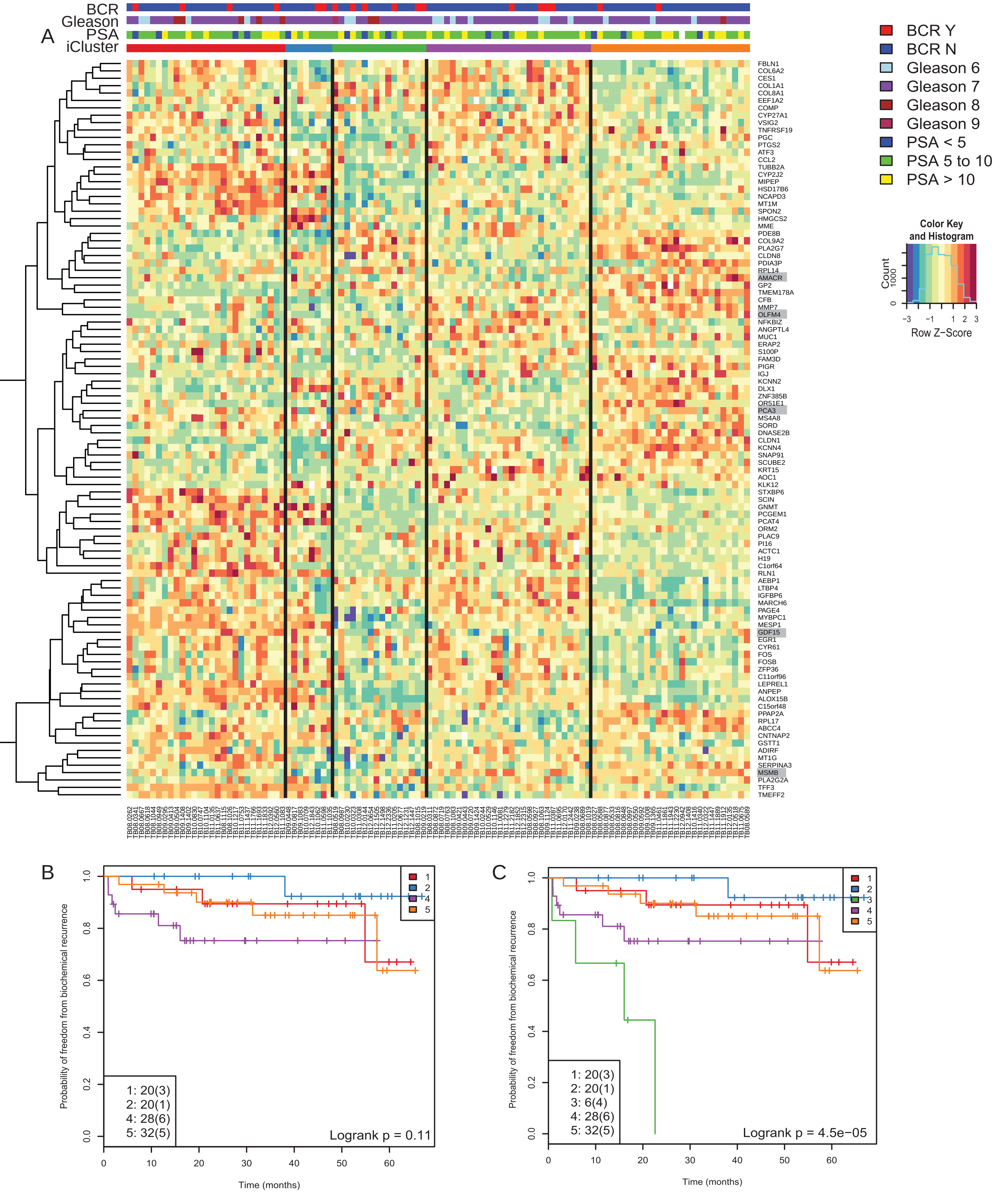

Supplementary Figure 3. Clustering based on Gene Expression (GEX) alone.
A. k-means clustering of the top 100 most variable inter-tumour probes (Gorlov et al., 2014) identified 5 clusters based on Illumina HT12 mRNA transcript data from primary prostate cancer tissue samples (see Methods). The distribution of clinical characteristics is shown. Relative expression of the 100 most variable genes (between tumours) are shown. This approach identified known prostate cancer risk genes AMACR, PCA3, GDF15, OLFM4 and MSMB, highlighted in grey. PSA = pre-operative PSA (ng/ml). Gleason = Prostatectomy Gleason Score. BCR = biochemical relapse.
B. Kaplan-Meier biochemical relapse curves using clusters generated above. These GEX profiles alone are not prognostic (logrank p=0.11).
C. Transcript-only profiling was only statistically significant when a small group of outliers was included in the analysis.

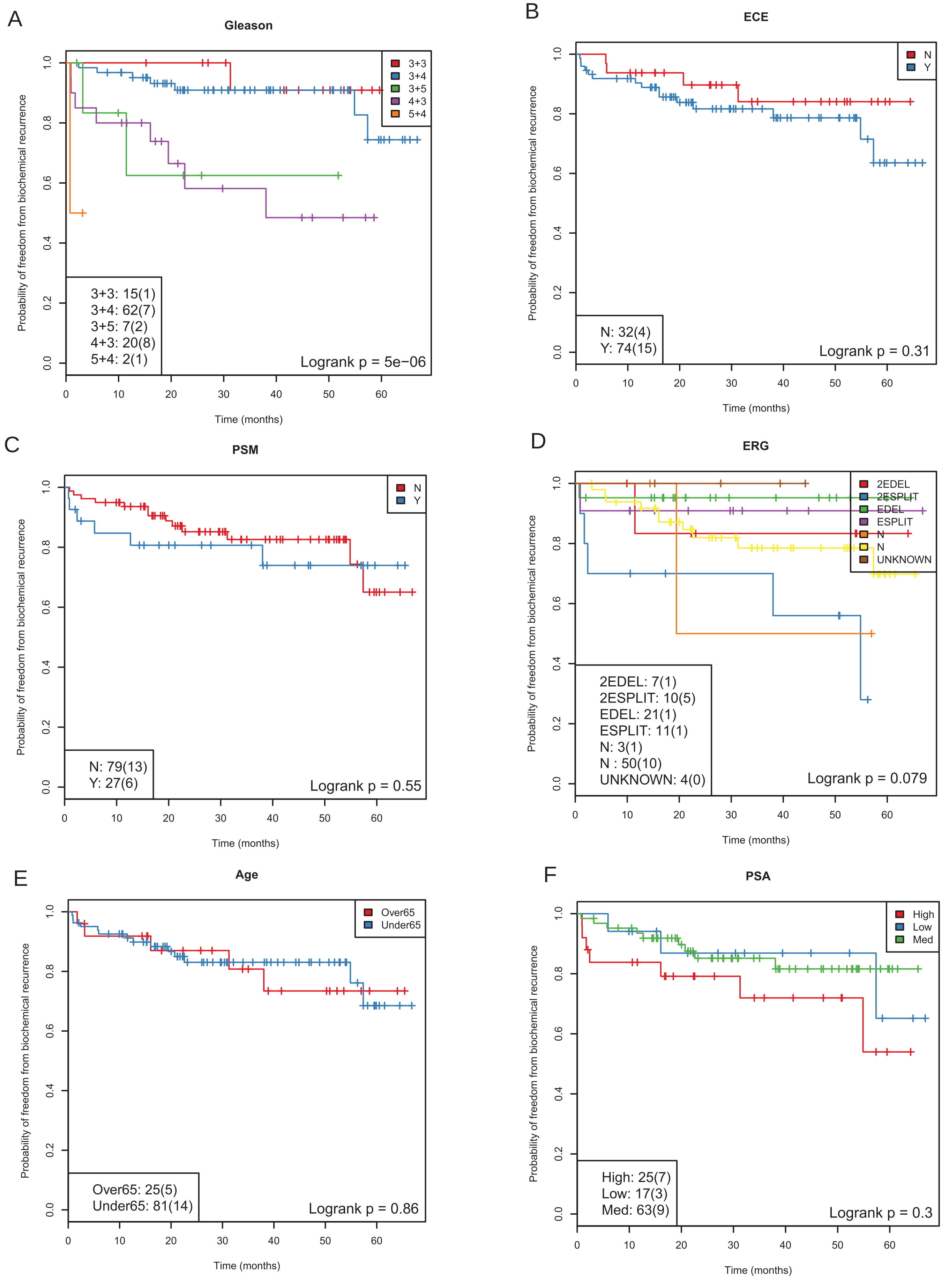

Supplementary Figure 4. Relapse-free survival curves based on clinical characteristics.
A. Kaplan-Meier biochemical relapse curves split by Gleason score in the Discovery Cohort.
B. Extracapsular extension (ECE).
C. Positive surgical margin (PSM).
D. TMPRSS2-ERG gene fusion status (ERG).
E. Age.
F. Pre-operative PSA (low < 3 ng/ml; medium 3-10 ng/ml; high > 10 ng/ml).

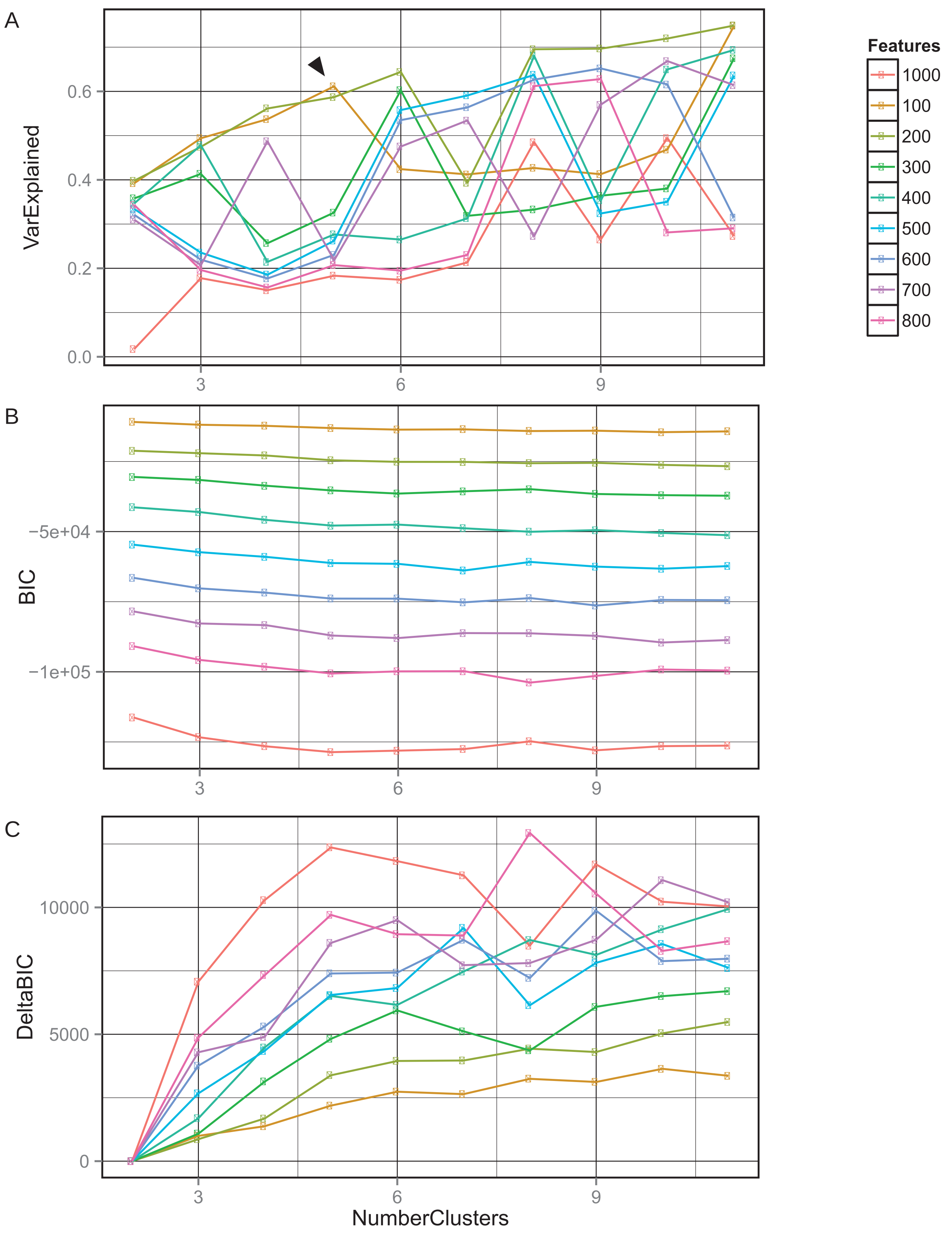

Supplementary Figure 5. Selection of integrated clusters in the Cambridge Discovery Cohort.
A. Variance plotted against number of clusters for 10 increasing size feature sets. The smallest number of features (n=100) consistently described the most variance for lower numbers of clusters. The inflection point at n=5 clusters was chosen as describing the most variance with largest numbers of men per cluster (k+1; iCluster+ k=4; see Methods & Supplementary Methods).
B. Optimal number of features was selected to minimise Bayesian information criterion (BIC) for a given k.
C. One over BIC was plotted (Delta BIC) to better display incremental differences in BIC. Clear inflection is seen in this figure at 5 clusters.

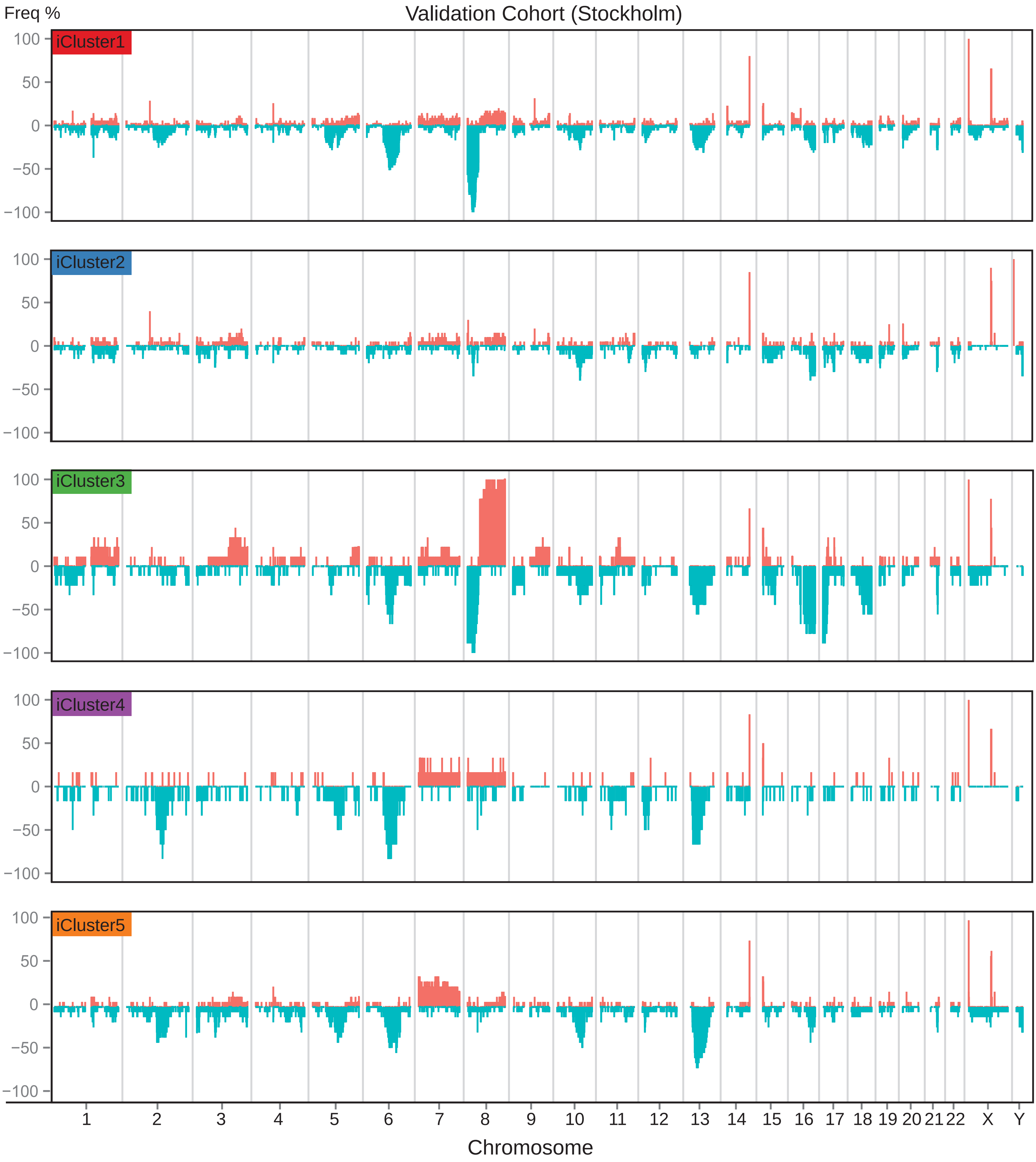

Supplementary Figure 6. Validation of integrative clusters (Stockholm Validation Cohort)
Genome-wide frequencies of somatic copy number alterations (CNAs) presented as a percentage of samples (left y-axis) in each Integrated Cluster (iCluster). Regions of copy number gain are indicated in red and regions of loss in blue. Subgroups were identified by integrated hierarchical clustering (as described in methods) of the discovery cohort (Figure 2; n=125; Illumina Omni2.5 array). For the validation cohort (n=99; Affymetrix SNP6 array) men were allocated to these same clusters based on the 100 CN and GEx features analysed using PAMr (Tibshirani et al., 2003; see Methods). Integrative clusters 1-5 correspond with survival analysis curves in Figure 3B.

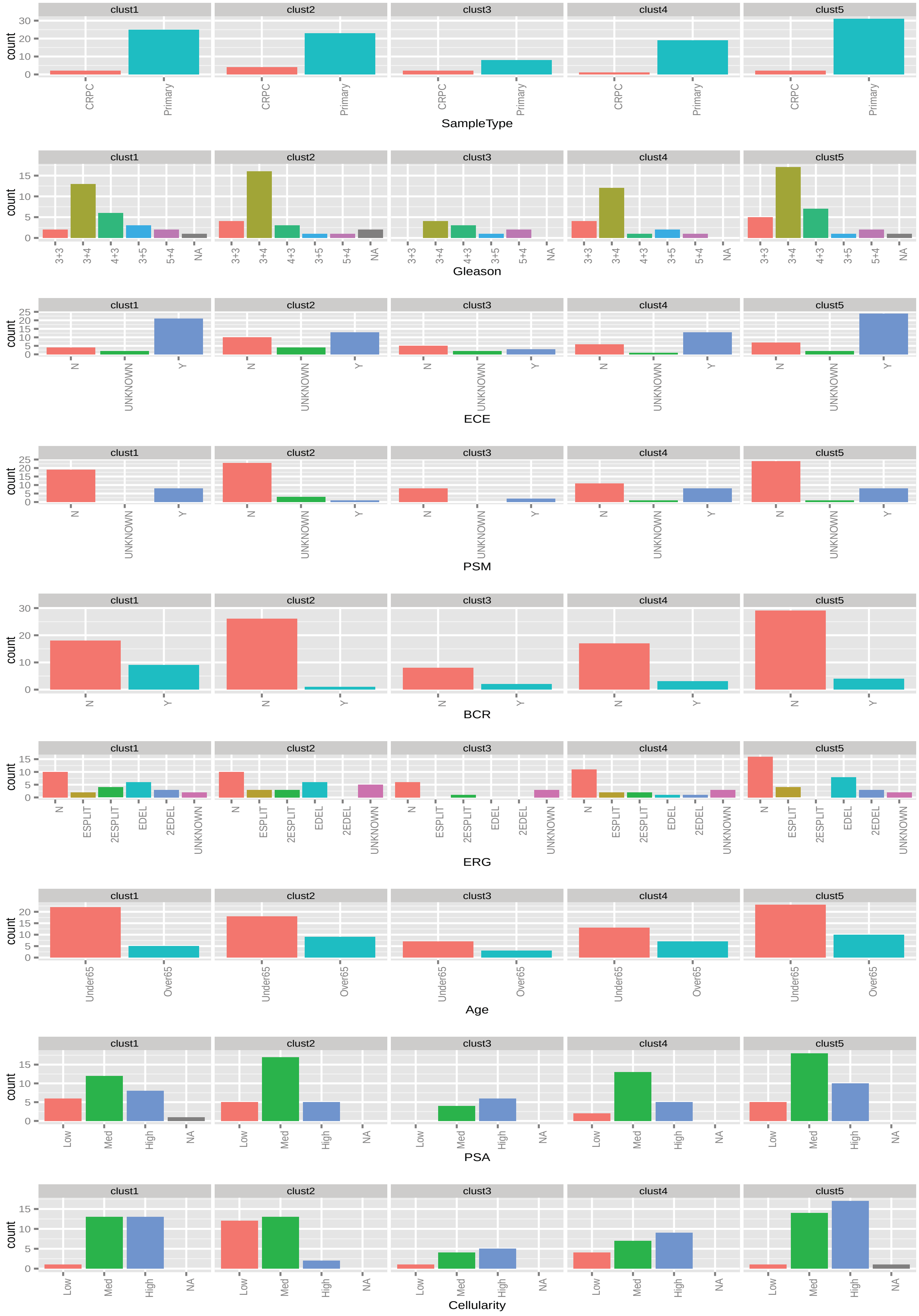

Supplementary Figure 7. Clinical Features of the integrative clusters (Cambridge Discovery Cohort). Distribution of samples between each sub-group is shown. CRPC samples are also included in the distribution (though not used in defining the clusters). Additional clinical information includes the presence of extra-capsular extension (ECE), positive surgical margin (PSM), biochemical relapse (BCR), TMPRSS2:ERG fusion status (ERG), age, PSA (low = <3ng/ml, med = 3-10ng/ml, high = >10ng/ml) and cellularity of the sample analysed.

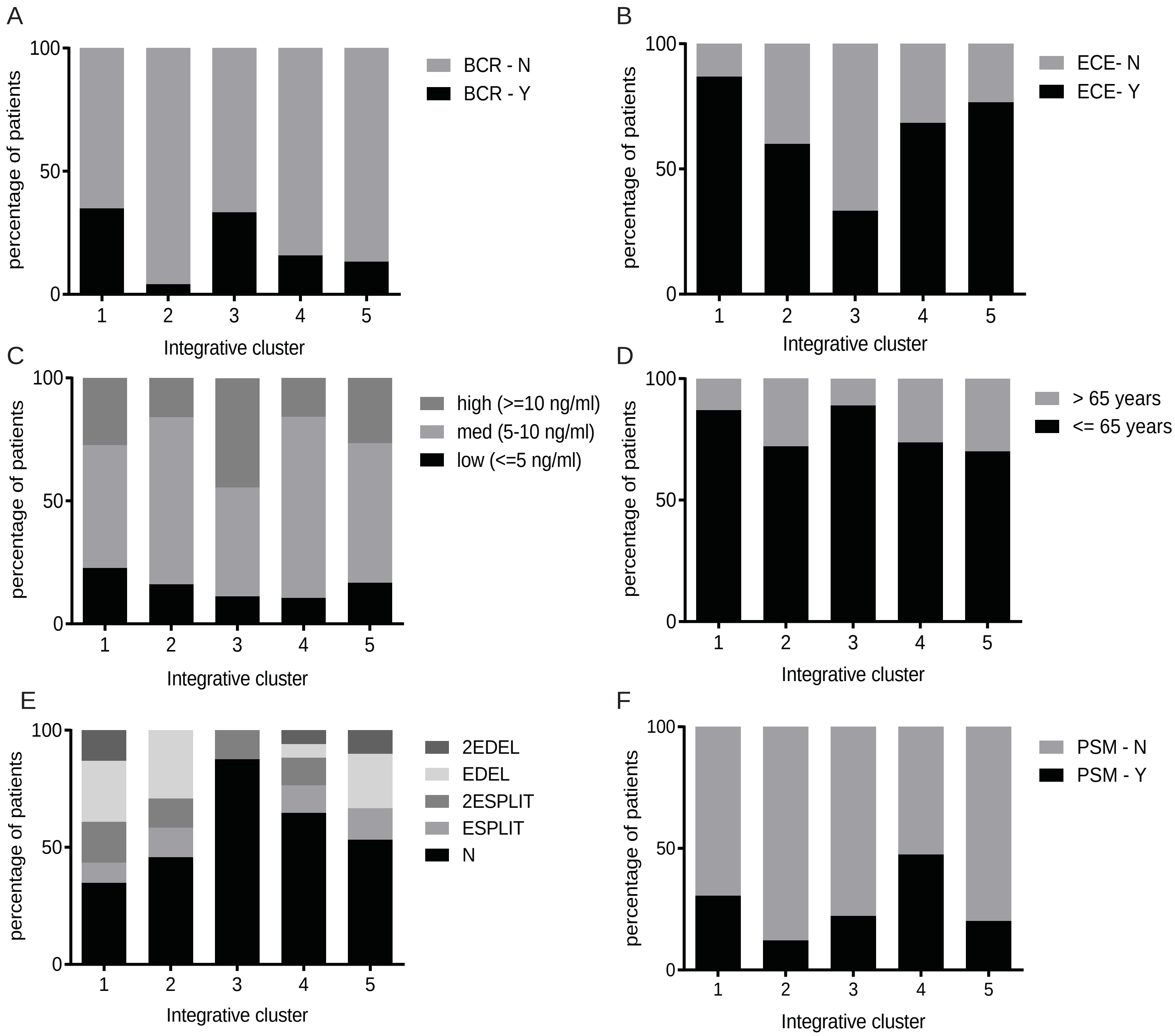

Supplementary Figure 8. Additional display of clinical features across integrative clusters (Cambridge Discovery Cohort).
A. Biochemical relapse (chi-sq p=0.0462).
B. Extra-capsular extension (chi-sq p=0.029).
C. Diagnostic PSA (Kruskal-Wallis p=0.2529).
D. Age (chi-sq p=0.5202).
E. TMPRSS2-ERG deletion status (K-W p=0.2751).
F. Positive surgical margins (chi-sq p=0.0912).

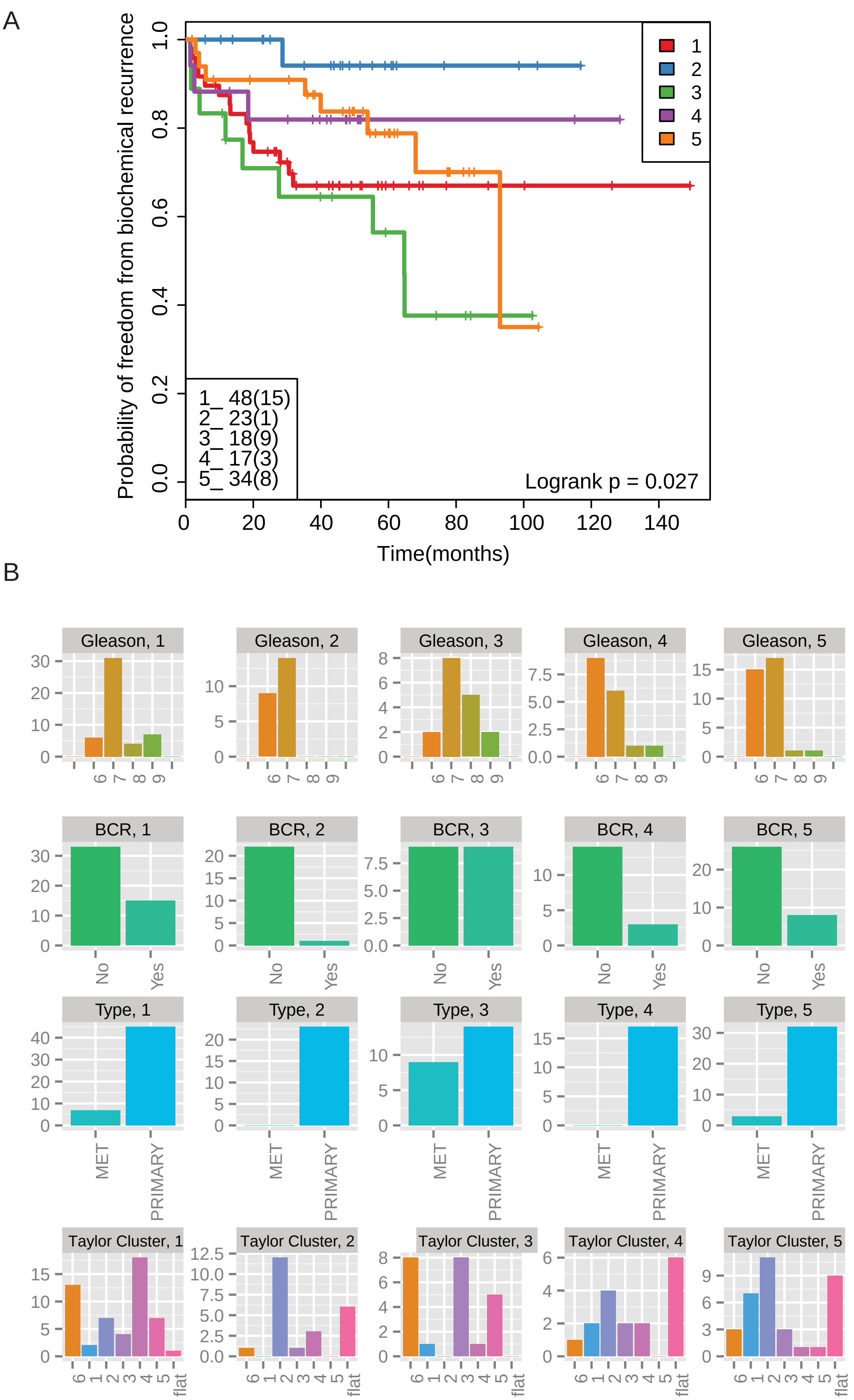

Supplementary Figure 9. **Further validation of integrative clusters in predicting biochemical relapse**.
A. The five molecular subtypes identified in the discovery cohort were assessed in this third validation set with 120-month follow-up (p=0.027; Taylor et al, 2010; see Supplementary Table 7 for clinical features of this cohort).
B. Clinical features of each subgroup are shown.

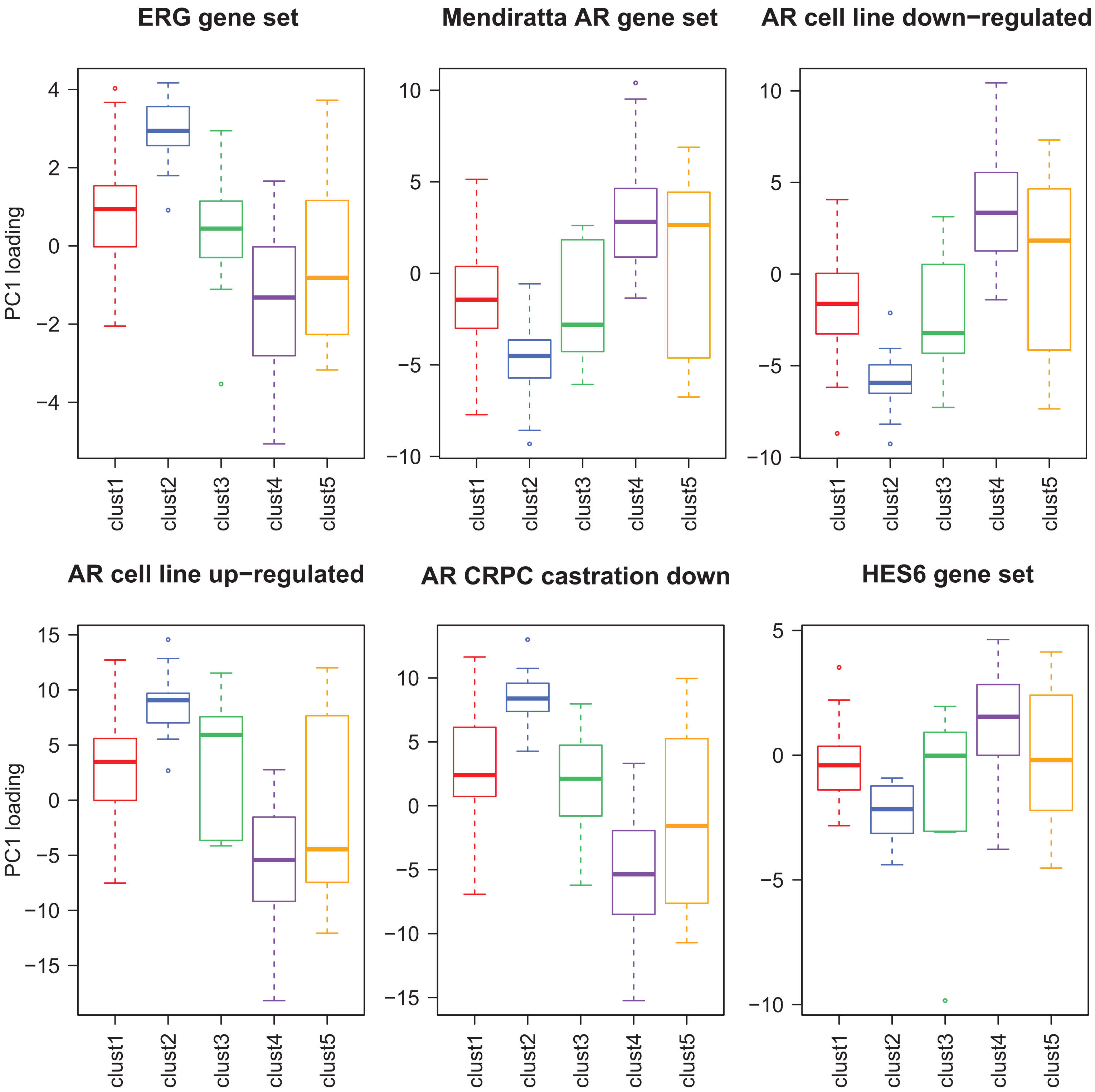

Supplementary Figure 10. Integrated Clusters showed distinct expression of ERG, AR and HES6 gene sets.
Principal components analyses (PCA) on the mRNA data from the Cambridge Discovery Cohort assessing the predominance of published gene sets in different integrated clusters (iCluster). Gene sets were identified from the following: ERG: Massie et al., (2010); Mendiratta et al. (2009), AR cell line up- and down-regulation: Massie et al., (2012); AR CRPC castration down (Sharma et al., 2013) and HES6 (Ramos-Montoya et al., 2014).

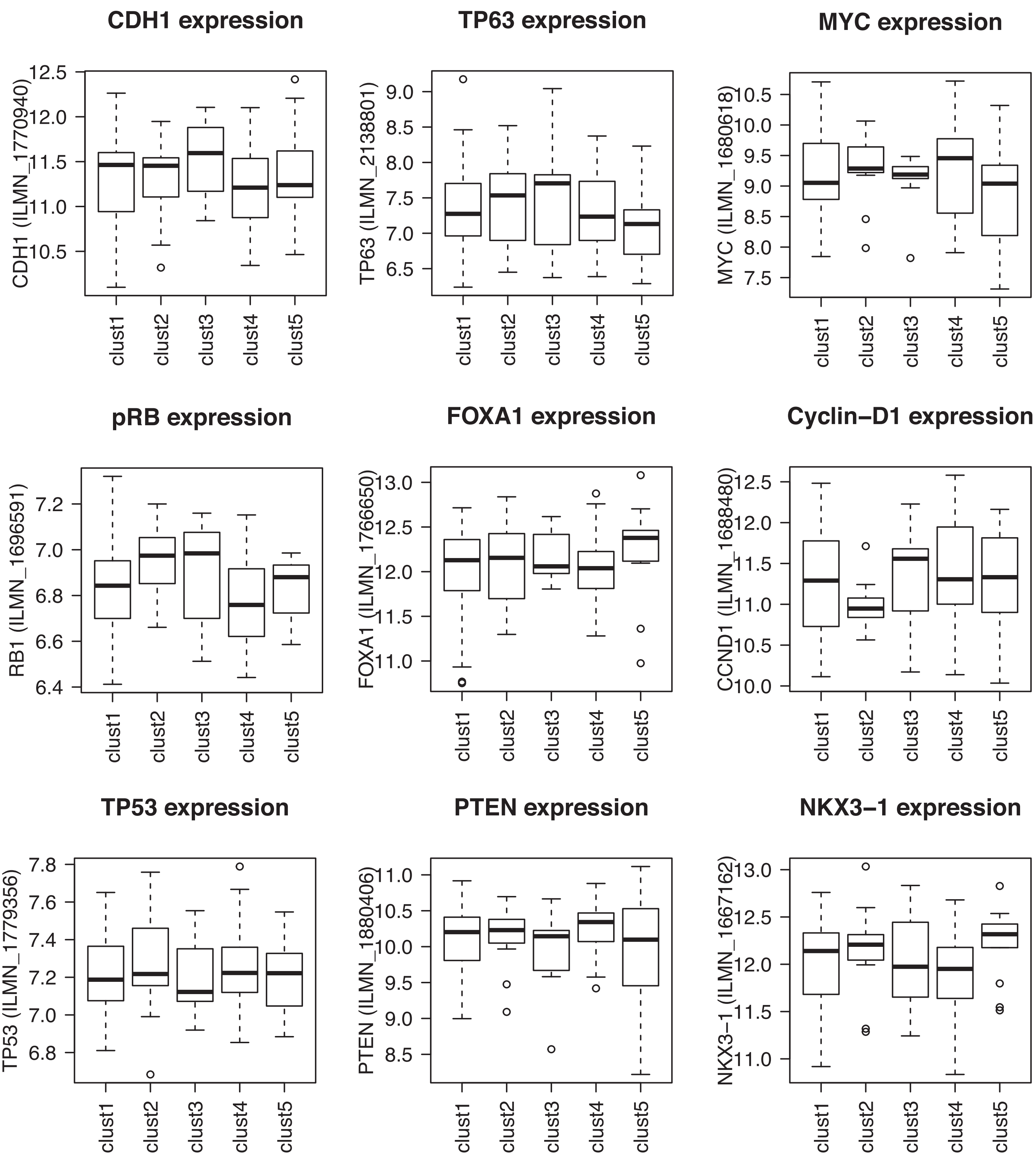

Supplementary Figure 11. Integrated Clusters showed distinct expression of individual genes. Relative expression levels of key prostate cancer genes in each cluster in the Cambridge Discovery Cohort

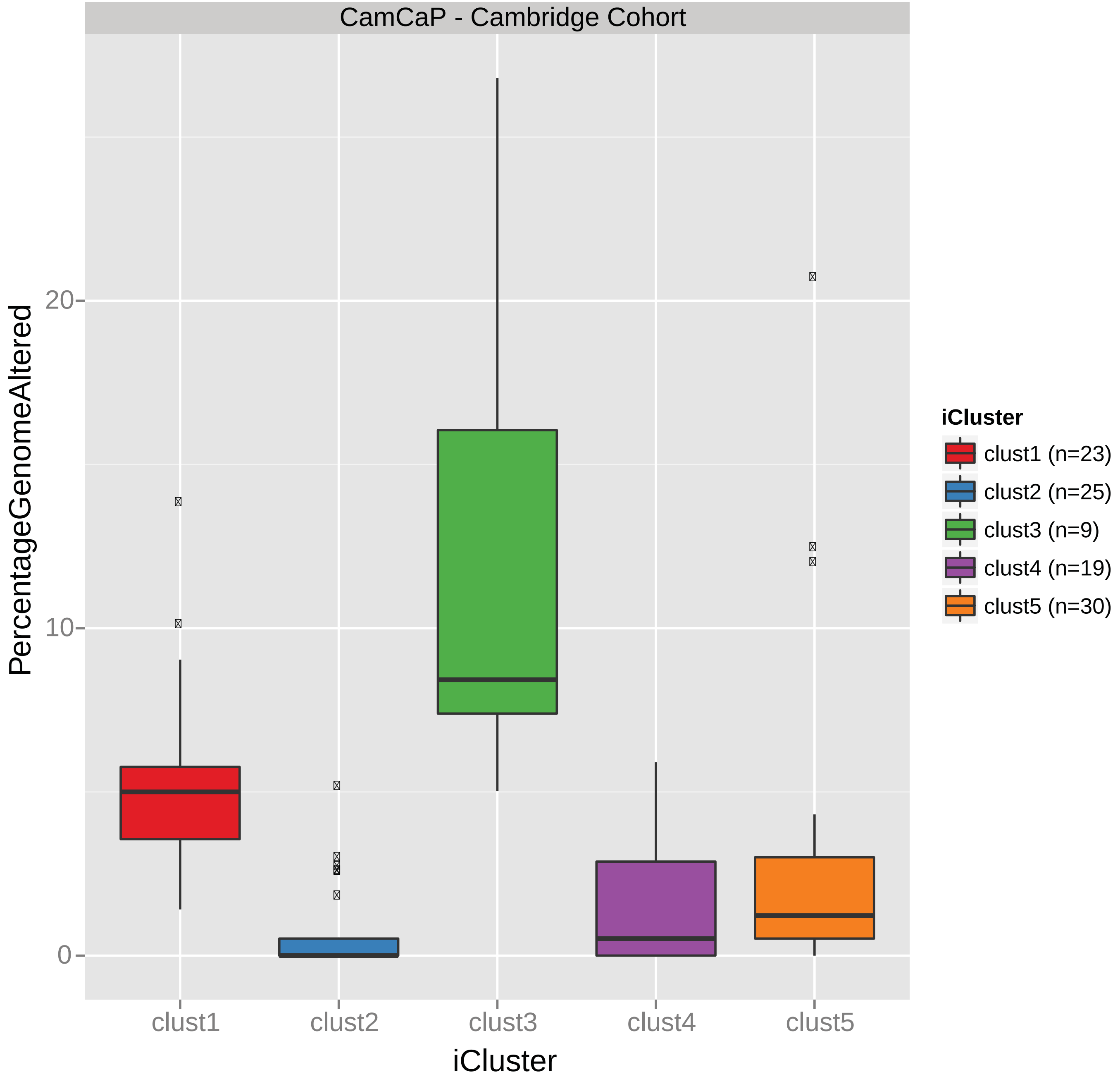

Supplementary Figure 12. Percentage Genome Affected (PGA).
The percentage of the genome affected by copy number change was calculated and mean value plotted for each cluster.

A

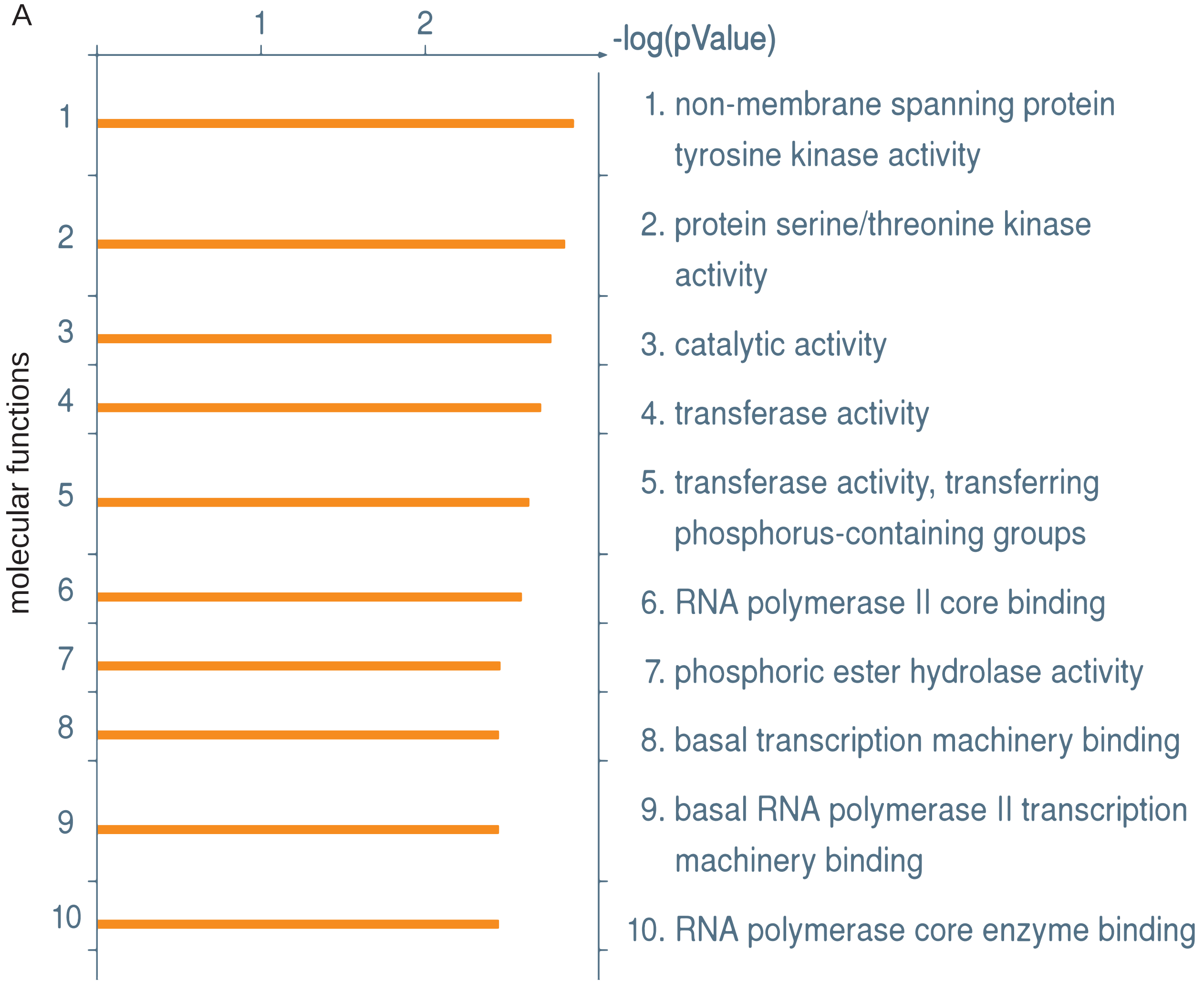

B

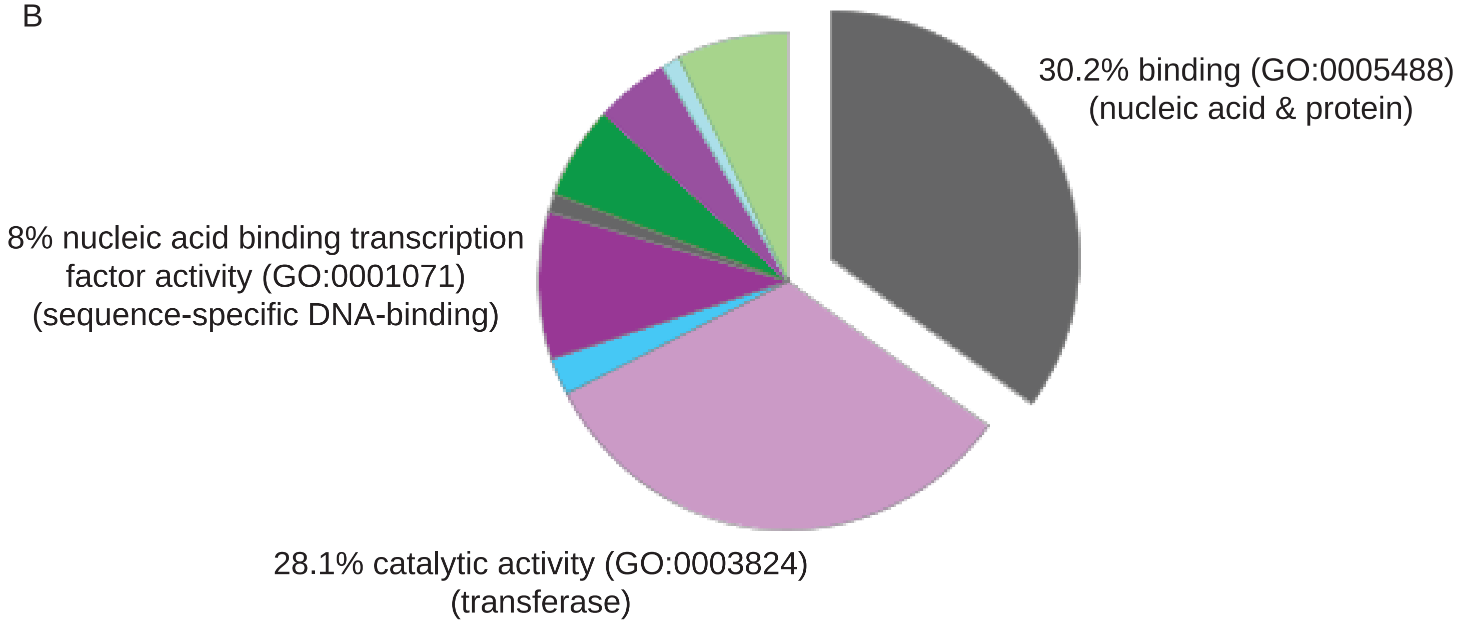

Supplementary Figure 13. Gene ontology pathway analysis of the 100-gene set
A. GeneGO MetaCore (Thomas Reuters) gene ontology analysis.
B. PantherDB (www.pantherdb.org) gene ontology analysis.
Both analyses identified enrichment of pathways relating to RNA and DNA processing – specifically sequence-specific transcription factor and nucleic acid binding – and the phosphorylation of proteins (particularly serine/threonine kinases) in this gene set.

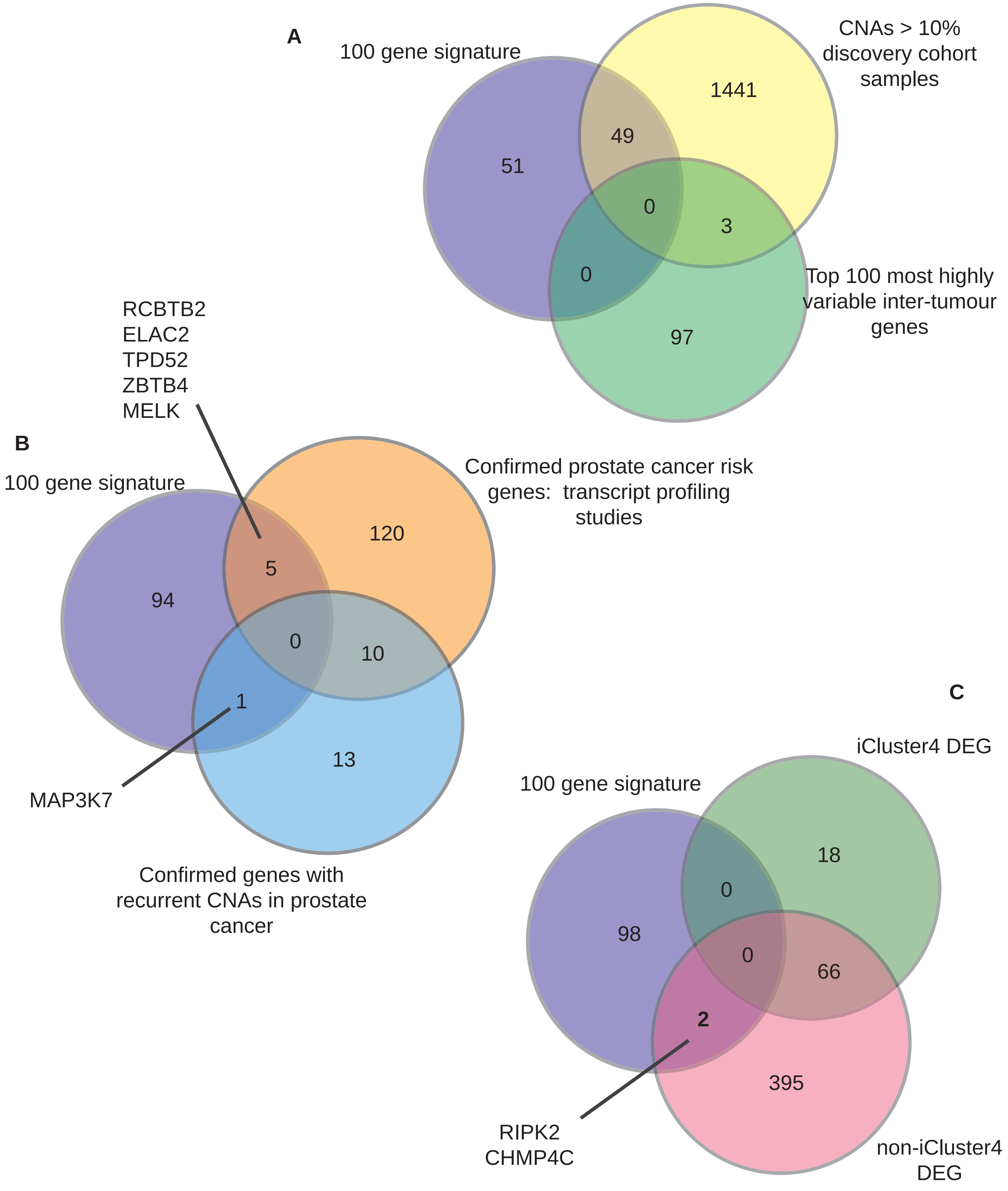

Supplementary Figure 14. Comparison of 100-gene integrative gene set with known CN and gene expression loci.

A. Within the Cambridge Discovery Cohort, only 3.3% (49/1490) of all CN changes (>10 % samples tested) were found in the 100-gene set. None of the top 100 most variable inter-tumour genes was included.

B. Only six risk genes from the 100-gene set have previously been described according to published meta-analyses – CN changes in MAP3K7 and gene expression changes in RCBTB2, ELAC2, TPD52, ZBTB4 and MELK. Our eQTL-based gene set therefore identifies 94 targets that would not have been identified using copy number or transcript profiling data alone.

C. Comparison of differentially expressed genes between iCluster4 and non-iCluster 4 identified CHMP4C and RIPK2 in the 100-gene set that could separate iCluster4 from non-iCluster4 using expression levels alone.

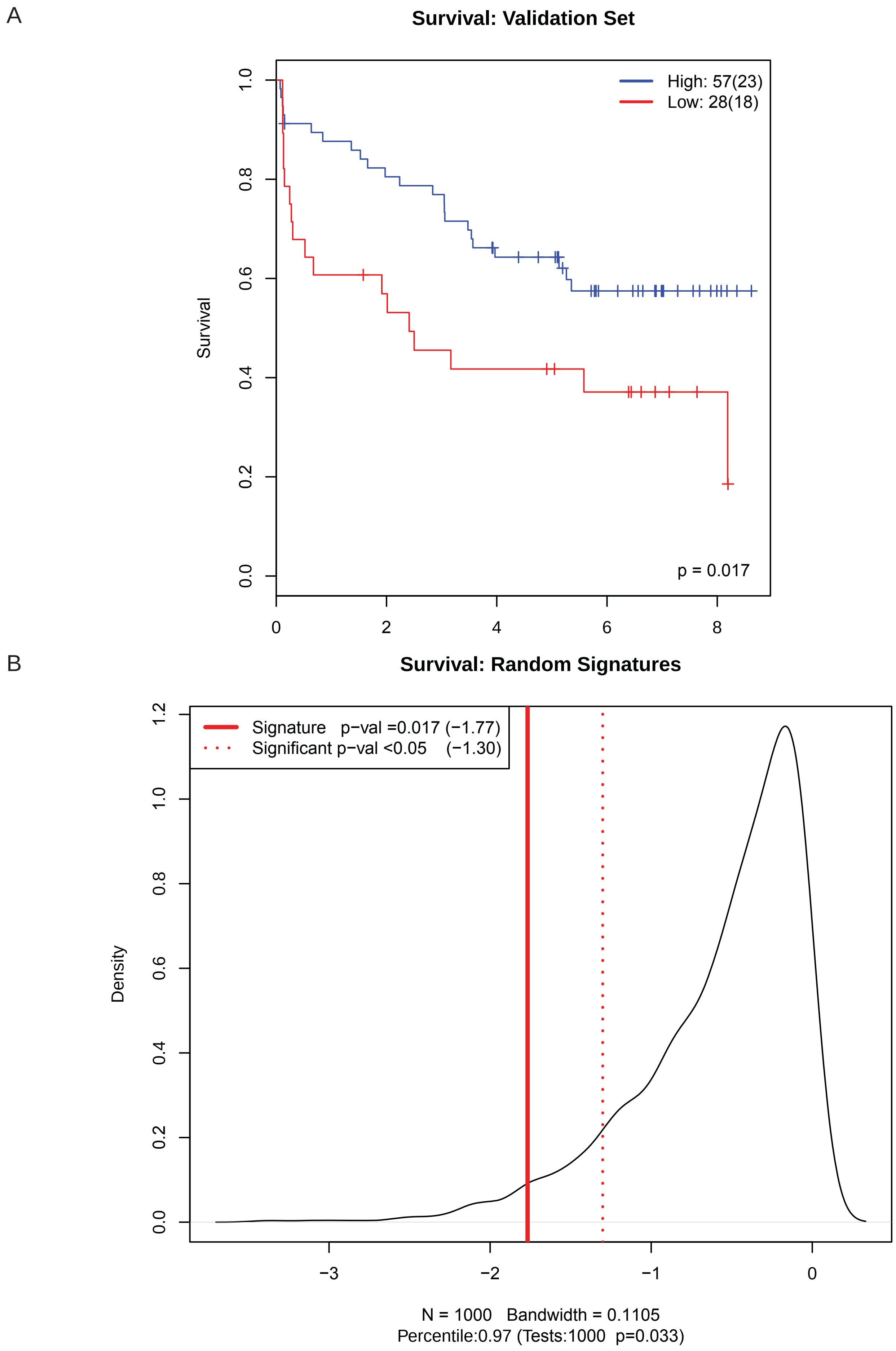

Supplementary Figure 15. Performance of 100-gene set as predictive signature in Stockholm validation cohort.

A. Kaplan-Meier plot of biochemical relapse-free survival in the Stockholm validation cohort over 96 months. Patient data were divided into two categories by calculating the mean value over all features for each sample; the 'high' enriched group comprised those samples >33rd percentile, while the 'low' enriched group consisted of samples ≤ 33rd percentile. Log-rank p-value = 0.017.

B. The 100-gene set was compared to 1 000 random signatures of the same size. The solid red vertical line shows the performance of the Cambridge signature, while the dotted red vertical line shows p = 0.05.

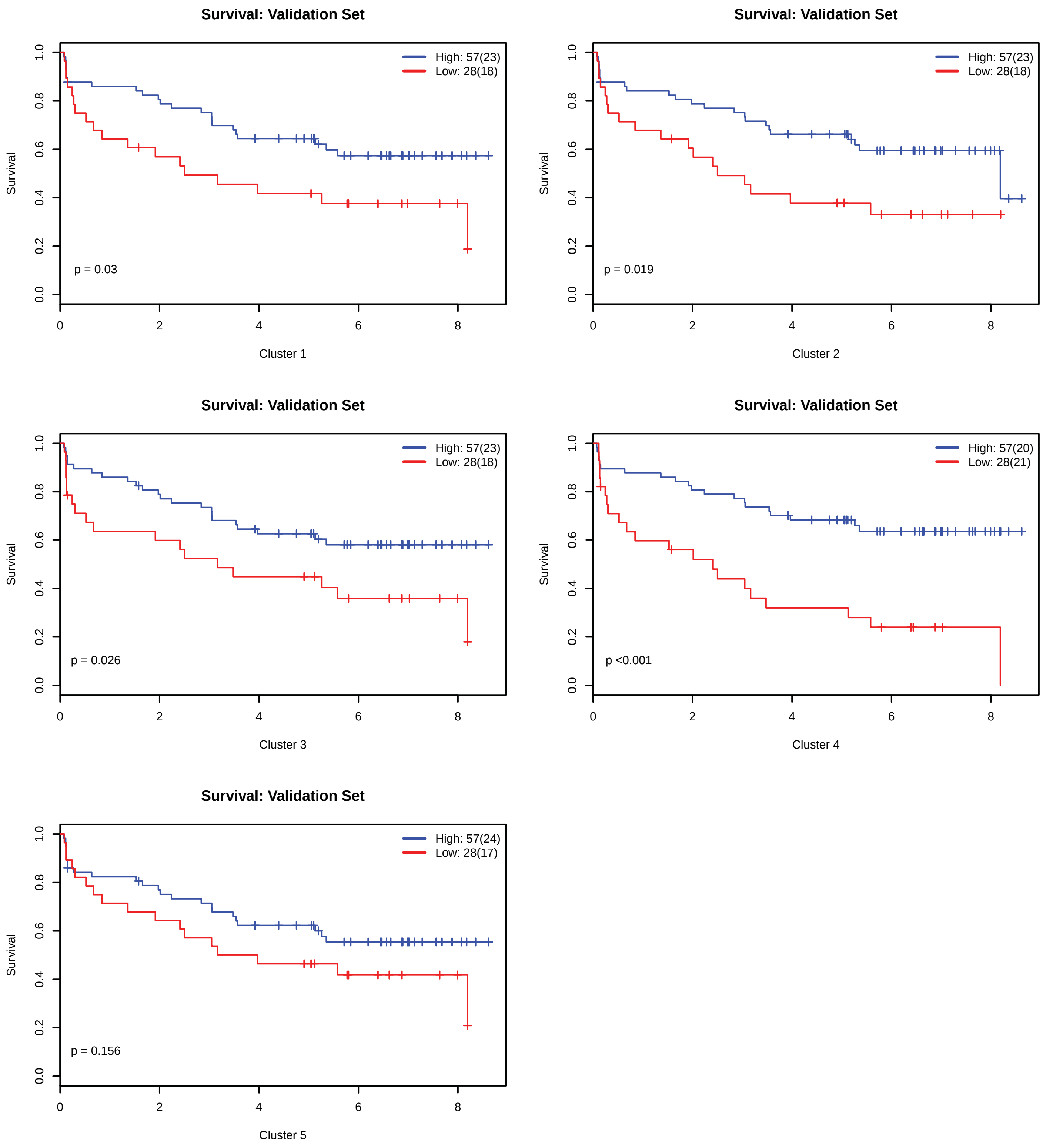

Supplementary Figure 16. Relapse-free survival curves for cluster-specific signatures
Kaplan Meier plots for each of the five clusters, using the 50-feature signatures from rankings computed by PAMR (Tibshirani et al., (2003), applied to the Stockholm validation set with no a priori clustering (see Suppl. Methods).

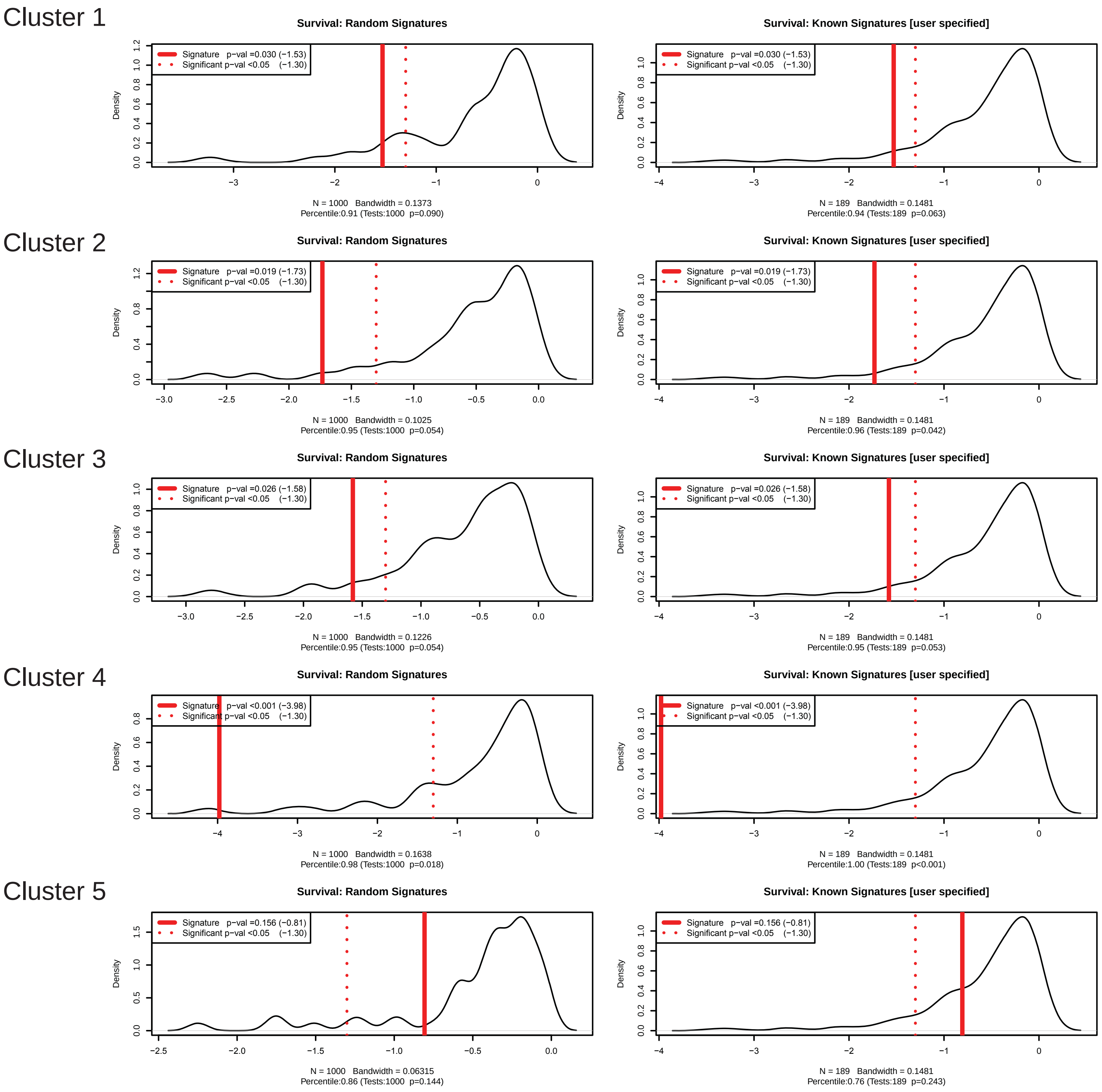

Supplementary Figure 17. Cluster-specific refinement of 100-gene set improves prediction of relapse. Subsets of the 100-gene set were identified that best describes each iCluster (see Suppl. Table 11 for gene lists, and Suppl. Methods). These were tested for power to predict relapse in the Stockholm validation set, against 1 000 random signatures of similar size (left column; p<0.001), and 189 oncological signatures (right colum; p<0.001) (Subramanian et al., 2005). In each case, the solid red line shows the performance of each Cluster-specific gene signature, as compared to dotted red line p=0.05, against the background/null distribution of the gene signatures tested.

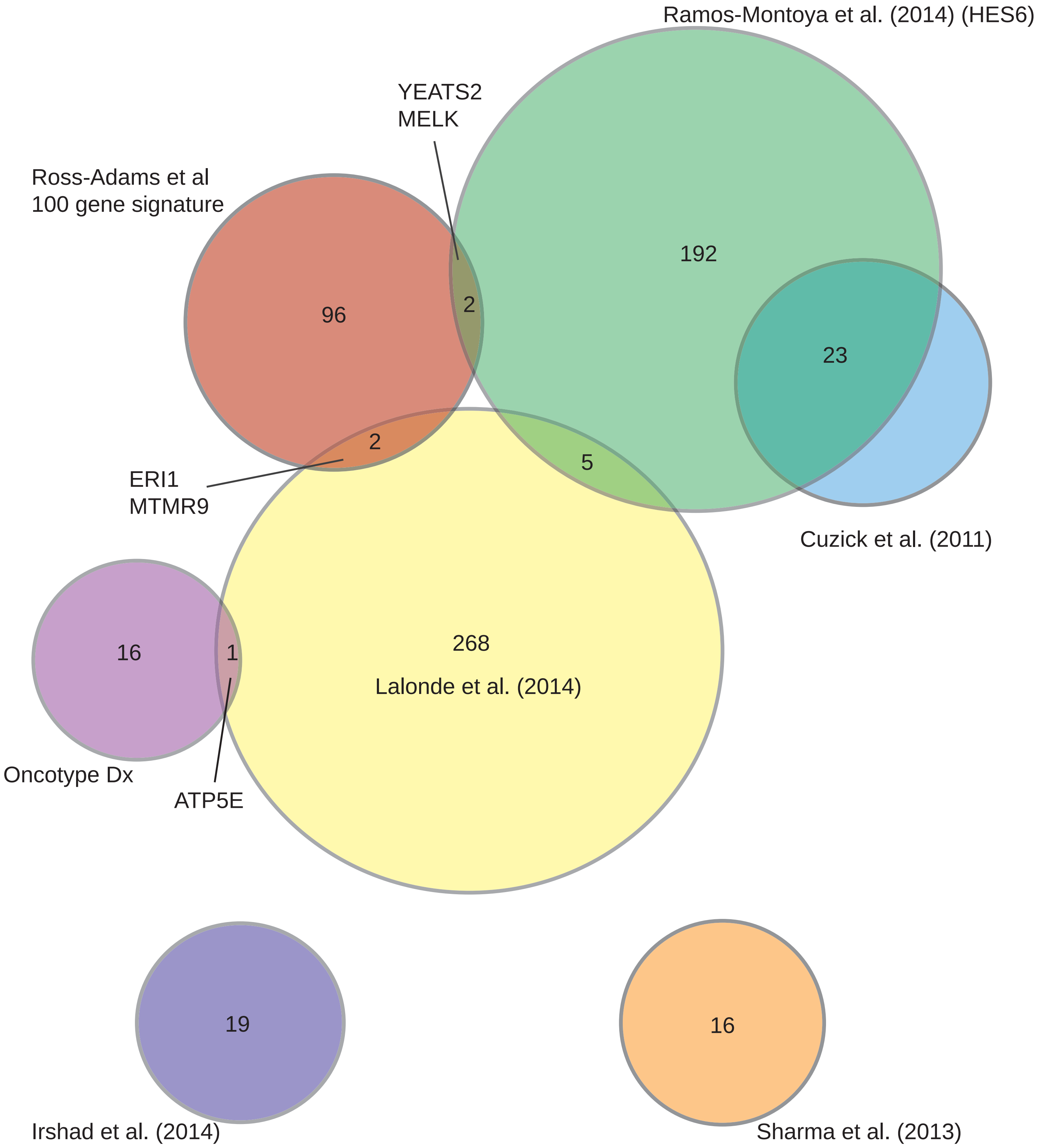

Supplementary Figure 18. **Published gene signature overlap.**
Intersects compared between our 100 gene-set and other published gene signatures as shown.

SUPPLEMENTARY METHODS

Integrative clustering

The GenomicRanges and GenomicFeatures infrastructure in Bioconductor was used to define a set of gene-centric coordinates for hg19, and to perform overlaps between the copy number and expression data.[1] For each gene in the TxDb.Hsapiens.UCSC.hg19.knownGene database, we computed a single copy-number and expression value for each sample. For the Cambridge cohort, copy number values were taken as the OncoSNP[2] Rank 1 call with most-aberrant log-ratio that overlapped the gene. For Stockholm, the longest segment overlapping the gene was used in order to remove short, spurious calls from the data. In both cohorts, gene-centric expression data were derived by first considering Illumina expression probes that overlapped the coordinates of each gene. In the case of multiple overlapping probes, the probe with the highest inter-quartile range (IQR) was used. An ANOVA test was used to compute a p-value, testing the association between expression and copy-number for each gene. All genes were then ranked according to this p-value; lowest (i.e. strongest evidence for association) to highest. The top N ranked genes where then taken forward for integrative analysis; where increasing values of N were used in order to determine to optimal number of features (see below).

Integrative analysis (cluster identification) was performed using copy number and gene expression data from the same 125 PC tissue samples in iCluster+[3], with seed set to zero to ensure that the same parameters were generated each time where iCluster+ uses kNN (nearest neighbour) method. All features were included, and used to generate diagnostic plots in both discovery and validation cohorts. To increase stability, penalty parameters (lambda) were set to zero for all (no penalty). Variable selection was done after cluster generation, to reduce variability in results. Since the percentage variation explained naturally increases with an increase in number of clusters (and a reduction in cluster size), we selected the optimal *k* (5 clusters=*k*+1; *k*=4) at the point beyond which additional sample divisions provided no significant improvement in variation explained – see Suppl. Fig 5. In-group proportions (IGP) were used to determine optimal number of *k*=4 (5 clusters), where IGP is defined as the proportion of data in a group whose nearest neighbours are classified to the same group (Pearson's centred correlation).

The stability of iCluster+ was confirmed by considering the *distribution* of explained variation for each *k* after running 100 different analyses with 100 features each and a range of *k*=1 to 10. There was no value for *k* after which the explained variation became more unstable; clusters were reliably reproduced. After training of the classifier using primary tumours of the Cambridge cohort with iCluster+[3],pamr[4] in Bioconductor was used to predict clusters for the Cambridge CRPC samples, as well as in the Stockholm and Taylor et al., (2010)[5] validation cohorts.

iCluster comparison

A core set of 106 samples pairs were used to accurately compare partitioning of samples based on either CN or mRNA or CN+mRNA data (integrative). We specified the iClusters to be ‘true’, and applied the recommended Adjusted Rand Index (ARI)[6]using fpc in Bioconductor to measure the relative accuracy of alternative clustering, where identical clustering gives ARI=1, and ARI=0 implies completely random re-clustering. Since there is no absolute threshold for similarity/divergence (but 0·35 would not occur by chance; Suppl. Table 6), and outputs must be considered relative to the set ‘true’ value, we also calculated the Variation of Information Index (VII)[7] in Bioconductor, where VII=0 for identical clustering and VII>0 for more disparate groupings; there is no maximum value.

Cambridge prostate cancer (CamCaP) gene signature analysis

This section describes an analysis of the genes and features identified as driving our clustering, treated as a prognostic gene signature. The goal is to identify the ability of these genes to stratify patients into outcome groups, and to determine how unique they are in predicting outcome for prostate cancer patients.

Genes, features, training set
We identified 100 genes for use in deriving tumour subtype clusters. For every sample, each of these genes has two features: a gene expression value and a copy number value. The genes were chosen based on a correlation between the expression value and the copy number values in the Cambridge (discovery) cohort samples (i.e. eQTL), here known as the *training/discovery set*.

Validation Sets
Evaluation of the performance of the 100-gene set was performed using the Stockholm patient set, consisting of 103 samples, 99 with both copy number and expression and of which 85 samples had relapse information.

Patient categories
Patients were divided into two categories using the 100-gene set by calculating the mean expression over all the features associated with genes in the signature for each sample, and selecting a "high" enriched group consisting of those above the 33rd percentile, and a "low" enriched group at or below the 33rd percentile.

Prognostic power of the gene signature
The baseline test of the gene signature involved survival analyses on the validation data to determine the ability of the gene signature to separate a class of patients with worse outcome (as measured by time to relapse) from other, better performing patients. For this, we generated Kaplan-Meier plots, and computed associated p-values using a chi-squared test. For this test, the p-value was computed as p=0·017 (Suppl. Fig 14A).

Specificity of the gene signature
It has been shown that most oncogenic gene signatures are highly predictive of prognosis in breast cancer, even if they were derived using data for a different cancer[8]. We therefore performed a more rigorous set of tests to determine whether our gene signature had prognostic power beyond other possible gene sets. For this we used SigCheck in Bioconductor[9]. This package performs tests again randomly selected gene signatures, as well as sets of previously published signatures with discriminatory power value in various oncogenic contexts.

a. **Compared to random sets of genes**
We tested the p-value of our signature against a null distribution of random signatures of similar size (number of genes). This distribution is constructed by generating 1 000 random samples of 1 000 genes, and repeating the survival analysis for each set. An empirical p-value was computed by counting the number of random gene signatures with p-values equal to or lower than that computed to our gene signature. Our gene signature performed at the 97th percentile, for an empirical p-value computed at p=0·033. Suppl. Fig 14B shows how the signature compares to the background distribution of randomly selected 100-gene signatures.

b. Compared to known gene signatures

The second set of tests is against previously published gene signatures. We use two sets of cancer signatures.

1. A set of 189 oncogenic signatures in MSigDB[10].
   Our signature performed at the 96th percentile, for an empirical p-value of p=0·042.
2. A set of six signatures specific to prostate cancer:
   - Sharma (2013): 16 genes relating to CRPC[11]
   - Cuzick (2011): 31 genes relating to prognosis in primary PC[12]
   - Irshad (2013): 19 genes relating to indolent PC[13]
   - HES6 (2014): A set of 222 genes associated with HES6 & CRPC[14]
   - Lalonde (2014): A set of 276 genes (corresponding to 100 loci) relating to chromosome instability, and predicting <5 year relapse (intended to be used with copy number data only)[15]
   - OncoTypeDx: A set of 17 genes on commercial Oncotype Dx prognostic arrays

Not only did our signature outperform all six of these, none of the previously identified prostate-cancer specific signatures achieved significant results on the Stockholm dataset, mean gene expression:

Signature	p-value
SHARMA	0·174
CUZICK	0·263
IRSHAD	0·853
HES6	0·189
LALONDE	0·495
ONCOTYPEDX	0·732

Cluster-specific signatures

In our final analysis of signature performance, we looked at the features *within* our 100 gene set that were most important for defining our five clusters, yielding five distinct prognostic signatures (one for each cluster). We wanted to see if any of these five clusters had particularly interesting prognostic power.

We obtained a signature for each cluster by ranking the importance of features on a classification task, separating the samples in that cluster from those in other clusters. This was repeated for each cluster. As we had 100 genes and two features for each gene (expression and copy number), we had a total of 200 features from which to select a signature. For this analysis, we generated signatures comprised of the genes corresponding to the 50 highest-ranked *features* for each cluster, selected using rankings computed by the PAMR package in Bioconductor[4]. These signatures were then each applied to the Stockholm validation set (with no *a priori* clustering) (Suppl. Fig 16). When both the copy number and expression features for the same gene were used, these mapped to only one gene in the signature, so there could be as few as 25 genes in a signature.

Four out of five of the clusters exhibited significant ability to separate a poor outcome class in the Stockholm validation set, as shown in Suppl. Fig 15 and Table 3. In addition, it showed how none of the five previously identified signatures or the commercially available OncoType Dx gene signature, was able to separate a distinct survival group using mean expression values. We next compared the performance of the cluster signatures to 1 000

signatures of equivalent size (number of genes), as well as 189 previously identified oncogenic signatures (see Suppl. Fig 16).

The best performing cluster signature is that obtained for iCluster4 (purple curve; figure 3). On the survival task, we calculated a p-value of 0·0001. When compared to random signatures, iCluster4 signature performed in the 98th percentile for an empirical p-value of p=0·018. This signature also out-performed all 189 oncogenic signatures from MSigDB (Suppl. Fig 16 & Suppl. Table 9). These are presented in PAMR-ranked order of importance in discriminating the cluster. Further investigation showed that not all 50 genes are required to generate good survival performance in the Stockholm validation set, with even lower p-values obtained for as few as 15-20 genes. However, we strictly applied the same criteria for all five signatures in the discovery cohort, and did not select a lower cut-off that performed better in the validation set, since performance in a validation set cannot be used to set any parameters.

Supplementary Methods - References

1. Lawrence M, Huber W, Pagès H, et al. Software for Computing and Annotating Genomic Ranges. *PLoS Comput Biol* 2013; **9**(8): e1003118.
2. Yau C, Mouradov D, Jorissen RN, et al. A statistical approach for detecting genomic aberrations in heterogeneous tumor samples from single nucleotide polymorphism genotyping data. *Genome biology* 2010; **11**(9): R92.
3. Mo Q, Wang S, Seshan VE, et al. Pattern discovery and cancer gene identification in integrated cancer genomic data. *Proceedings of the National Academy of Sciences of the United States of America* 2013; **110**(11): 4245-50.
4. Tibshirani R, Hastie T, Narasimhan B, Chu G. Class Prediction by Nearest Shrunken Centroids, with Applications to DNA Microarrays. 2003: 104-17.
5. Taylor BS, Schultz N, Hieronymus H, et al. Integrative genomic profiling of human prostate cancer. *Cancer cell* 2010; **18**(1): 11-22.
6. Hubert L, Arabie P. Comparing partitions. *Journal of Classification* 1985; **2**(1): 193-218.
7. Meilă M. Comparing clusterings—an information based distance. *Journal of Multivariate Analysis* 2007; **98**(5): 873-95.
8. Venet D, Dumont JE, Detours V. Most Random Gene Expression Signatures Are Significantly Associated with Breast Cancer Outcome. *PLoS Comput Biol* 2011; **7**(10): e1002240.
9. Norden JS, Rory. SigCheck: Check a gene signature's classification performance against random signatures, permuted data, and known signatures. R package version 1.0.2. ed; 2014.
10. Subramanian A, Tamayo P, Mootha VK, et al. Gene set enrichment analysis: A knowledge-based approach for interpreting genome-wide expression profiles. *Proceedings of the National Academy of Sciences* 2005; **102**(43): 15545-50.
11. Sharma NL, Massie CE, Ramos-Montoya A, et al. The androgen receptor induces a distinct transcriptional program in castration-resistant prostate cancer in man. *Cancer cell* 2013; **23**(1): 35-47.
12. Cuzick J, Swanson GP, Fisher G, et al. Prognostic value of an RNA expression signature derived from cell cycle proliferation genes in patients with prostate cancer: a retrospective study. *The Lancet Oncology* 2011; **12**(3): 245-55.
13. Irshad S, Bansal M, Castillo-Martin M, et al. A molecular signature predictive of indolent prostate cancer. *Science translational medicine* 2013; **5**(202): 202ra122.
14. Ramos-Montoya A, Lamb AD, Russell R, et al. HES6 drives a critical AR transcriptional programme to induce castration-resistant prostate cancer through activation of an E2F1-mediated cell cycle network. *EMBO Mol Med* 2014; **6**(5): 651-61.
15. Lalonde E, Ishkanian AS, Sykes J, et al. Tumour genomic and microenvironmental heterogeneity for integrated prediction of 5-year biochemical recurrence of prostate cancer: a retrospective cohort study. *The Lancet Oncology* 2014; **15**(13): 1521-32.

CASE EXAMPLE

Integration of copy number and transcriptomics provides risk stratification in prostate cancer: a discovery and validation cohort study

H Ross-Adams[1]*, AD Lamb[1,2,3]*, MJ Dunning[1*], S Halim[1], J Lindberg[4], CM Massie[1], LA Egevad[5], R Russell[1], A Ramos-Montoya[1], S Vowler[1], NL Sharma[6], J Kay[1,7], H Whitaker[1,7], J Clark[8], R Hurst[8], VJ Gnanapragasam[2,3], NC Shah[2], AY Warren[9], CS Cooper[8], AG Lynch[1], R Stark[1], IG Mills[1,10,11,12], H Grönberg [3]†, DE Neal [1,2]† on behalf of the CamCaP Study Group‡

Men who have clinically low risk disease at diagnosis but who turn out to have unexpectedly aggressive disease are a problem for the prostate cancer medical community. There seem to be two principal reasons for this. The first is sampling error. Prostate biopsies cannot sample the whole prostate gland with complete accuracy and therefore may miss the dominant tumour nodule. But the second is that our current methods in disease risk stratification are inadequate. In our study we have proposed a molecular classification for men with prostate cancer that will improve such stratification.

We present here a case where such molecular knowledge, derived from information discovered in our study, would have changed clinical management.

<u>Case</u>

This man was diagnosed with prostate cancer in 2008 and his key clinical parameters are outlined below.

	Diagnostic Biopsy	Radical Prostatectomy
Gleason Score	3+3=6; 3/10 cores; 5%	3+4=7
PSA	4.3	No change
TNM	T1cN0Mx	T2aN0Mx
ECE	N/A	No
SMS	N/A	Negative

On the basis of these details he would be considered to have low risk prostate cancer according to D'Amico criteria at biopsy. He was upgraded and upstaged at prostatectomy but would still have been considered to be at relatively low risk of relapse (see Figure 1). If the Memorial Sloane Kettering nomogram is used (https://www.mskcc.org/nomograms/prostate/pre-op) then he would be considered to have a probability of progression-free survival at 5 years of 94% based on the diagnostic biopsy and 91% based on the prostatectomy specimen.

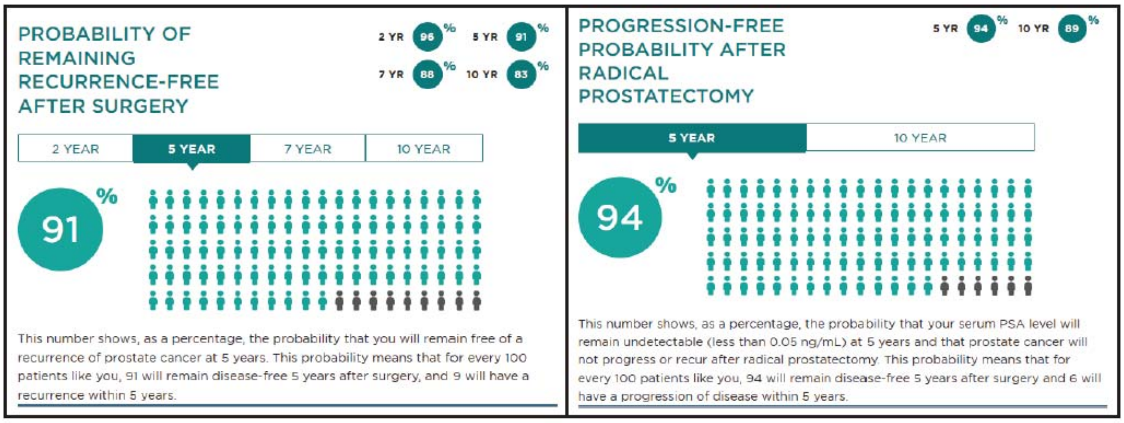

Figure 1. Memorial Sloane Kettering prostate cancer risk nomogram.
Patient details input to https://www.mskcc.org/nomograms/prostate for both pre-operative and post-operative outcomes.

On integrated genomic analysis, this man was found to be in iCluster3, our highest risk subgroup. So, although his clinical data suggested he was at low risk of relapse he is actually, according to our data, between 30 and 78% likely to relapse. In the event, this man had adjuvant / early salvage radiotherapy at 7 months and this seems to have been effective as his PSA has remained steady since (Figure 2) suggesting that his relapse was due to recurrence from unidentified locally advanced disease (local equivalent of micrometastatic disease). We propose that genomic information contained within this study could be used at the time of biopsy to advocate surgery with wider margins, or after prostatectomy to advocate early adjuvant radiotherapy or even neoadjuvant treatment with an appropriate small molecule.

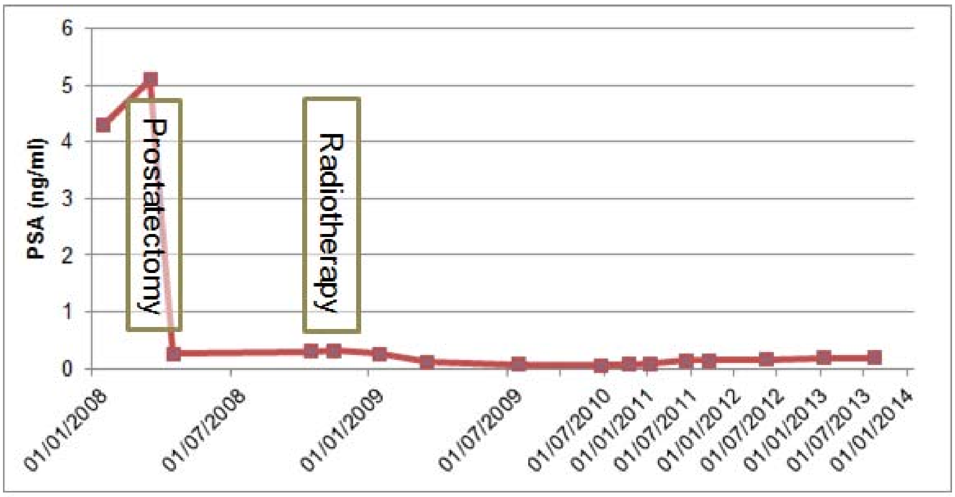

Figure 2. PSA kinetics from diagnosis to most recent recorded follow up.

In addition to signature profiling another area in which this information could be used is in immunohistochemistry of biopsy or prostatectomy specimens. In order to help investigate this we generated a tissue microarray (TMA) containing tumour cores for every man in the Cambridge cohort undergoing prostatectomy. These specimens were taken from regions of the prostate paraffin megablock directly adjacent to the frozen section from which nucleic acids were extracted. A representative H&E stain of one of the four TMA blocks is shown (Figure 3A) as well as of the man

described here. A core demonstrating his prostate cancer is presented (Figure 3B) as well as our first attempt at staining for a protein coded by a member of the 100-gene set presented in this study, MELK. This staining needs optimising and it is hoped that further antibody selection for genes discriminating the high-risk subgroups will yield clinically useful diagnostic information.

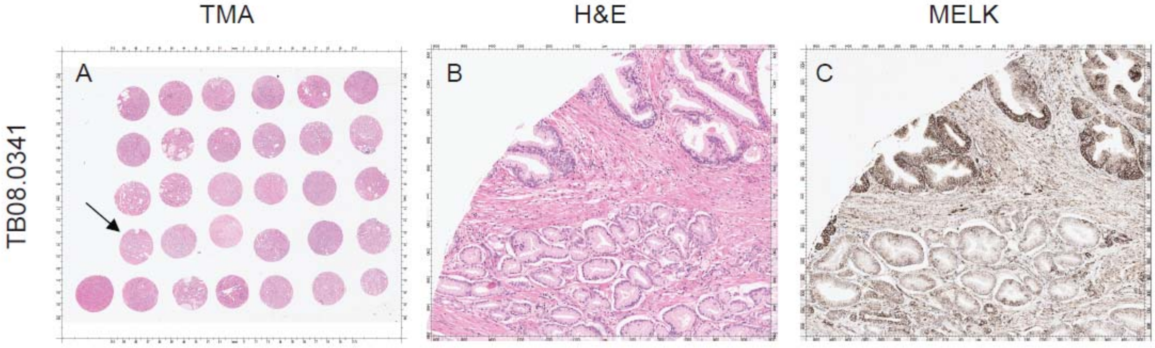

Figure 3. Histology from CamCaP robotic prostatectomy TMA
A. Screenshot of relevant block of TMA (one of four blocks). Each 3mm core is taken from a section of megablock neighbouring the region from which nucleic acids were extracted for integrated analysis. Arrow indicates the man in this case example.
B. Haematoxylin & Eosin stain at 10x magnification of region of infiltrating cancer. Scale as shown.
C. MELK immunohistochemistry (IHC, Sigma Atlas #HPA017214). MELK is one of the 100-gene set of discriminating genes. Staining is poor here. Further work-up of relevant antibodies is needed to identify members of the gene-set that are accurate in identifying members of high-risk subgroups before an IHC/histological approach could be used.

Supplementary Table 1. Available prostate cancer datasets with both mRNA and copy number data

**Dataset**	**Platform Gene expression**	**Platform CN**	**# primary tumours**	**# advanced tumours**
Varambally *et al.* (2005)	Affymetrix U133 2.0	N/A	7	6
Tomlins *et al.* (2005)	not reported	N/A	22	9
Taylor *et al.* (2010)	Affymetrix Human Exon 1.0 ST	Agilent 244K	**109**	**19**
Markert et al. (2011)	Illumina Custom Array (6,144 genes)	N/A	281	
Barbieri *et al.* (2012)	Illumina RNA-seq	Affymetrix SNP6	112 (CN)<br>22 (mRNA & CN)	-
Grasso *et al.* (2012)	Exome-seq (Illumina GAIIx)	Agilent 105K or 244K	59	32
Baca *et al.* (2013)*	Exome-seq (Illumina GAIIx)	WGS (Illumina GAIIx) & Affymetrix SNP 6	55	2
TCGA - provisional‡	Illumina RNA-seq	Affymetrix SNP6	171	-

* data not available
‡ data only available after analysis completed

Supplementary Table 3. Frequencies of copy number variations (CNVs) in known prostate cancer risk genes in discovery and validation cohorts

genes tagging region*	chr	% other primary PC cohorts*	% Cambridge discovery cohort	% Stockholm validation cohort
NKX3-1	8p	55.7%	39.6%	44.7%
RB1	13q	44.9%	23.6%	42.7%
RYBP	3p13	19.8%	19.8%	32.0%
PTEN	10q	26.4%	17.9%	37.9%
CDKN1B	12p	23.8%	17.0%	29.1%
SHQ1	3p13	19.8%	15.1%	30.1%
MAP3K7	6q	40.8%	15.1%	45.6%
FOXP1	3p13	19.8%	15.1%	29.1%
MYC	8q24	20.9%	13.2%	36.9%
TP53	17p	28.4%	11.3%	32.0%
BRCA2	13q	44.9%	9.4%	32.0%
BCL2	18q	25.5%	7.5%	35.9%
MCM7	7p	14.1%	7.5%	33.0%
CDH1	16q	44.3%	7.5%	31.1%
CHD1	5q	35.9%	6.6%	37.9%
APC	5q	35.9%	6.6%	36.9%
ETV4	17q	17.4%	6.6%	30.1%
ETV5	3q	9.7%	6.6%	29.1%
SMAD4	18q	25.5%	5.7%	35.9%
CXCR4	2q	22.9%	4.7%	37.9%
EGFR	7p	14.1%	4.7%	31.1%
PIK3CA	3q	9.7%	4.7%	30.1%
BRCA1	17q	17.4%	3.8%	27.2%
BRAF	7p	14.1%	3.8%	31.1%
ERG	21q	25.2%	2.8%	15.5%
ETV1	7p	14.1%	1.9%	32.0%
TMPRRS2	21q	25.1%	0.0%	19.4%

* From Williams et al. (2014) meta-analysis of recurrent CNVs in PC

Cambridge cohort OMNI2.5M data were analysed in OncoSNP (Yau et al., 2010); all rank calls 1-5 counted

Stockholm SNP6 data were analysed in ASCAT (van Loo et al., 2010); no filtering on calls

Supplementary Table 4. All genes with CN altered in ≥ 10% Cambridge samples tested (OMNI2.5M; Onc

chr	chrStart	chrEnd	gene	strand	CNA type
chr8	47752507	47767407	LINC00293	+	gain
chr8	48100929	48104439	LOC100287846	+	gain
chr8	48173488	48678652	SPIDR	+	gain
chr8	48872762	48890719	MCM4	+	gain
chr8	48920994	48974454	UBE2V2	+	gain
chr8	49966894	49988642	C8orf22	+	gain
chr8	50822348	51705427	SNTG1	+	gain
chr8	53852467	53853454	NPBWR1	+	gain
chr8	54764367	54871863	RGS20	+	gain
chr8	55047780	55061074	MRPL15	+	gain
chr8	55370494	55373456	SOX17	+	gain
chr8	55528626	55682531	RP1	+	gain
chr8	56015016	56438710	XKR4	+	gain
chr8	56685790	56738005	TGS1	+	gain
chr8	56792385	56925006	LYN	+	gain
chr8	57124314	57131176	CHCHD7	+	gain
chr8	58131647	58145388	LOC100507651	+	gain
chr8	58173784	58179170	LOC286177	+	gain
chr8	58192101	58197290	LINC00588	+	gain
chr8	58907112	59062277	FAM110B	+	gain
chr8	59323822	59364060	UBXN2B	+	gain
chr8	59465727	59495419	SDCBP	+	gain
chr8	61429468	61536203	RAB2A	+	gain
chr8	61591323	61780586	CHD7	+	gain
chr8	62200524	62414204	CLVS1	+	gain
chr8	62627346	62627418	MIR4470	+	gain
chr8	63161500	63903628	NKAIN3	+	gain
chr8	63890419	63897460	UG0898H09	+	gain
chr8	64081111	64125346	YTHDF3	+	gain
chr8	64681987	64698054	LINC01289	+	gain
chr8	65285774	65295842	MIR124-2HG	+	gain
chr8	65291705	65291814	MIR124-2	+	gain
chr8	65492794	65496191	BHLHE22	+	gain
chr8	66556887	66622798	MTFR1	+	gain
chr8	66933790	67012755	DNAJC5B	+	gain
chr8	67039277	67087718	TRIM55	+	gain
chr8	67104348	67109554	LINC00967	+	gain
chr8	67341262	67342968	RRS1	+	gain
chr8	67344717	67381044	ADHFE1	+	gain
chr8	67405490	67430759	C8orf46	+	gain
chr8	67579786	67593377	C8orf44	+	gain
chr8	67579786	67774257	C8orf44-SGK3	+	gain
chr8	67624652	67774257	SGK3	+	gain
chr8	67782983	67834283	MCMDC2	+	gain
chr8	67976602	68108849	CSPP1	+	gain
chr8	68864602	69143897	PREX2	+	gain
chr8	69242956	69731258	C8orf34	+	gain
chr8	70378858	70573147	SULF1	+	gain

chr8	71520811	71575513	LOC286190	+	gain
chr8	71581599	71648177	XKR9	+	gain
chr8	72755357	72968547	LOC100132891	+	gain
chr8	73449625	73850584	KCNB2	+	gain
chr8	73921096	73959987	TERF1	+	gain
chr8	74206836	74237520	RDH10	+	gain
chr8	74332308	74353753	STAU2-AS1	+	gain
chr8	74888376	74895018	TMEM70	+	gain
chr8	74903563	74941307	LY96	+	gain
chr8	75262617	75279335	GDAP1	+	gain
chr8	75460777	75460852	MIR5681A	+	gain
chr8	75512100	75670587	FLJ39080	+	gain
chr8	75617927	75617982	MIR2052	+	gain
chr8	75736771	75767264	PI15	+	gain
chr8	75896707	75946793	CRISPLD1	+	gain
chr8	76320182	76479061	HNF4G	+	gain
chr8	77593514	77779521	ZFHX4	+	gain
chr8	79428335	79517502	PKIA	+	gain
chr8	79578281	79631997	ZC2HC1A	+	gain
chr8	80523048	80578410	STMN2	+	gain
chr8	81153623	81153708	MIR5708	+	gain
chr8	81397853	81438500	ZBTB10	+	gain
chr8	82192717	82197012	FABP5	+	gain
chr8	82644687	82671748	CHMP4C	+	gain
chr8	85095452	85834078	RALYL	+	gain
chr8	86019322	86058314	LRRCC1	+	gain
chr8	86089618	86126753	E2F5	+	gain
chr8	86132844	86352138	CA13	+	gain
chr8	86351055	86361267	CA3	+	gain
chr8	86376130	86393721	CA2	+	gain
chr8	87111138	87166454	ATP6V0D2	+	gain
chr8	87354993	87480178	WWP1	+	gain
chr8	87526655	87573726	CPNE3	+	gain
chr8	87878675	88394955	CNBD1	+	gain
chr8	90769974	90803292	RIPK2	+	gain
chr8	90914095	90940095	OSGIN2	+	gain
chr8	91013579	91064227	DECR1	+	gain
chr8	91233715	91400187	LINC00534	+	gain
chr8	91803920	91971630	NECAB1	+	gain
chr8	92082423	92099323	OTUD6B	+	gain
chr8	92114846	92231464	LRRC69	+	gain
chr8	92217712	92217786	MIR4661	+	gain
chr8	92221721	92410378	SLC26A7	+	gain
chr8	94712772	94740671	FAM92A1	+	gain
chr8	94752338	94753047	RBM12B-AS1	+	gain
chr8	94767071	94830347	TMEM67	+	gain
chr8	94929082	94938296	PDP1	+	gain
chr8	95653363	95719694	ESRP1	+	gain
chr8	95732102	95806076	DPY19L4	+	gain
chr8	95835517	95892721	INTS8	+	gain

chr8	95970235	96088396	NDUFAF6	+	gain
chr8	96085141	96085221	MIR3150A	+	gain
chr8	96145948	96168913	PLEKHF2	+	gain
chr8	96281063	96822371	C8orf37-AS1	+	gain
chr8	97274166	97346774	PTDSS1	+	gain
chr8	97505881	97624037	SDC2	+	gain
chr8	97657498	98155722	CPQ	+	gain
chr8	98656406	98742488	MTDH	+	gain
chr8	98787808	98864830	LAPTM4B	+	gain
chr8	98881310	99048946	MATN2	+	gain
chr8	99076749	99105838	ERICH5	+	gain
chr8	99129520	99172069	POP1	+	gain
chr8	99439249	99443023	KCNS2	+	gain
chr8	99956630	99964326	OSR2	+	gain
chr8	100025493	100889814	VPS13B	+	gain
chr8	101162838	101166230	POLR2K	+	gain
chr8	101170262	101254132	SPAG1	+	gain
chr8	101394990	101395073	MIR4471	+	gain
chr8	102064281	102088479	FLJ42969	+	gain
chr8	102381120	102381823	NACAP1	+	gain
chr8	102504667	102681952	GRHL2	+	gain
chr8	103137659	103137743	MIR5680	+	gain
chr8	103563847	103573245	ODF1	+	gain
chr8	104033247	104085285	ATP6V1C1	+	gain
chr8	104152920	104242533	BAALC	+	gain
chr8	104166841	104166917	MIR3151	+	gain
chr8	104310660	104345094	FZD6	+	gain
chr8	104383742	104395232	CTHRC1	+	gain
chr8	104426941	104455680	DCAF13	+	gain
chr8	104512975	105266656	RIMS2	+	gain
chr8	105352023	105368917	DCSTAMP	+	gain
chr8	106331146	106816767	ZFPM2	+	gain
chr8	107282405	107764921	OXR1	+	gain
chr8	109455852	109499136	EMC2	+	gain
chr8	110099652	110131812	TRHR	+	gain
chr8	110346551	110358189	ENY2	+	gain
chr8	110374705	110543500	PKHD1L1	+	gain
chr8	110551928	110577391	EBAG9	+	gain
chr8	113655721	113655812	MIR2053	+	gain
chr8	117778741	117786921	UTP23	+	gain
chr8	117886662	117889107	RAD21-AS1	+	gain
chr8	117950463	117956239	AARD	+	gain
chr8	117962511	118188953	SLC30A8	+	gain
chr8	118532964	118552501	MED30	+	gain
chr8	119633239	119738306	SAMD12-AS1	+	gain
chr8	120079423	120119202	COLEC10	+	gain
chr8	120220609	120257914	MAL2	+	gain
chr8	120428551	120436678	NOV	+	gain
chr8	120885899	121063157	DEPTOR	+	gain
chr8	121137346	121384273	COL14A1	+	gain

chr8	121457637	121535875	MTBP	+	gain
chr8	122651585	122657564	HAS2-AS1	+	gain
chr8	123793900	123986755	ZHX2	+	gain
chr8	124084919	124164392	TBC1D31	+	gain
chr8	124191286	124222318	FAM83A	+	gain
chr8	124428964	124454260	WDYHV1	+	gain
chr8	124780881	124827690	FAM91A1	+	gain
chr8	124864226	125132302	FER1L6	+	gain
chr8	125463047	125465266	TRMT12	+	gain
chr8	125487007	125500859	RNF139	+	gain
chr8	125551342	125580751	NDUFB9	+	gain
chr8	125951883	125963337	LINC00964	+	gain
chr8	125985538	125991630	ZNF572	+	gain
chr8	126010719	126034525	SQLE	+	gain
chr8	126104082	126379367	NSMCE2	+	gain
chr8	126442562	126450644	TRIB1	+	gain
chr8	128025398	128033259	PCAT1	+	gain
chr8	128427856	128429441	POU5F1B	+	gain
chr8	128747764	128753680	MYC	+	gain
chr8	128806802	129113499	PVT1	+	gain
chr8	128808207	128808274	MIR1204	+	gain
chr8	128972878	128972941	MIR1205	+	gain
chr8	129021143	129021202	MIR1206	+	gain
chr8	129061397	129061484	MIR1207	+	gain
chr8	129162361	129162434	MIR1208	+	gain
chr8	132916355	133025886	EFR3A	+	gain
chr8	133787603	133861052	PHF20L1	+	gain
chr8	133879204	134147143	TG	+	gain
chr8	134203281	134243932	WISP1	+	gain
chr8	135610313	135612932	ZFAT-AS1	+	gain
chr8	136246373	136311962	LOC286094	+	gain
chr8	136469715	136659848	KHDRBS3	+	gain
chr8	141521396	141527252	CHRAC1	+	gain
chr8	142138719	142205906	DENND3	+	gain
chr8	142350647	142354720	LINC01300	+	gain
chr8	142432006	142441620	PTP4A3	+	gain
chr8	143257699	143257779	MIR4472-1	+	gain
chr8	143279716	143290364	LINC00051	+	gain
chr8	143545376	143626368	BAI1	+	gain
chr8	143751725	143764145	PSCA	+	gain
chr8	143781528	143785584	LY6K	+	gain
chr8	143808620	143818350	THEM6	+	gain
chr8	143916216	143928262	GML	+	gain
chr8	144077244	144079080	CDC42P3	+	gain
chr8	144099901	144103827	LY6E	+	gain
chr8	144120678	144135720	C8orf31	+	gain
chr8	144295067	144299044	GPIHBP1	+	gain
chr8	144328990	144359101	ZFP41	+	gain
chr8	144349606	144359101	GLI4	+	gain
chr8	144373558	144382120	ZNF696	+	gain

chr8	144451024	144466390	RHPN1	+	gain
chr8	144635556	144645231	GSDMD	+	gain
chr8	144680073	144682485	TIGD5	+	gain
chr8	144718182	144735900	ZNF623	+	gain
chr8	144766621	144794977	ZNF707	+	gain
chr8	144779284	144780583	BREA2	+	gain
chr8	144798506	144804633	MAPK15	+	gain
chr8	144816309	144828507	FAM83H-AS1	+	gain
chr8	145064225	145067583	GRINA	+	gain
chr8	145086581	145102015	SPATC1	+	gain
chr8	145133521	145135551	EXOSC4	+	gain
chr8	145137523	145141119	GPAA1	+	gain
chr8	145149941	145152428	CYC1	+	gain
chr8	145159304	145162515	MAF1	+	gain
chr8	145162628	145173218	KIAA1875	+	gain
chr8	145192671	145440828	HGH1	+	gain
chr8	145202918	145316843	MROH1	+	gain
chr8	145321516	145492131	SCX	+	gain
chr8	145515269	145538385	HSF1	+	gain
chr8	145582216	145584948	SLC52A2	+	gain
chr8	145597730	145618453	ADCK5	+	gain
chr8	145691737	145699499	KIFC2	+	gain
chr8	145715416	145727504	PPP1R16A	+	gain
chr8	145729464	145732555	GPT	+	gain
chr8	145734551	145736589	MFSD3	+	gain
chr8	145743348	145750559	LRRC14	+	gain
chr8	146024260	146036554	ZNF517	+	gain
chr8	146052902	146072894	ZNF7	+	gain
chr8	146220250	146224283	TMED10P1	+	gain
chr8	146228196	146231432	ZNF252P-AS1	+	gain
chr8	146277823	146281416	C8orf33	+	gain
chr8	48649475	48650726	CEBPD	-	gain
chr8	48685668	48872743	PRKDC	-	gain
chr8	49623350	49647870	EFCAB1	-	gain
chr8	49830238	49833999	SNAI2	-	gain
chr8	52232136	52722005	PXDNL	-	gain
chr8	52730139	52811735	PCMTD1	-	gain
chr8	53023391	53322439	ST18	-	gain
chr8	53446596	53478021	FAM150A	-	gain
chr8	53535017	53627026	RB1CC1	-	gain
chr8	54138275	54164194	OPRK1	-	gain
chr8	54628102	54755871	ATP6V1H	-	gain
chr8	54879115	54935008	TCEA1	-	gain
chr8	54958937	55014577	LYPLA1	-	gain
chr8	56361756	56367881	SBF1P1	-	gain
chr8	56651319	56685885	TMEM68	-	gain
chr8	56980738	56987140	RPS20	-	gain
chr8	56986397	56986460	SNORD54	-	gain
chr8	57025500	57026541	MOS	-	gain
chr8	57073467	57123859	PLAG1	-	gain

chr8	57212569	57233241	SDR16C5	-	gain
chr8	57353512	57359282	PENK	-	gain
chr8	57430877	57472382	LINC00968	-	gain
chr8	57870487	57906430	IMPAD1	-	gain
chr8	59402736	59412720	CYP7A1	-	gain
chr8	59496063	59572404	NSMAF	-	gain
chr8	59717976	60031767	TOX	-	gain
chr8	61101422	61193954	CA8	-	gain
chr8	61878679	61880307	LOC100130298	-	gain
chr8	62413114	62627199	ASPH	-	gain
chr8	63927638	63951610	GGH	-	gain
chr8	63972047	63998612	TTPA	-	gain
chr8	64080283	64081001	YTHDF3-AS1	-	gain
chr8	65486865	65494445	LOC401463	-	gain
chr8	65508528	65711348	CYP7B1	-	gain
chr8	66073379	66092575	LINC00251	-	gain
chr8	66439242	66474901	LINC01299	-	gain
chr8	66514690	66546452	ARMC1	-	gain
chr8	66626568	66753969	PDE7A	-	gain
chr8	67088611	67090846	CRH	-	gain
chr8	67331821	67341212	RRS1-AS1	-	gain
chr8	67474409	67525480	MYBL1	-	gain
chr8	67542487	67579452	VCPIP1	-	gain
chr8	67679631	67680240	PTTG3P	-	gain
chr8	67834164	67837777	SNHG6	-	gain
chr8	67834708	67834784	SNORD87	-	gain
chr8	67858735	67874825	TCF24	-	gain
chr8	67900366	67940786	PPP1R42	-	gain
chr8	67955314	67974571	COPS5	-	gain
chr8	68085746	68255912	ARFGEF1	-	gain
chr8	68334404	68658620	CPA6	-	gain
chr8	69215702	69243726	LOC286189	-	gain
chr8	69824037	70016425	LOC100505718	-	gain
chr8	70584567	70747299	SLCO5A1	-	gain
chr8	70963885	70983562	PRDM14	-	gain
chr8	71024266	71316020	NCOA2	-	gain
chr8	71485452	71520694	TRAM1	-	gain
chr8	71549500	71581447	LACTB2	-	gain
chr8	72109667	72274467	EYA1	-	gain
chr8	72753776	72756731	MSC	-	gain
chr8	72933485	72987819	TRPA1	-	gain
chr8	73117533	73163869	LOC392232	-	gain
chr8	73976777	74005507	SBSPON	-	gain
chr8	74153658	74171737	LOC100130301	-	gain
chr8	74202873	74205869	RPL7	-	gain
chr8	74332603	74659943	STAU2	-	gain
chr8	74692331	74791145	UBE2W	-	gain
chr8	74857372	74884522	TCEB1	-	gain
chr8	75146938	75233562	JPH1	-	gain
chr8	75460784	75460844	MIR5681B	-	gain

chr8	77523113	77595510	ZFHX4-AS1	-	gain
chr8	77892493	77913280	PEX2	-	gain
chr8	79635082	79636609	LOC101241902	-	gain
chr8	79645006	79717758	IL7	-	gain
chr8	80676244	80680098	HEY1	-	gain
chr8	80831094	80942506	MRPS28	-	gain
chr8	80831094	81083836	TPD52	-	gain
chr8	81540685	81787016	ZNF704	-	gain
chr8	81880045	82024303	PAG1	-	gain
chr8	82352563	82359719	PMP2	-	gain
chr8	82370617	82373758	FABP9	-	gain
chr8	82390731	82395473	FABP4	-	gain
chr8	82437280	82443550	FABP12	-	gain
chr8	82569150	82598589	IMPA1	-	gain
chr8	82605890	82607207	SLC10A5	-	gain
chr8	82613565	82633539	ZFAND1	-	gain
chr8	82711817	82754521	SNX16	-	gain
chr8	86126287	86132643	C8orf59	-	gain
chr8	86240457	86290342	CA1	-	gain
chr8	86566827	86757761	REXO1L2P	-	gain
chr8	86568694	86840171	REXO1L1P	-	gain
chr8	87060690	87081851	PSKH2	-	gain
chr8	87226287	87242609	SLC7A13	-	gain
chr8	87484577	87521009	RMDN1	-	gain
chr8	87586162	87755903	CNGB3	-	gain
chr8	88882970	88886296	DCAF4L2	-	gain
chr8	89049459	89339717	MMP16	-	gain
chr8	90945563	90996899	NBN	-	gain
chr8	91070837	91107687	CALB1	-	gain
chr8	91634222	91803859	TMEM64	-	gain
chr8	91970705	91997485	C8orf88	-	gain
chr8	92006501	92053203	TMEM55A	-	gain
chr8	92967194	93115454	RUNX1T1	-	gain
chr8	93725189	93798288	FLJ46284	-	gain
chr8	93895757	94029901	TRIQK	-	gain
chr8	94146323	94179079	C8orf87	-	gain
chr8	94358694	94712661	LINC00535	-	gain
chr8	94743730	94753224	RBM12B	-	gain
chr8	94928249	94928347	MIR378D2	-	gain
chr8	95139393	95229531	CDH17	-	gain
chr8	95261484	95274547	GEM	-	gain
chr8	95384187	95487343	RAD54B	-	gain
chr8	95439939	95449180	FSBP	-	gain
chr8	95500004	95565746	KIAA1429	-	gain
chr8	95649512	95651695	LOC100288748	-	gain
chr8	95892452	95907482	CCNE2	-	gain
chr8	95938199	95961615	TP53INP1	-	gain
chr8	96085138	96085224	MIR3150B	-	gain
chr8	96219234	96228602	LINC01298	-	gain
chr8	96257140	96281462	C8orf37	-	gain

chr8	96959212	96960576	LOC100500773	-	gain
chr8	97154557	97173020	GDF6	-	gain
chr8	97238903	97247862	UQCRB	-	gain
chr8	97251644	97273796	MTERF3	-	gain
chr8	98285713	98290176	TSPYL5	-	gain
chr8	99037078	99057818	RPL30	-	gain
chr8	99054313	99054445	SNORA72	-	gain
chr8	99114566	99129418	HRSP12	-	gain
chr8	99204386	99306621	NIPAL2	-	gain
chr8	99466858	99954799	STK3	-	gain
chr8	100548863	100548958	MIR599	-	gain
chr8	100549013	100549089	MIR875	-	gain
chr8	100890222	100906242	COX6C	-	gain
chr8	100973275	101118344	RGS22	-	gain
chr8	101145587	101158099	FBXO43	-	gain
chr8	101269287	101322379	RNF19A	-	gain
chr8	101521979	101572014	ANKRD46	-	gain
chr8	101585111	101661893	SNX31	-	gain
chr8	101715143	101734315	PABPC1	-	gain
chr8	101930803	101965623	YWHAZ	-	gain
chr8	102209265	102218292	ZNF706	-	gain
chr8	102698769	103137135	NCALD	-	gain
chr8	103216728	103251346	RRM2B	-	gain
chr8	103264501	103424917	UBR5	-	gain
chr8	103661004	103667983	KLF10	-	gain
chr8	103838535	103876397	AZIN1	-	gain
chr8	104145190	104153570	BAALCOS	-	gain
chr8	104177736	104298135	BAALC-AS1	-	gain
chr8	104410865	104427563	SLC25A32	-	gain
chr8	105391651	105479277	DPYS	-	gain
chr8	105501458	105601252	LRP12	-	gain
chr8	107771710	107782472	ABRA	-	gain
chr8	108261709	108510254	ANGPT1	-	gain
chr8	108911543	109095913	RSPO2	-	gain
chr8	109213971	109260959	EIF3E	-	gain
chr8	109619078	109799770	TMEM74	-	gain
chr8	110253147	110346350	NUDCD1	-	gain
chr8	110586404	110704020	SYBU	-	gain
chr8	110979232	110988076	KCNV1	-	gain
chr8	113235158	114449242	CSMD3	-	gain
chr8	116420723	116681228	TRPS1	-	gain
chr8	116962735	117337297	LINC00536	-	gain
chr8	117657054	117778494	EIF3H	-	gain
chr8	117858172	117887105	RAD21	-	gain
chr8	117886966	117887039	MIR3610	-	gain
chr8	118811601	119124058	EXT1	-	gain
chr8	119201694	119634234	SAMD12	-	gain
chr8	119935795	119964383	TNFRSF11B	-	gain
chr8	120569316	120651106	ENPP2	-	gain
chr8	120743013	120845074	TAF2	-	gain

chr8	120846180	120868170	DSCC1	-	gain
chr8	121408082	121457647	MRPL13	-	gain
chr8	121547984	121824309	SNTB1	-	gain
chr8	122625270	122653630	HAS2	-	gain
chr8	124025403	124054663	DERL1	-	gain
chr8	124213411	124214983	FAM83A-AS1	-	gain
chr8	124228027	124228103	MIR4663	-	gain
chr8	124232195	124253638	C8orf76	-	gain
chr8	124238428	124286727	ZHX1-C8orf76	-	gain
chr8	124260689	124287781	ZHX1	-	gain
chr8	124332090	124428590	ATAD2	-	gain
chr8	124510126	124553493	FBXO32	-	gain
chr8	124657914	124665190	KLHL38	-	gain
chr8	124693033	124749647	ANXA13	-	gain
chr8	124996377	125053023	FER1L6-AS1	-	gain
chr8	125323158	125384940	TMEM65	-	gain
chr8	125500734	125551329	TATDN1	-	gain
chr8	125563027	125740730	MTSS1	-	gain
chr8	126036502	126104061	KIAA0196	-	gain
chr8	126934766	126963441	LINC00861	-	gain
chr8	127564682	127570711	FAM84B	-	gain
chr8	128301928	128494384	CASC8	-	gain
chr8	130228712	130253486	LINC00977	-	gain
chr8	130760441	130799134	GSDMC	-	gain
chr8	130851838	131028897	FAM49B	-	gain
chr8	131020579	131020699	MIR5194	-	gain
chr8	131064350	131455906	ASAP1	-	gain
chr8	131094983	131097014	ASAP1-IT2	-	gain
chr8	131307600	131308779	ASAP1-IT1	-	gain
chr8	131792546	132052835	ADCY8	-	gain
chr8	133036466	133071627	OC90	-	gain
chr8	133073732	133117512	HHLA1	-	gain
chr8	133133104	133493004	KCNQ3	-	gain
chr8	133572744	133573726	HPYR1	-	gain
chr8	133584200	133687863	LRRC6	-	gain
chr8	133722191	133772914	TMEM71	-	gain
chr8	134048972	134115310	SLA	-	gain
chr8	134249413	134309547	NDRG1	-	gain
chr8	134467090	134584183	ST3GAL1	-	gain
chr8	135490030	135725292	ZFAT	-	gain
chr8	135812762	135812850	MIR30B	-	gain
chr8	135817118	135817188	MIR30D	-	gain
chr8	139142265	139509065	FAM135B	-	gain
chr8	139600477	139926236	COL22A1	-	gain
chr8	140613081	140715299	KCNK9	-	gain
chr8	140742585	141468678	TRAPPC9	-	gain
chr8	141541263	141645646	AGO2	-	gain
chr8	141668480	142011412	PTK2	-	gain
chr8	142217273	142264225	SLC45A4	-	gain
chr8	142366586	142377365	GPR20	-	gain

chr8	142443928	142517330	MROH5	-	gain
chr8	143293440	143484543	TSNARE1	-	gain
chr8	143692404	143695833	ARC	-	gain
chr8	143738873	143751401	JRK	-	gain
chr8	143783669	143808391	LOC100288181	-	gain
chr8	143822361	143823829	SLURP1	-	gain
chr8	143831627	143833952	LYPD2	-	gain
chr8	143845755	143859640	LYNX1	-	gain
chr8	143866297	143868008	LY6D	-	gain
chr8	143953772	143961236	CYP11B1	-	gain
chr8	143991974	143999259	CYP11B2	-	gain
chr8	144063447	144099807	LOC100133669	-	gain
chr8	144239330	144242053	LY6H	-	gain
chr8	144391496	144442147	TOP1MT	-	gain
chr8	144448792	144450805	RHPN1-AS1	-	gain
chr8	144510229	144512602	MAFA	-	gain
chr8	144519824	144623620	ZC3H3	-	gain
chr8	144648362	144654928	MROH6	-	gain
chr8	144656954	144660513	NAPRT	-	gain
chr8	144661866	144679845	EEF1D	-	gain
chr8	144686082	144691784	PYCRL	-	gain
chr8	144694787	144699732	TSTA3	-	gain
chr8	144788863	144790279	CCDC166	-	gain
chr8	144806102	144815914	FAM83H	-	gain
chr8	144815252	144815323	MIR4664	-	gain
chr8	144873089	144897549	SCRIB	-	gain
chr8	144895126	144895212	MIR937	-	gain
chr8	144898513	144911556	PUF60	-	gain
chr8	144915754	144924200	NRBP2	-	gain
chr8	144939911	144947434	EPPK1	-	gain
chr8	144989320	145050913	PLEC	-	gain
chr8	145019358	145019447	MIR661	-	gain
chr8	145051319	145060635	PARP10	-	gain
chr8	145106166	145115584	OPLAH	-	gain
chr8	145153535	145159140	SHARPIN	-	gain
chr8	145486055	145515120	BOP1	-	gain
chr8	145538246	145550567	DGAT1	-	gain
chr8	145554453	145559943	SCRT1	-	gain
chr8	145576885	145579269	TMEM249	-	gain
chr8	145579087	145583036	FBXL6	-	gain
chr8	145618445	145634733	CPSF1	-	gain
chr8	145619363	145619445	MIR939	-	gain
chr8	145625475	145625559	MIR1234	-	gain
chr8	145637797	145642273	SLC39A4	-	gain
chr8	145648999	145653927	VPS28	-	gain
chr8	145654162	145669812	TONSL	-	gain
chr8	145675314	145691031	CYHR1	-	gain
chr8	145699114	145701718	FOXH1	-	gain
chr8	145736666	145743210	RECQL4	-	gain
chr8	145747760	145752416	LRRC24	-	gain

chr8	145751602	145754458	C8orf82	-	gain
chr8	145754562	145911194	ARHGAP39	-	gain
chr8	145946293	145980970	ZNF251	-	gain
chr8	145998500	146012725	ZNF34	-	gain
chr8	146015153	146017805	RPL8	-	gain
chr8	146065828	146126846	COMMD5	-	gain
chr8	146102335	146126846	ZNF250	-	gain
chr8	146155743	146176274	ZNF16	-	loss
chr8	146198974	146228285	ZNF252P	-	loss
chr3	71803200	71804328	GPR27	+	loss
chr3	72200407	72223490	LINC00870	+	loss
chr3	71003864	71633140	FOXP1	-	loss
chr3	71591120	71591240	MIR1284	-	loss
chr3	71728439	71803924	EIF4E3	-	loss
chr3	71820805	71834357	PROK2	-	loss
chr3	72423743	72495774	RYBP	-	loss
chr3	72798427	72897598	SHQ1	-	loss
chr6	80816343	81055987	BCKDHB	+	loss
chr6	83072922	83077133	TPBG	+	loss
chr6	83777384	83881065	DOPEY1	+	loss
chr6	83903031	83906256	RWDD2A	+	loss
chr6	84222193	84235421	PRSS35	+	loss
chr6	84562984	84567234	RIPPLY2	+	loss
chr6	84569369	84670146	CYB5R4	+	loss
chr6	84743419	84800605	MRAP2	+	loss
chr6	86159301	86205509	NT5E	+	loss
chr6	87647023	87726397	HTR1E	+	loss
chr6	87862550	87973406	ZNF292	+	loss
chr6	88032305	88085428	SMIM8	+	loss
chr6	88054570	88075181	C6orf163	+	loss
chr6	88091719	88109459	C6orf164	+	loss
chr6	88117689	88205858	C6orf165	+	loss
chr6	88146443	88222057	SLC35A1	+	loss
chr6	88299784	88377172	ORC3	+	loss
chr6	88757506	88776550	SPACA1	+	loss
chr6	89790428	89794879	PNRC1	+	loss
chr6	89855768	89875288	PM20D2	+	loss
chr6	90142896	90343553	ANKRD6	+	loss
chr6	90539618	90584155	CASP8AP2	+	loss
chr6	90604187	90605819	GJA10	+	loss
chr6	91022460	91022552	MIR4464	+	loss
chr6	92231377	92231455	MIR4643	+	loss
chr6	94416800	94486199	TSG1	+	loss
chr6	96025372	96057328	MANEA	+	loss
chr6	96463844	96663488	FUT9	+	loss
chr6	96969701	97003151	UFL1	+	loss
chr6	97010423	97064512	FHL5	+	loss
chr6	97372495	97588630	KLHL32	+	loss
chr6	98472406	98472495	MIR2113	+	loss
chr6	99282579	99286666	POU3F2	+	loss

chr6	99968869	100033084	TSTD3	+	loss
chr6	100054649	100063454	PRDM13	+	loss
chr6	100441913	100524295	MCHR2-AS1	+	loss
chr6	101846668	102517958	GRIK2	+	loss
chr6	105404922	105531207	LIN28B	+	loss
chr6	105585561	105617819	BVES-AS1	+	loss
chr6	106534194	106557814	PRDM1	+	loss
chr6	106959304	107018334	AIM1	+	loss
chr6	107077440	107116292	QRSL1	+	loss
chr6	107349406	107372547	C6orf203	+	loss
chr6	107811316	107982513	SOBP	+	loss
chr6	108487214	108510013	NR2E1	+	loss
chr6	108616097	108844251	LACE1	+	loss
chr6	108881025	109005971	FOXO3	+	loss
chr6	109072856	109091145	LINC00222	+	loss
chr6	109169618	109295352	ARMC2	+	loss
chr6	109416355	109485115	CEP57L1	+	loss
chr6	109557816	109591717	LOC100996634	+	loss
chr6	109615505	109629423	CCDC162P	+	loss
chr6	109761930	109765122	SMPD2	+	loss
chr6	110012423	110146634	FIG4	+	loss
chr6	110300297	110301924	GPR6	+	loss
chr6	110501586	110553422	CDC40	+	loss
chr6	111135823	111216913	AMD1	+	loss
chr6	111279762	111289091	GTF3C6	+	loss
chr6	111303290	111346794	RPF2	+	loss
chr6	111408780	111544606	SLC16A10	+	loss
chr6	111580481	111590261	KIAA1919	+	loss
chr6	111804674	111923497	TRAF3IP2-AS1	+	loss
chr6	112375277	112390887	WISP3	+	loss
chr6	112408673	112423993	FAM229B	+	loss
chr6	112668531	112672498	RFPL4B	+	loss
chr6	114178526	114184652	MARCKS	+	loss
chr6	114225550	114242806	FLJ34503	+	loss
chr6	116359893	116361107	TPI1P3	+	loss
chr6	116421998	116566853	NT5DC1	+	loss
chr6	116575335	116759442	DSE	+	loss
chr6	116782532	116784934	FAM26F	+	loss
chr6	116832807	116839709	FAM26E	+	loss
chr6	116850175	116880031	FAM26D	+	loss
chr6	116892582	116914436	RWDD1	+	loss
chr6	116937641	116954148	RSPH4A	+	loss
chr6	117002366	117063030	KPNA5	+	loss
chr6	117198375	117253326	RFX6	+	loss
chr6	117586720	117594728	VGLL2	+	loss
chr6	117803819	117891020	DCBLD1	+	loss
chr6	117996616	118031886	NUS1	+	loss
chr6	118228688	118638839	SLC35F1	+	loss
chr6	118822535	118824996	BRD7P3	+	loss
chr6	118869441	118881587	PLN	+	loss

chr6	119103870	119104581	LOC100287632	+	loss
chr6	119215240	119230335	ASF1A	+	loss
chr6	82455446	82462428	FAM46A	-	loss
chr6	82879955	82957448	IBTK	-	loss
chr6	83602185	83775545	UBE3D	-	loss
chr6	83874592	83903655	PGM3	-	loss
chr6	83920109	84140938	ME1	-	loss
chr6	84262604	84419127	SNAP91	-	loss
chr6	84833959	84937335	CEP162	-	loss
chr6	85397078	85473954	TBX18	-	loss
chr6	86215214	86303629	SNX14	-	loss
chr6	86317501	86353043	SYNCRIP	-	loss
chr6	86386724	86388451	SNHG5	-	loss
chr6	86387011	86387086	SNORD50A	-	loss
chr6	86387306	86387377	SNORD50B	-	loss
chr6	87795215	87804865	CGA	-	loss
chr6	87992696	88038996	GJB7	-	loss
chr6	88224095	88299735	RARS2	-	loss
chr6	88384577	88411985	AKIRIN2	-	loss
chr6	88849584	88875767	CNR1	-	loss
chr6	89319988	89673348	RNGTT	-	loss
chr6	89805677	89827800	SRSF12	-	loss
chr6	89887222	89941007	GABRR1	-	loss
chr6	89966839	90025018	GABRR2	-	loss
chr6	90036343	90062619	UBE2J1	-	loss
chr6	90074334	90121995	RRAGD	-	loss
chr6	90277862	90348474	LYRM2	-	loss
chr6	90353230	90529442	MDN1	-	loss
chr6	90636246	91006627	BACH2	-	loss
chr6	91223291	91297020	MAP3K7	-	loss
chr6	93949739	94129300	EPHA7	-	loss
chr6	97241997	97285353	GPR63	-	loss
chr6	97337186	97345767	NDUFAF4	-	loss
chr6	97590036	97731061	MMS22L	-	loss
chr6	99321600	99395849	FBXL4	-	loss
chr6	99720792	99797531	FAXC	-	loss
chr6	99817347	99842082	COQ3	-	loss
chr6	99847840	99873207	PNISR	-	loss
chr6	99880183	99963308	USP45	-	loss
chr6	99990262	100016690	CCNC	-	loss
chr6	100367785	100442114	MCHR2	-	loss
chr6	100836749	100912805	SIM1	-	loss
chr6	100956607	101329224	ASCC3	-	loss
chr6	105175967	105307794	HACE1	-	loss
chr6	105384168	105388402	LINC00577	-	loss
chr6	105544698	105585049	BVES	-	loss
chr6	105605774	105627858	POPDC3	-	loss
chr6	105725441	105850999	PREP	-	loss
chr6	106632351	106773695	ATG5	-	loss
chr6	107018902	107078366	RTN4IP1	-	loss

chr6	107165326	107235300	LOC100422737	-	loss
chr6	107386384	107435636	BEND3	-	loss
chr6	107473760	107780779	PDSS2	-	loss
chr6	108023363	108145521	SCML4	-	loss
chr6	108188959	108279482	SEC63	-	loss
chr6	108362612	108395941	OSTM1	-	loss
chr6	108532716	108582464	SNX3	-	loss
chr6	109307639	109415708	SESN1	-	loss
chr6	109687716	109703762	CD164	-	loss
chr6	109711417	109762374	PPIL6	-	loss
chr6	109765265	109787171	MICAL1	-	loss
chr6	109783718	109804440	ZBTB24	-	loss
chr6	109814058	110012415	AK9	-	loss
chr6	110421021	110501207	WASF1	-	loss
chr6	110567148	110731939	METTL24	-	loss
chr6	110713382	110736753	DDO	-	loss
chr6	110745891	110797844	SLC22A16	-	loss
chr6	110931180	111136837	CDK19	-	loss
chr6	111367621	111368757	GSTM2P1	-	loss
chr6	111620233	111804918	REV3L	-	loss
chr6	111876580	111927477	TRAF3IP2	-	loss
chr6	111981534	112194655	FYN	-	loss
chr6	112391859	112408751	TUBE1	-	loss
chr6	112429133	112575917	LAMA4	-	loss
chr6	114189178	114194512	LINC01268	-	loss
chr6	114257319	114292359	HDAC2	-	loss
chr6	114376749	114663540	HS3ST5	-	loss
chr6	116262692	116381921	FRK	-	loss
chr6	116440084	116447296	COL10A1	-	loss
chr6	116571130	116575261	TSPYL4	-	loss
chr6	116596021	116601280	TSPYL1	-	loss
chr6	116817650	116866773	TRAPPC3L	-	loss
chr6	116956780	116989973	ZUFSP	-	loss
chr6	117073359	117086886	FAM162B	-	loss
chr6	117113247	117150198	GPRC6A	-	loss
chr6	117609529	117747018	ROS1	-	loss
chr6	117639350	117923705	GOPC	-	loss
chr6	118781934	119031238	CEP85L	-	loss
chr6	119134611	119256327	MCM9	-	loss
chr6	119280995	119470358	FAM184A	-	loss
chr6	119390211	119390308	MIR548B	-	loss
chr6	119498365	119670931	MAN1A1	-	loss
chr6	119773711	119812467	LOC285762	-	loss
chr8	182199	197339	ZNF596	+	loss
chr8	356807	419875	FBXO25	+	loss
chr8	687586	1087777	ERICH1-AS1	+	loss
chr8	1449531	1656642	DLGAP2	+	loss
chr8	1711869	1734736	CLN8	+	loss
chr8	1765396	1765473	MIR596	+	loss
chr8	1772148	1906807	ARHGEF10	+	loss

chr8	1922043	1955109	KBTBD11	+	loss
chr8	1993157	2093380	MYOM2	+	loss
chr8	6264112	6501140	MCPH1	+	loss
chr8	6565877	6619021	AGPAT5	+	loss
chr8	6602684	6602765	MIR4659A	+	loss
chr8	6693075	6699975	GS1-24F4.2	+	loss
chr8	7353367	7366833	DEFB107A	+	loss
chr8	7627105	7628835	FAM90A10P	+	loss
chr8	7705401	7721319	SPAG11A	+	loss
chr8	7752198	7754237	DEFB4A	+	loss
chr8	7783858	7809935	ZNF705B	+	loss
chr8	7812534	7866277	FAM66E	+	loss
chr8	8086091	8102387	FAM86B3P	+	loss
chr8	8559665	8561617	CLDN23	+	loss
chr8	8860313	8890849	ERI1	+	loss
chr8	8905954	8906028	MIR4660	+	loss
chr8	9182560	9192590	LOC157273	+	loss
chr8	9413444	9639856	TNKS	+	loss
chr8	9599181	9599278	MIR597	+	loss
chr8	9911829	10286401	MSRA	+	loss
chr8	10383055	10411676	PRSS55	+	loss
chr8	10530146	10558103	C8orf74	+	loss
chr8	11141999	11185654	MTMR9	+	loss
chr8	11188494	11189695	SLC35G5	+	loss
chr8	11197145	11225961	TDH	+	loss
chr8	11225910	11296166	C8orf12	+	loss
chr8	11351520	11422108	BLK	+	loss
chr8	11434043	11438850	LINC00208	+	loss
chr8	11534467	11617509	GATA4	+	loss
chr8	11627171	11644854	NEIL2	+	loss
chr8	11653081	11696818	FDFT1	+	loss
chr8	11839829	11842099	DEFB135	+	loss
chr8	11946846	11973025	ZNF705D	+	loss
chr8	11973290	12008698	FAM66D	+	loss
chr8	11973290	12424354	LOC100506990	+	loss
chr8	12219527	12279970	FAM66A	+	loss
chr8	12584745	12584808	MIR3926-2	+	loss
chr8	12651751	12675800	LINC00681	+	loss
chr8	12803150	12889012	KIAA1456	+	loss
chr8	13424351	13425797	C8orf48	+	loss
chr8	15397595	15624158	TUSC3	+	loss
chr8	16884746	16980148	MICU3	+	loss
chr8	17013835	17080241	ZDHHC2	+	loss
chr8	17104400	17155533	VPS37A	+	loss
chr8	17354596	17428077	SLC7A2	+	loss
chr8	17433941	17500642	PDGFRL	+	loss
chr8	17780365	17887457	PCM1	+	loss
chr8	18027970	18081198	NAT1	+	loss
chr8	18248754	18258723	NAT2	+	loss
chr8	19171080	19253729	SH2D4A	+	loss

chr8	19674917	19709586	INTS10	+	loss
chr8	19796581	19824770	LPL	+	loss
chr8	20054703	20079207	ATP6V1B2	+	loss
chr8	20133283	20147969	LZTS1-AS1	+	loss
chr8	20831496	20852630	LOC286114	+	loss
chr8	21777179	21864096	XPO7	+	loss
chr8	21881644	21894408	NPM2	+	loss
chr8	21900427	21906319	FGF17	+	loss
chr8	21911080	21940036	DMTN	+	loss
chr8	21946713	21961891	FAM160B2	+	loss
chr8	22016233	22021991	SFTPC	+	loss
chr8	22022652	22069840	BMP1	+	loss
chr8	22102618	22108680	POLR3D	+	loss
chr8	22132809	22213584	PIWIL2	+	loss
chr8	22224761	22291640	SLC39A14	+	loss
chr8	22298482	22398657	PPP3CC	+	loss
chr8	22409250	22433008	SORBS3	+	loss
chr8	22436253	22455538	PDLIM2	+	loss
chr8	22457113	22461662	C8orf58	+	loss
chr8	22462144	22477983	CCAR2	+	loss
chr8	22844929	22877710	RHOBTB2	+	loss
chr8	22925741	22941132	LOC286059	+	loss
chr8	22941867	22961070	LOC254896	+	loss
chr8	22960326	22974950	TNFRSF10C	+	loss
chr8	23082733	23088439	LOC389641	+	loss
chr8	23101149	23119512	CHMP7	+	loss
chr8	23145611	23153792	R3HCC1	+	loss
chr8	23193720	23223638	LOC100507156	+	loss
chr8	23386362	23430063	SLC25A37	+	loss
chr8	24151579	24212726	ADAM28	+	loss
chr8	24241797	24263526	ADAMDEC1	+	loss
chr8	24298508	24384483	ADAM7	+	loss
chr8	24771273	24776606	NEFM	+	loss
chr8	25042286	25270619	DOCK5	+	loss
chr8	25230074	26230195	PPP2R2A	+	loss
chr8	25316512	25365425	CDCA2	+	loss
chr8	26240522	26270644	BNIP3L	+	loss
chr8	26371708	26515693	DPYSL2	+	loss
chr8	27168998	27316908	PTK2B	+	loss
chr8	27348518	27402439	EPHX2	+	loss
chr8	27491576	27534286	SCARA3	+	loss
chr8	27559193	27559276	MIR3622A	+	loss
chr8	27632057	27670141	ESCO2	+	loss
chr8	27947748	28048669	ELP3	+	loss
chr8	28174648	28200868	PNOC	+	loss
chr8	28351721	28431785	FZD3	+	loss
chr8	28558989	28611207	EXTL3	+	loss
chr8	28747910	28910242	HMBOX1	+	loss
chr8	29779028	29811123	FAM183CP	+	loss
chr8	29952921	29995222	LEPROTL1	+	loss

chr8	30013812	30041155	DCTN6	+	loss
chr8	30241943	30429734	RBPMS	+	loss
chr8	30496116	30503469	SMIM18	+	loss
chr8	30601689	30624520	UBXN8	+	loss
chr8	30890777	31031277	WRN	+	loss
chr8	31496910	32622558	NRG1	+	loss
chr8	32298261	32300241	NRG1-IT3	+	loss
chr8	33342684	33358778	MAK16	+	loss
chr8	35092974	35652181	UNC5D	+	loss
chr8	36641841	36793643	KCNU1	+	loss
chr8	37553300	37556396	ZNF703	+	loss
chr8	37594096	37615319	ERLIN2	+	loss
chr8	37620100	37637286	PROSC	+	loss
chr8	37654400	37701504	GPR124	+	loss
chr8	37888019	37917883	EIF4EBP1	+	loss
chr8	37963010	37997598	ASH2L	+	loss
chr8	38034105	38070819	BAG4	+	loss
chr8	38089008	38120287	DDHD2	+	loss
chr8	38243958	38266062	LETM2	+	loss
chr8	38585703	38710546	TACC1	+	loss
chr8	38758752	38831430	PLEKHA2	+	loss
chr8	38831667	38846181	HTRA4	+	loss
chr8	38854504	38962779	ADAM9	+	loss
chr8	38965049	39142436	ADAM32	+	loss
chr8	39172181	39274897	ADAM5	+	loss
chr8	39420630	39437653	LOC100130964	+	loss
chr8	39442086	39587583	ADAM18	+	loss
chr8	39771327	39786309	IDO1	+	loss
chr8	39792473	39873910	IDO2	+	loss
chr8	40010986	40012827	C8orf4	+	loss
chr8	41348080	41368499	GOLGA7	+	loss
chr8	41386724	41402565	GINS4	+	loss
chr8	41435706	41482520	AGPAT6	+	loss
chr8	42010463	42028701	AP3M2	+	loss
chr8	42128819	42190171	IKBKB	+	loss
chr8	42195972	42229331	POLB	+	loss
chr8	42249278	42263455	VDAC3	+	loss
chr8	42396297	42408140	SMIM19	+	loss
chr8	42552561	42592209	CHRNB3	+	loss
chr8	42752032	42885682	HOOK3	+	loss
chr8	42911441	42940932	FNTA	+	loss
chr8	42948648	42978323	POMK	+	loss
chr8	42995591	43057970	HGSNAT	+	loss
chr8	43147584	43218328	POTEA	+	loss
chr8	158344	182318	RPL23AP53	-	loss
chr8	439789	495781	TDRP	-	loss
chr8	614199	681226	ERICH1	-	loss
chr8	1244293	1250827	LOC286083	-	loss
chr8	2792874	4852328	CSMD1	-	loss
chr8	6261076	6264069	LOC100287015	-	loss

chr8	6357174	6420784	ANGPT2	-	loss
chr8	6602688	6602761	MIR4659B	-	loss
chr8	6666040	6693166	XKR5	-	loss
chr8	6728096	6735529	DEFB1	-	loss
chr8	6782215	6783598	DEFA6	-	loss
chr8	6793344	6795786	DEFA4	-	loss
chr8	6808247	6809121	DEFA8P	-	loss
chr8	6816810	6817683	DEFA9P	-	loss
chr8	6825662	6826635	DEFA10P	-	loss
chr8	6886122	6887011	DEFA11P	-	loss
chr8	6912828	6914259	DEFA5	-	loss
chr8	7143732	7212876	FAM66B	-	loss
chr8	7272384	7274354	DEFB4B	-	loss
chr8	7413659	7431920	FAM90A7P	-	loss
chr8	7669241	7673238	DEFB107B	-	loss
chr8	7829182	7830775	USP17L8	-	loss
chr8	7833914	7835507	USP17L3	-	loss
chr8	7946462	7946611	MIR548I3	-	loss
chr8	8175257	8239257	SGK223	-	loss
chr8	8641998	8751131	MFHAS1	-	loss
chr8	8993763	9009152	PPP1R3B	-	loss
chr8	9757573	9760839	LINC00599	-	loss
chr8	9760897	9760982	MIR124-1	-	loss
chr8	10463859	10512617	RP1L1	-	loss
chr8	10581277	10697299	SOX7	-	loss
chr8	10622883	10697299	PINX1	-	loss
chr8	10682882	10682953	MIR1322	-	loss
chr8	10753656	11058875	XKR6	-	loss
chr8	10892715	10892812	MIR598	-	loss
chr8	11278972	11324276	FAM167A	-	loss
chr8	11700033	11725646	CTSB	-	loss
chr8	11831445	11832108	DEFB136	-	loss
chr8	11851488	11853760	DEFB134	-	loss
chr8	11921897	12175825	DEFB130	-	loss
chr8	11985366	11986806	LOC392196	-	loss
chr8	11989925	11991518	USP17L7	-	loss
chr8	11994676	11996269	USP17L2	-	loss
chr8	12030962	12034776	FAM90A2P	-	loss
chr8	12039612	12051624	FAM86B1	-	loss
chr8	12236775	12237298	LOC649352	-	loss
chr8	12250792	12257874	DEFB109P1	-	loss
chr8	12272029	12275541	FAM90A25P	-	loss
chr8	12283123	12293864	FAM86B2	-	loss
chr8	12394587	12523120	LOC729732	-	loss
chr8	12579405	12612992	LONRF1	-	loss
chr8	12584740	12584813	MIR3926-1	-	loss
chr8	12623570	12668910	LOC340357	-	loss
chr8	12940871	13372429	DLC1	-	loss
chr8	13947372	15095792	SGCZ	-	loss
chr8	14710946	14711019	MIR383	-	loss

chr8	15965386	16050300	MSR1	-	loss
chr8	16850333	16859674	FGF20	-	loss
chr8	17086739	17104387	CNOT7	-	loss
chr8	17154305	17271040	MTMR7	-	loss
chr8	17501302	17658426	MTUS1	-	loss
chr8	17721899	17767874	FGL1	-	loss
chr8	17913924	17942507	ASAH1	-	loss
chr8	18384812	18871196	PSD3	-	loss
chr8	19041185	19103032	LOC100128993	-	loss
chr8	19261671	19615382	CSGALNACT1	-	loss
chr8	20002365	20040717	SLC18A1	-	loss
chr8	20103675	20112803	LZTS1	-	loss
chr8	21549529	21646346	GFRA2	-	loss
chr8	21563901	21771205	DOK2	-	loss
chr8	21964382	21967456	NUDT18	-	loss
chr8	21971931	21988565	HR	-	loss
chr8	21995532	21999448	REEP4	-	loss
chr8	22004342	22014344	LGI3	-	loss
chr8	22077215	22089851	PHYHIP	-	loss
chr8	22102474	22102556	MIR320A	-	loss
chr8	22477946	22526661	BIN3	-	loss
chr8	22497883	22499722	BIN3-IT1	-	loss
chr8	22545173	22550815	EGR3	-	loss
chr8	22570764	22785421	PEBP4	-	loss
chr8	22877647	22926700	TNFRSF10B	-	loss
chr8	22993100	23021543	TNFRSF10D	-	loss
chr8	23048969	23082680	TNFRSF10A	-	loss
chr8	23154409	23261722	LOXL2	-	loss
chr8	23243441	23315244	ENTPD4	-	loss
chr8	23536205	23540450	NKX3-1	-	loss
chr8	23559963	23564111	NKX2-6	-	loss
chr8	23699433	23712320	STC1	-	loss
chr8	24808468	24814383	NEFL	-	loss
chr8	25276773	25282556	GNRH1	-	loss
chr8	25285363	25315984	KCTD9	-	loss
chr8	25699245	25902640	EBF2	-	loss
chr8	26362195	26371483	PNMA2	-	loss
chr8	26605666	26722922	ADRA1A	-	loss
chr8	27093813	27115903	STMN4	-	loss
chr8	27142403	27168834	TRIM35	-	loss
chr8	27317277	27336813	CHRNA2	-	loss
chr8	27454433	27472328	CLU	-	loss
chr8	27559189	27559284	MIR3622B	-	loss
chr8	27590832	27630170	CCDC25	-	loss
chr8	27667137	27695572	PBK	-	loss
chr8	27727398	27850369	SCARA5	-	loss
chr8	27743555	27743633	MIR4287	-	loss
chr8	27879480	27941388	NUGGC	-	loss
chr8	28203101	28347835	ZNF395	-	loss
chr8	28285925	28347835	FBXO16	-	loss

chr8	28362632	28362699	MIR4288	-	loss
chr8	28625174	28747698	INTS9	-	loss
chr8	28924794	29120610	KIF13B	-	loss
chr8	29190578	29208267	DUSP4	-	loss
chr8	29578775	29605625	LINC00589	-	loss
chr8	29814787	29814864	MIR3148	-	loss
chr8	29920257	30108213	MIR548O2	-	loss
chr8	29920630	29940649	SARAF	-	loss
chr8	29989186	30002200	MBOAT4	-	loss
chr8	30239634	30242917	RBPMS-AS1	-	loss
chr8	30436030	30515738	GTF2E2	-	loss
chr8	30535579	30585486	GSR	-	loss
chr8	30643125	30670352	PPP2CB	-	loss
chr8	30689059	30706533	TEX15	-	loss
chr8	30853320	30891231	PURG	-	loss
chr8	33228343	33330664	FUT10	-	loss
chr8	33330903	33370703	TTI2	-	loss
chr8	33405272	33424643	RNF122	-	loss
chr8	33448853	33457439	DUSP26	-	loss
chr8	37604073	37605564	LOC728024	-	loss
chr8	37701397	37707431	BRF2	-	loss
chr8	37716464	37757015	RAB11FIP1	-	loss
chr8	37791799	37797647	GOT1L1	-	loss
chr8	37820513	37824184	ADRB3	-	loss
chr8	38000217	38008600	STAR	-	loss
chr8	38020838	38034248	LSM1	-	loss
chr8	38120649	38126738	PPAPDC1B	-	loss
chr8	38132560	38239790	WHSC1L1	-	loss
chr8	38268655	38326352	FGFR1	-	loss
chr8	38368351	38386180	C8orf86	-	loss
chr8	38457692	38458775	RNF5P1	-	loss
chr8	38846326	38854041	TM2D2	-	loss
chr8	39601254	39695808	ADAM2	-	loss
chr8	40388110	40755343	ZMAT4	-	loss
chr8	41119475	41166990	SFRP1	-	loss
chr8	41503828	41643385	NKX6-3	-	loss
chr8	41510743	41754280	ANK1	-	loss
chr8	41517958	41518026	MIR486-1	-	loss
chr8	41786996	41909505	KAT6A	-	loss
chr8	42032235	42065194	PLAT	-	loss
chr8	42231585	42234674	DKK4	-	loss
chr8	42273979	42397356	SLC20A2	-	loss
chr8	42607779	42623929	CHRNA6	-	loss
chr8	42691816	42698474	THAP1	-	loss
chr8	42704779	42751866	RNF170	-	loss
chr8	42751339	42751418	MIR4469	-	loss
chr10	88414313	88426605	OPN4	+	loss
chr10	88426550	88495824	LDB3	+	loss
chr10	88516395	88684945	BMPR1A	+	loss
chr10	88718287	88723017	SNCG	+	loss

chr10	88728187	88730666	ADIRF	+	loss
chr10	88730497	88784487	AGAP11	+	loss
chr10	88780045	88784487	FAM25A	+	loss
chr10	88854952	88951222	FAM35A	+	loss
chr10	88985204	88994733	NUTM2A	+	loss
chr10	89102167	89103331	LOC439994	+	loss
chr10	89117476	89130452	NUTM2D	+	loss
chr10	89263637	89263711	MIR4678	+	loss
chr10	89264222	89313218	MINPP1	+	loss
chr10	89419475	89507462	PAPSS2	+	loss
chr10	89578069	89605369	CFL1P1	+	loss
chr10	89623194	89728532	PTEN	+	loss
chr10	90346518	90366733	LIPJ	+	loss
chr10	90424145	90438572	LIPF	+	loss
chr10	90484300	90512513	LIPK	+	loss
chr10	90521162	90537999	LIPN	+	loss
chr10	90562486	90580303	LIPM	+	loss
chr10	90639943	90734910	STAMBPL1	+	loss
chr10	90729552	90775542	FAS	+	loss
chr10	90823092	90823167	MIR4679-1	+	loss
chr10	88695297	88717425	MMRN2	-	loss
chr10	88809958	88854776	GLUD1	-	loss
chr10	88965544	89102315	NUTM2A-AS1	-	loss
chr10	89156330	89167457	LINC00864	-	loss
chr10	89512874	89577917	ATAD1	-	loss
chr10	89618917	89623194	KLLN	-	loss
chr10	89892056	90343082	RNLS	-	loss
chr10	90579658	90611732	ANKRD22	-	loss
chr10	90694830	90751147	ACTA2	-	loss
chr10	90751180	90752732	FAS-AS1	-	loss
chr10	90823091	90823168	MIR4679-2	-	loss
chr10	90965693	90967071	CH25H	-	loss
chr10	90973325	91174382	LIPA	-	loss
chr12	11081833	11087444	PRH2	+	loss
chr12	11323779	11328619	LOC100129361	+	loss
chr12	11700963	11717335	LINC01252	+	loss
chr12	11802787	12268261	ETV6	+	loss
chr12	12223877	12363947	BCL2L14	+	loss
chr12	12510019	12619838	LOH12CR1	+	loss
chr12	12764830	12798041	CREBL2	+	loss
chr12	12870301	12875305	CDKN1B	+	loss
chr12	12878850	12944399	APOLD1	+	loss
chr12	12878850	12982915	DDX47	+	loss
chr12	12917582	12917677	MIR613	+	loss
chr12	13028410	13029070	RPL13AP20	+	loss
chr12	13043955	13066600	GPRC5A	+	loss
chr12	13068762	13068852	MIR614	+	loss
chr12	13132770	13137576	LOC100506314	+	loss
chr12	13153375	13157764	HTR7P1	+	loss
chr12	13197314	13236383	KIAA1467	+	loss

chr12	13349601	13369708	EMP1	+	loss
chr12	14518610	14651697	ATF7IP	+	loss
chr12	14927269	14930936	H2AFJ	+	loss
chr12	14956505	14976791	C12orf60	+	loss
chr12	15125955	15134799	PDE6H	+	loss
chr12	10771537	10826891	STYK1	-	loss
chr12	10851675	10875953	YBX3	-	loss
chr12	10954130	10955226	TAS2R7	-	loss
chr12	10958649	10959579	TAS2R8	-	loss
chr12	10961692	10962767	TAS2R9	-	loss
chr12	10977944	10978868	TAS2R10	-	loss
chr12	10998447	11002075	PRR4	-	loss
chr12	10998447	11324224	PRH1-PRR4	-	loss
chr12	11033559	11036874	PRH1	-	loss
chr12	11033559	11463369	PRB4	-	loss
chr12	11060524	11062161	TAS2R13	-	loss
chr12	11090852	11091806	TAS2R14	-	loss
chr12	11138511	11139511	TAS2R50	-	loss
chr12	11148560	11150474	TAS2R20	-	loss
chr12	11174270	11175170	TAS2R19	-	loss
chr12	11182985	11184006	TAS2R31	-	loss
chr12	11213963	11214893	TAS2R46	-	loss
chr12	11243885	11244912	TAS2R43	-	loss
chr12	11285883	11286843	TAS2R30	-	loss
chr12	11338598	11339543	TAS2R42	-	loss
chr12	11418856	11422641	PRB3	-	loss
chr12	11504756	11508524	PRB1	-	loss
chr12	11544473	11548498	PRB2	-	loss
chr12	12268960	12419811	LRP6	-	loss
chr12	12482217	12503169	MANSC1	-	loss
chr12	12508341	12510001	LOH12CR2	-	loss
chr12	12626215	12715448	DUSP16	-	loss
chr12	12813994	12849121	GPR19	-	loss
chr12	13093708	13103318	GPRC5D	-	loss
chr12	13127798	13153243	HEBP1	-	loss
chr12	13236470	13256630	GSG1	-	loss
chr12	13523604	13529679	C12orf36	-	loss
chr12	13714409	14133022	GRIN2B	-	loss
chr12	14656596	14720791	PLBD1	-	loss
chr12	14765567	14849519	GUCY2C	-	loss
chr12	14923653	14924065	HIST4H4	-	loss
chr12	14939411	14956401	WBP11	-	loss
chr12	14957583	14967116	SMCO3	-	loss
chr12	14982244	14996413	ART4	-	loss
chr12	15034114	15038853	MGP	-	loss
chr12	15066975	15091463	ERP27	-	loss
chr12	15094949	15114562	ARHGDIB	-	loss
chr13	35516423	36246874	NBEA	+	loss
chr13	36920567	36943872	SPG20OS	+	loss
chr13	37005656	37017019	CCNA1	+	loss

chr13	37248048	37271975	SERTM1	+	loss
chr13	37393338	37403740	RFXAP	+	loss
chr13	37574677	37583751	EXOSC8	+	loss
chr13	38109076	38125673	LINC00547	+	loss
chr13	38923941	38937143	UFM1	+	loss
chr13	39141860	39153653	LINC00366	+	loss
chr13	39261172	39461267	FREM2	+	loss
chr13	39612447	39624244	NHLRC3	+	loss
chr13	40229763	40365802	COG6	+	loss
chr13	40755945	40763167	LINC00332	+	loss
chr13	41363546	41386596	SLC25A15	+	loss
chr13	41384901	41384997	MIR621	+	loss
chr13	41635696	41658139	WBP4	+	loss
chr13	41885340	41951166	NAA16	+	loss
chr13	42016699	42017902	OR7E37P	+	loss
chr13	42031541	42045013	RGCC	+	loss
chr13	42535304	42555701	VWA8-AS1	+	loss
chr13	42614171	42830716	DGKH	+	loss
chr13	42846288	42897403	AKAP11	+	loss
chr13	43136871	43182149	TNFSF11	+	loss
chr13	43355685	43365685	FAM216B	+	loss
chr13	43597361	43683306	DNAJC15	+	loss
chr13	44453419	44468068	LACC1	+	loss
chr13	44596470	44604599	LINC00284	+	loss
chr13	44947977	44971850	SERP2	+	loss
chr13	45563686	45607743	GPALPP1	+	loss
chr13	45694630	45858239	GTF2F2	+	loss
chr13	45915479	45965618	TPT1-AS1	+	loss
chr13	46039029	46110833	COG3	+	loss
chr13	46276445	46288693	SPERT	+	loss
chr13	46626982	46675482	CPB2-AS1	+	loss
chr13	47127295	47327175	LRCH1	+	loss
chr13	48611702	48621282	NUDT15	+	loss
chr13	48651272	48654129	MED4-AS1	+	loss
chr13	48807273	48836232	ITM2B	+	loss
chr13	48877882	49056026	RB1	+	loss
chr13	49280952	49283498	CYSLTR2	+	loss
chr13	49550047	49783915	FNDC3A	+	loss
chr13	49794473	49796513	MLNR	+	loss
chr13	49822046	49867622	CDADC1	+	loss
chr13	50018428	50069139	SETDB2	+	loss
chr13	50069800	50103117	PHF11	+	loss
chr13	50202434	50208008	ARL11	+	loss
chr13	50464544	50467516	CTAGE10P	+	loss
chr13	50571142	50592603	TRIM13	+	loss
chr13	50589389	50595058	KCNRG	+	loss
chr13	50656413	51102670	DLEU1	+	loss
chr13	50746153	50747751	ST13P4	+	loss
chr13	51381990	51424041	DLEU7-AS1	+	loss
chr13	51483813	51544596	RNASEH2B	+	loss

chr13	51796469	51858377	FAM124A	+	loss
chr13	51915167	51938418	SERPINE3	+	loss
chr13	52126724	52126803	MIR4703	+	loss
chr13	52158483	52336171	WDFY2	+	loss
chr13	52436116	52440372	CCDC70	+	loss
chr13	52586522	52591849	ALG11	+	loss
chr13	52586522	52607736	UTP14C	+	loss
chr13	53029494	53050763	CKAP2	+	loss
chr13	53063127	53161225	TPTE2P3	+	loss
chr13	53191604	53217919	HNRNPA1L2	+	loss
chr13	53226830	53262433	SUGT1	+	loss
chr13	53384184	53384275	MIR759	+	loss
chr13	53602875	53626196	OLFM4	+	loss
chr13	54389553	54450254	LINC00558	+	loss
chr13	55748588	55748683	MIR5007	+	loss
chr13	57715051	57731216	PRR20D	+	loss
chr13	57734765	57737787	PRR20B	+	loss
chr13	57741330	57744352	PRR20E	+	loss
chr13	58205788	58303065	PCDH17	+	loss
chr13	60586884	60618490	DIAPH3-AS1	+	loss
chr13	60718831	60727639	DIAPH3-AS2	+	loss
chr13	60970590	61148013	TDRD3	+	loss
chr13	64311567	64316701	OR7E156P	+	loss
chr13	67399300	67489163	PCDH9-AS2	+	loss
chr13	67551520	67559908	PCDH9-AS3	+	loss
chr13	70681344	70713885	ATXN8OS	+	loss
chr13	71589272	71742549	LINC00348	+	loss
chr13	73302041	73330328	BORA	+	loss
chr13	73356229	73590591	PIBF1	+	loss
chr13	73629113	73651676	KLF5	+	loss
chr13	74993309	75009296	LINC00381	+	loss
chr13	75126979	75131257	LINC00347	+	loss
chr13	76123615	76180156	UCHL3	+	loss
chr13	76194569	76434006	LMO7	+	loss
chr13	76445173	76457948	LMO7DN	+	loss
chr13	77502584	77503224	BTF3P11	+	loss
chr13	77522693	77532776	IRG1	+	loss
chr13	77566058	77576652	CLN5	+	loss
chr13	77649648	77661911	MYCBP2-AS1	+	loss
chr13	78109808	78219398	SCEL	+	loss
chr13	78271988	78338377	SLAIN1	+	loss
chr13	78493823	79191460	RNF219-AS1	+	loss
chr13	78587017	78627730	LINC00446	+	loss
chr13	36047925	36515382	MIR548F5	-	loss
chr13	36342788	36705514	DCLK1	-	loss
chr13	36742344	36788752	SOHLH2	-	loss
chr13	36742344	36871992	CCDC169-SOHLH	-	loss
chr13	36801178	36871992	CCDC169	-	loss
chr13	36875774	36944317	SPG20	-	loss
chr13	37418967	37494409	SMAD9	-	loss

chr13	37523907	37573504	ALG5	-	loss
chr13	37583450	37633850	SUPT20H	-	loss
chr13	37677396	37679801	CSNK1A1L	-	loss
chr13	38136718	38183563	POSTN	-	loss
chr13	38210772	38443939	TRPC4	-	loss
chr13	38624953	38717369	LINC00571	-	loss
chr13	39540061	39564996	STOML3	-	loss
chr13	39584001	39612213	PROSER1	-	loss
chr13	39917028	40177356	LHFP	-	loss
chr13	40238170	40238272	MIR4305	-	loss
chr13	40768645	40794639	LINC00548	-	loss
chr13	40921270	41055143	LINC00598	-	loss
chr13	41048130	41240734	FOXO1	-	loss
chr13	41301963	41302011	MIR320D1	-	loss
chr13	41303431	41345347	MRPS31	-	loss
chr13	41371120	41495910	TPTE2P5	-	loss
chr13	41486024	41495910	SUGT1P3	-	loss
chr13	41506054	41593508	ELF1	-	loss
chr13	41701708	41706936	KBTBD6	-	loss
chr13	41765710	41768702	KBTBD7	-	loss
chr13	41790515	41837742	MTRF1	-	loss
chr13	42140960	42535221	VWA8	-	loss
chr13	42142421	42142531	MIR5006	-	loss
chr13	43460523	43566407	EPSTI1	-	loss
chr13	43787665	44361116	ENOX1	-	loss
chr13	44410488	44453826	CCDC122	-	loss
chr13	44720605	44732358	SMIM2-IT1	-	loss
chr13	45006278	45150701	TSC22D1	-	loss
chr13	45373639	45383766	LINC00330	-	loss
chr13	45513383	45563613	NUFIP1	-	loss
chr13	45766987	45775175	KCTD4	-	loss
chr13	45911303	45915297	TPT1	-	loss
chr13	45911614	45911744	SNORA31	-	loss
chr13	45967453	45992516	SLC25A30	-	loss
chr13	46115431	46189874	ERICH6B	-	loss
chr13	46354415	46425846	SIAH3	-	loss
chr13	46529804	46626894	ZC3H13	-	loss
chr13	46627321	46679211	CPB2	-	loss
chr13	46700057	46756459	LCP1	-	loss
chr13	46870579	46871979	LINC00563	-	loss
chr13	46916136	47012325	KIAA0226L	-	loss
chr13	47345390	47371367	ESD	-	loss
chr13	47405676	47471211	HTR2A	-	loss
chr13	48516790	48612125	SUCLA2	-	loss
chr13	48649863	48669277	MED4	-	loss
chr13	48870648	48877797	LINC00441	-	loss
chr13	48963706	49018840	LPAR6	-	loss
chr13	49063098	49110118	RCBTB2	-	loss
chr13	49151109	49155037	LINC00462	-	loss
chr13	49882785	50018221	CAB39L	-	loss

chr13	50106081	50159719	RCBTB1	-	loss
chr13	50234809	50265623	EBPL	-	loss
chr13	50273442	50367057	KPNA3	-	loss
chr13	50486841	50510625	SPRYD7	-	loss
chr13	50556687	50699677	DLEU2	-	loss
chr13	50570550	50570637	MIR3613	-	loss
chr13	50623108	50623337	MIR16-1	-	loss
chr13	51286758	51417885	DLEU7	-	loss
chr13	51456514	51484848	RNASEH2B-AS1	-	loss
chr13	51568646	51654998	GUCY1B2	-	loss
chr13	51658150	51746524	LINC00371	-	loss
chr13	51922702	51922775	MIR5693	-	loss
chr13	51935700	52027275	INTS6	-	loss
chr13	52342128	52378298	DHRS12	-	loss
chr13	52387482	52419286	LINC00282	-	loss
chr13	52506805	52585630	ATP7B	-	loss
chr13	52638899	52703214	NEK5	-	loss
chr13	52706778	52733996	NEK3	-	loss
chr13	52741844	52865680	MRPS31P5	-	loss
chr13	52951302	52980629	THSD1	-	loss
chr13	52986736	53024763	VPS36	-	loss
chr13	53277399	53313947	LECT1	-	loss
chr13	53418108	53422775	PCDH8	-	loss
chr13	54886106	54886183	MIR1297	-	loss
chr13	60239720	60738119	DIAPH3	-	loss
chr13	61773931	61774014	MIR3169	-	loss
chr13	61983818	62002079	PCDH20	-	loss
chr13	66876965	67804468	PCDH9	-	loss
chr13	69435416	69459457	LINC00550	-	loss
chr13	70274724	70682625	KLHL1	-	loss
chr13	72012097	72441330	DACH1	-	loss
chr13	73282494	73301938	MZT1	-	loss
chr13	73329539	73356344	DIS3	-	loss
chr13	74260148	74708394	KLF12	-	loss
chr13	75811888	75814517	CTAGE11P	-	loss
chr13	75858808	76056250	TBC1D4	-	loss
chr13	76099349	76111991	COMMD6	-	loss
chr13	77454303	77460540	KCTD12	-	loss
chr13	77579388	77601331	FBXL3	-	loss
chr13	77618791	77901177	MYCBP2	-	loss
chr13	78272146	78272251	MIR3665	-	loss
chr13	78469615	78549664	EDNRB	-	loss
chr13	79173229	79177695	POU4F1	-	loss
chr13	79188420	79234700	RNF219	-	loss
chr13	79361453	79414185	LINC00331	-	loss
chr16	74330672	74340186	PSMD7	+	loss
chr16	75032914	75144892	ZNRF1	+	loss
chr16	75182420	75206132	ZFP1	+	loss
chr16	75252883	75258822	CTRB1	+	loss
chr16	75600248	75611779	GABARAPL2	+	loss

chr16	75681634	75691341	TERF2IP	+	loss
chr16	76311175	76593135	CNTNAP4	+	loss
chr16	76902832	76902916	MIR4719	+	loss
chr16	77224835	77233543	MON1B	+	loss
chr16	77233348	77246976	SYCE1L	+	loss
chr16	77756388	77776157	NUDT7	+	loss
chr16	77822482	78014001	VAT1L	+	loss
chr16	78056442	78066001	CLEC3A	+	loss
chr16	78133326	79246564	WWOX	+	loss
chr16	80574853	80584541	DYNLRB2	+	loss
chr16	81040102	81066709	CENPN	+	loss
chr16	81069457	81080951	ATMIN	+	loss
chr16	81272295	81324747	BCO1	+	loss
chr16	81348570	81413803	GAN	+	loss
chr16	81418622	81418698	MIR4720	+	loss
chr16	81478774	81745367	CMIP	+	loss
chr16	81698958	81700879	LOC100129617	+	loss
chr16	81812898	81991899	PLCG2	+	loss
chr16	82068857	82132139	HSD17B2	+	loss
chr16	82660398	83830215	CDH13	+	loss
chr16	83541950	83542013	MIR3182	+	loss
chr16	83841507	83846607	HSBP1	+	loss
chr16	83932729	83949787	MLYCD	+	loss
chr16	83982671	83999937	OSGIN1	+	loss
chr16	84002236	84036379	NECAB2	+	loss
chr16	84178864	84211524	DNAAF1	+	loss
chr16	84224722	84230772	ADAD2	+	loss
chr16	84328400	84363450	WFDC1	+	loss
chr16	84402132	84497793	ATP2C2	+	loss
chr16	84682130	84695916	KLHL36	+	loss
chr16	84733554	84813527	USP10	+	loss
chr16	84853586	84943116	CRISPLD2	+	loss
chr16	85061374	85127828	KIAA0513	+	loss
chr16	85170755	85183049	LOC400548	+	loss
chr16	85316563	85321685	LINC00311	+	loss
chr16	85645028	85709812	GSE1	+	loss
chr16	85833172	85840607	COX4I1	+	loss
chr16	85932773	85956211	IRF8	+	loss
chr16	86320036	86326995	LOC146513	+	loss
chr16	86544132	86548070	FOXF1	+	loss
chr16	86588925	86590905	FLJ30679	+	loss
chr16	86600856	86602537	FOXC2	+	loss
chr16	86612114	86615304	FOXL1	+	loss
chr16	87425800	87438380	MAP1LC3B	+	loss
chr16	87635440	87731761	JPH3	+	loss
chr16	87984237	88110924	BANP	+	loss
chr16	88493878	88507165	ZNF469	+	loss
chr16	88520013	88601574	ZFPM1	+	loss
chr16	88535325	88535439	MIR5189	+	loss
chr16	88636788	88698372	ZC3H18	+	loss

chr16	88705000	88706882	IL17C	+	loss
chr16	88729780	88753594	SNAI3-AS1	+	loss
chr16	88772890	88781786	CTU2	+	loss
chr16	88870185	88875666	CDT1	+	loss
chr16	88922627	88927591	TRAPPC2L	+	loss
chr16	89160216	89222171	ACSF3	+	loss
chr16	89225627	89230083	LINC00304	+	loss
chr16	89232790	89235555	LOC400558	+	loss
chr16	89238162	89261900	CDH15	+	loss
chr16	89284110	89295965	ZNF778	+	loss
chr16	89387540	89391518	LOC100287036	+	loss
chr16	89574804	89624174	SPG7	+	loss
chr16	89627064	89633237	RPL13	+	loss
chr16	89627837	89627909	SNORD68	+	loss
chr16	89642175	89663654	CPNE7	+	loss
chr16	89679715	89704839	DPEP1	+	loss
chr16	89724151	89736866	SPATA33	+	loss
chr16	89753075	89762772	CDK10	+	loss
chr16	89778263	89784573	VPS9D1-AS1	+	loss
chr16	89786807	89807332	ZNF276	+	loss
chr16	89894906	89937727	SPIRE2	+	loss
chr16	89939993	89977792	TCF25	+	loss
chr16	89984286	89987385	MC1R	+	loss
chr16	89985258	90002505	TUBB3	+	loss
chr16	90015138	90034468	DEF8	+	loss
chr16	90038987	90067195	AFG3L1P	+	loss
chr16	90086068	90111379	GAS8	+	loss
chr16	74366303	74402153	LOC283922	-	loss
chr16	74442528	74455649	CLEC18B	-	loss
chr16	74481325	74641042	GLG1	-	loss
chr16	74907470	75019017	WDR59	-	loss
chr16	75145757	75150670	LDHD	-	loss
chr16	75237993	75241072	CTRB2	-	loss
chr16	75262927	75301951	BCAR1	-	loss
chr16	75327607	75467387	CFDP1	-	loss
chr16	75477141	75498584	TMEM170A	-	loss
chr16	75507021	75528926	CHST6	-	loss
chr16	75562427	75579393	CHST5	-	loss
chr16	75572014	75590184	TMEM231	-	loss
chr16	75632246	75657221	ADAT1	-	loss
chr16	75661621	75682541	KARS	-	loss
chr16	77316024	77469011	ADAMTS18	-	loss
chr16	79627744	79634622	MAF	-	loss
chr16	80637675	80838175	CDYL2	-	loss
chr16	81009698	81040502	CMC2	-	loss
chr16	81087101	81110872	C16orf46	-	loss
chr16	81115551	81129980	GCSH	-	loss
chr16	81134483	81253975	PKD1L2	-	loss
chr16	82031250	82045093	SDR42E1	-	loss
chr16	82181766	82203829	MPHOSPH6	-	loss

chr16	84043388	84075762	SLC38A8	-	loss
chr16	84087368	84150517	MBTPS1	-	loss
chr16	84155743	84178800	HSDL1	-	loss
chr16	84211452	84220676	TAF1C	-	loss
chr16	84254740	84273356	KCNG4	-	loss
chr16	84509965	84538288	TLDC1	-	loss
chr16	84599203	84651669	COTL1	-	loss
chr16	85008066	85045141	ZDHHC7	-	loss
chr16	85131964	85146114	FAM92B	-	loss
chr16	85339831	85339931	MIR5093	-	loss
chr16	85711279	85722588	GINS2	-	loss
chr16	85741123	85784689	C16orf74	-	loss
chr16	85775226	85775306	MIR1910	-	loss
chr16	85812230	85833148	EMC8	-	loss
chr16	86365455	86379285	LINC00917	-	loss
chr16	86508130	86542466	FENDRR	-	loss
chr16	86563781	86588841	MTHFSD	-	loss
chr16	87336403	87351026	C16orf95	-	loss
chr16	87362941	87425708	FBXO31	-	loss
chr16	87439851	87525460	ZCCHC14	-	loss
chr16	87736728	87799598	KLHDC4	-	loss
chr16	87863628	87903100	SLC7A5	-	loss
chr16	87921624	87970112	CA5A	-	loss
chr16	88709696	88717492	CYBA	-	loss
chr16	88718347	88729495	MVD	-	loss
chr16	88744089	88752882	SNAI3	-	loss
chr16	88762902	88772829	RNF166	-	loss
chr16	88781745	88851372	PIEZO1	-	loss
chr16	88782685	88782745	MIR4722	-	loss
chr16	88875876	88878342	APRT	-	loss
chr16	88880141	88923374	GALNS	-	loss
chr16	88928035	88933068	PABPN1L	-	loss
chr16	88941262	89043504	CBFA2T3	-	loss
chr16	89262168	89266529	SLC22A31	-	loss
chr16	89334028	89556969	ANKRD11	-	loss
chr16	89710838	89724193	CHMP1A	-	loss
chr16	89762764	89768121	SPATA2L	-	loss
chr16	89773540	89787394	VPS9D1	-	loss
chr16	89803958	89883065	FANCA	-	loss
chr16	90036182	90039240	CENPBD1	-	loss
chr16	90071278	90086328	DBNDD1	-	loss
chr16	90095315	90096309	C16orf3	-	loss
chr16	90106168	90114191	URAHP	-	loss
chr16	90122973	90142338	PRDM7	-	loss
chr21	40177230	40196878	ETS2	+	loss
chr21	40687632	40695144	BRWD1-AS1	+	loss
chr21	40752212	40786214	WRB	+	loss
chr21	40817796	40887433	SH3BGR	+	loss
chr21	40928368	41034815	B3GALT5	+	loss
chr21	41117333	41174023	IGSF5	+	loss

chr21	41239346	41301322	PCP4	+	loss
chr21	41755009	41757285	DSCAM-AS1	+	loss
chr21	42539483	42539556	MIR3197	+	loss
chr21	42539727	42648524	BACE2	+	loss
chr21	42676176	42729654	FAM3B	+	loss
chr21	42733949	42780869	MX2	+	loss
chr21	42792519	42831141	MX1	+	loss
chr21	39739182	40033704	ERG	-	loss
chr21	40110878	40145401	LINC00114	-	loss
chr21	40547371	40555440	PSMG1	-	loss
chr21	40557403	40685712	BRWD1	-	loss
chr21	40714240	40721047	HMGN1	-	loss
chr21	40777769	40817670	LCA5L	-	loss
chr21	40969074	40984749	C21orf88	-	loss
chr21	41384342	42219039	DSCAM	-	loss
chr21	41584278	41584358	MIR4760	-	loss
chr21	41990432	42002693	DSCAM-IT1	-	loss
chr21	42513426	42519991	LINC00323	-	loss
chr21	42547157	42557166	PLAC4	-	loss
chrY	14774297	14804153	TTTY15	+	loss
chrY	14813159	14972768	USP9Y	+	loss
chrY	15016018	15032390	DDX3Y	+	loss
chrY	15815446	15817902	TMSB4Y	+	loss
chrY	16634487	16955848	NLGN4Y	+	loss
chrY	21729235	21769056	TXLNGY	+	loss
chrY	22737610	22755040	EIF1AY	+	loss
chrY	22917953	22942918	RPS4Y2	+	loss
chrY	24455005	24462352	RBMY2FP	+	loss
chrY	24455005	24564028	RBMY1F	+	loss
chrY	24636543	24660784	PRY	+	loss
chrY	25365580	27053187	DAZ2	+	loss
chrY	14517914	14533389	GYG2P1	-	loss
chrY	15346460	15592550	UTY	-	loss
chrY	16905521	16915913	NLGN4Y-AS1	-	loss
chrY	21094584	21239302	TTTY14	-	loss
chrY	21152525	21154705	CD24	-	loss
chrY	21617316	21665039	BCORP1	-	loss
chrY	21867300	21906825	KDM5D	-	loss
chrY	22627553	22681114	TTTY10	-	loss
chrY	23557033	23563448	RBMY2EP	-	loss
chrY	23745485	23756552	TTTY13	-	loss
chrY	24026222	24329089	RBMY1E	-	loss
chrY	24217902	24242154	PRY2	-	loss
chrY	24442944	24445023	TTTY5	-	loss
chrY	25275501	25345254	DAZ1	-	loss

:oSNP)

Supplementary Table 5. Top 100 genes with the most highly variable inter-tumour expression

Gene ID	Ensembl ID	chromosome
ABCC4	ENSG00000125257	chr13q32.1
ACTC1	ENSG00000159251	chr15q14
ADIRF	ENSG00000148671	chr10q23.2
AEBP1	ENSG00000106624	chr7p13
ALOX15B	ENSG00000179593	chr17p13.1
AMACR	ENSG00000242110	chr5p13.2
ANGPTL4	ENSG00000167772	chr19p13.2
ANPEP	ENSG00000166825	chr15q26.1
AOC1	ENSG00000002726	chr7q36.1
ATF3	ENSG00000162772	chr1q32.3
C11orf96	ENSG00000187479	chr11p11.2
C15orf48	ENSG00000166920	chr15q21.1
C1orf64	ENSG00000183888	chr1p36.13
CCL2	ENSG00000108691	chr17q12
CES1	ENSG00000262243	chr16q12.2
CFB	ENSG00000239754	chr6p21.33
CLDN1	ENSG00000163347	chr3q28
CLDN8	ENSG00000156284	chr21q22.11
CNTNAP2	ENSG00000174469	chr7q35
COL1A1	ENSG00000108821	chr17q21.33
COL6A2	ENSG00000142173	chr21q22.3
COL8A1	ENSG00000144810	chr3q12.1
COL9A2	ENSG00000049089	chr1p34.2
COMP	ENSG00000105664	chr19p13.11
CYP27A1	ENSG00000135929	chr2q35
CYP2J2	ENSG00000134716	chr1p32.1
CYR61	ENSG00000142871	chr1p22.3
DLX1	ENSG00000144355	chr2q31.1
DNASE2B	ENSG00000137976	chr1p31.1
EEF1A2	ENSG00000101210	chr20q13.33
EGR1	ENSG00000120738	chr5q31.2
ERAP2	ENSG00000164308	chr5q15
FAM3D	ENSG00000198643	chr3p14.2
FBLN1	ENSG00000077942	chr22q13.31
FOS	ENSG00000170345	chr14q24.3
FOSB	ENSG00000125740	chr19q13.32
GDF15	ENSG00000130513	chr19p13.11
GNMT	ENSG00000124713	chr6p21.1
GP2	ENSG00000169347	chr16p12.3
GSTT1	ENSG00000277656	chr22q11.23
H19	ENSG00000130600	chr11p15.5
HMGCS2	ENSG00000134240	chr1p12
HSD17B6	ENSG00000025423	chr12q13.3
IGFBP6	ENSG00000167779	chr12q13.13
IGJ	ENSG00000132465	chr4q13.3
KCNN2	ENSG00000080709	chr5q22.3
KCNN4	ENSG00000104783	chr19q13.31
KLK12	ENSG00000186474	chr19q13.41

KRT15	ENSG00000171346	chr17q21.2
LTBP4	ENSG00000090006	chr19q13.2
MARCH6	ENSG00000145495	chr5p15.2
MESP1	ENSG00000166823	chr15q26.1
MIPEP	ENSG00000027001	chr13q12.12
MME	ENSG00000196549	chr3q25.2
MMP7	ENSG00000137673	chr11q22.2
MS4A8	ENSG00000166959	chr11q12.2
MSMB	ENSG00000263639	chr10q11.22
MT1G	ENSG00000125144	chr16q13
MT1M	ENSG00000205364	chr16q13
MUC1	ENSG00000185499	chr1q22
MYBPC1	ENSG00000196091	chr12q23.2
NCAPD3	ENSG00000151503	chr11q25
NFKBIZ	ENSG00000144802	chr3q12.3
OLFM4	ENSG00000102837	chr13q14.3
OR51E1	ENSG00000180785	chr11p15.4
ORM2	ENSG00000228278	chr9q32
PAGE4	ENSG00000101951	chrXp11.23
PCA3	ENSG00000225937	chr9q21.2
PCAT4	ENSG00000251321	chr4q21.21
PCGEM1	ENSG00000227418	chr2q32.3
PDE8B	ENSG00000113231	chr5q13.3
PGC	ENSG00000096088	chr6p21.1
PI16	ENSG00000164530	chr6p21.2
PIGR	ENSG00000162896	chr1q32.1
PLA2G2A	ENSG00000188257	chr1p36.13
PLA2G7	ENSG00000146070	chr6p12.3
PLAC9	ENSG00000189129	chr10q22.3
PPAP2A	ENSG00000067113	chr5q11.2
PTGS2	ENSG00000073756	chr1q31.1
RLN1	ENSG00000107018	chr9p24.1
RPL14	ENSG00000188846	chr3p22.1
RPL17	ENSG00000265681	chr18q21.1
S100P	ENSG00000163993	chr4p16.1
SCIN	ENSG00000006747	chr7p21.3
SCUBE2	ENSG00000175356	chr11p15.4
SERPINA3	ENSG00000196136	chr14q32.13
SNAP91	ENSG00000065609	chr6q14.2
SORD	ENSG00000140263	chr15q21.1
SPON2	ENSG00000159674	chr4p16.3
STXBP6	ENSG00000168952	chr14q12
TFF3	ENSG00000160180	chr21q22.3
TMEFF2	ENSG00000144339	chr2q32.3
TMEM178A	ENSG00000152154	chr2p22.1
TNFRSF19	ENSG00000127863	chr13q12.12
TUBB2A	ENSG00000137267	chr6p25.2
VSIG2	ENSG00000019102	chr11q24.2
ZFP36	ENSG00000128016	chr19q13.2
ZNF385B	ENSG00000144331	chr2q31.3

LEPREL1	ENSG00000090530	chr3q29
PDIA3P	ENSG00000180867	chr1q21.1

Supplementary Table 6. Discriminating 100 gene set, derived from gene expression and copy number feature data*

Chromosome	start position^	end position	Associated gene	% CNAs in cohort
chr8p21.3	23119071	23119120	CHMP7	37%
chr8p21.3	23153663	23153712	R3HCC1	37%
chr8p21.3	22432879	22432928	SORBS3	36%
chr8p21.3	22477693	22477742	CCAR2	36%
chr8p21.3	22461341	22461390	C8orf58	36%
chr8p21.3	21961474	21961523	FAM160B2	36%
chr8p21.3	21939833	21939882	DMTN	36%
chr8p21.3	22398199	22398248	PPP3CC	36%
chr8p21.3	20078945	20078994	ATP6V1B2	35%
chr8p23.1	11185430	11185479	MTMR9	35%
chr8p23.1	8890610	8890659	ERI1	34%
chr8p21.2	27316655	27316704	PTK2B	34%
chr8p12	30436174	30436223	GTF2E2	33%
chr8p21.1	28048518	28048567	ELP3	33%
chr8p21.1	28625279	28625328	INTS9	33%
chr8p11.22	38846564	38846613	TM2D2	25%
chr8p11.21	41788540	41788589	KAT6A	25%
chr8p11.23	38020937	38020986	LSM1	25%
chr8p11.23	37701676	37701725	BRF2	25%
chr13q14.2	49063443	49063492	RCBTB2	21%
chr13q14.13	45858012	45858061	GTF2F2	20%
chr13q14.2	50065906	50065955	SETDB2	20%
chr13q14.2	50591839	50591888	TRIM13	20%
chr13q14.2	50102892	50102941	PHF11	20%
chr13q14.3	53262319	53262368	SUGT1	19%
chr13q14.11	42897015	42897064	AKAP11	18%
chr16q24.1	85840509	85840558	COX4I1	16%
chr16q24.2	87741499	87741548	KLHDC4	16%
chr6q15	88228353	88228402	RARS2	15%
chr16q24.3	89977633	89977682	TCF25	15%
chr16q24.3	88927406	88927455	TRAPPC2L	15%
chr16q24.3	90034280	90034329	DEF8	15%
chr16q23.3	83846402	83846451	HSBP1	15%
chr6q15	89320073	89320122	RNGTT	14%
chr6q15	91225641	91225690	MAP3K7	13%
chr8q21.3	90802376	90802425	RIPK2	13%
chr8q24.13	124232447	124232496	C8orf76	13%
chr8q24.13	126379143	126379192	NSMCE2	13%
chr6q21	110146366	110146415	FIG4	12%
chr8q11.21	48648011	48648060	SPIDR	12%
chr8q21.13	82570430	82570479	IMPA1	12%
chr8q21.3	87573314	87573363	CPNE3	12%
chr8q22.2	101533288	101533337	ANKRD46	12%
chr8q23.2	110576909	110576958	EBAG9	12%
chr8q21.13	80947140	80947189	TPD52	12%
chr8q21.13	82671480	82671529	CHMP4C	12%
chr8q22.1	97346438	97346487	PTDSS1	12%
chr8q24.3	146199160	146199209	ZNF252P	11%

chr16q23.1	74907697	74907746	WDR59	11%
chr17p13.1	7529756	7529805	SAT2	9%
chr17p13.1	7363017	7363066	ZBTB4	9%
chr17p13.1	7491180	7491229	MPDU1	9%
chr17p13.2	4862717	4862766	SPAG7	9%
chr17p13.2	4840686	4840735	SLC25A11	9%
chr10q23.1	85912689	85912738	GHITM	8%
chr18q23	77806076	77806125	RBFA	8%
chr18q23	77662567	77662616	PQLC1	8%
chr6q22.31	125597068	125597117	HDDC2	7%
chr12p13.31	6840857	6840906	COPS7A	7%
chr16q22.1	70286329	70286378	AARS	7%
chr16q22.1	70514063	70514112	FUK	7%
chr17p13.2	4060604	4060653	CYB5D2	7%
chr17p13.2	3564717	3564766	CTNS	7%
chr17p13.2	4067363	4067412	ANKFY1	7%
chr18q21.1	43846636	43846685	C18orf25	7%
chr17p12	12895998	12896047	ELAC2	7%
chr18q21.1	47808738	47808787	CXXC1	6%
chr18q21.33	59711985	59712034	PIGN	6%
chr7q32.1	127231472	127231521	FSCN3	6%
chr7q32.1	127231472	127231521	ARF5	6%
chr18q21.1	44633811	44633860	HDHD2	6%
chr17p12	14111849	14111898	COX10	6%
chr10q24.32	104183242	104183291	CUEDC2	5%
chr10q24.1	99218137	99218186	MMS19	5%
chr18p11.22	9537980	9538029	RALBP1	5%
chr18p11.21	12329202	12329251	AFG3L2	5%
chr2q14.3	128015187	128015236	ERCC3	4%
chr2q14.3	128461058	128461107	SFT2D3	4%
chr2q24.1	158593153	158593202	ACVR1	4%
chr3q27.1	184052784	184052833	EIF4G1	4%
chr3q27.1	183530057	183530106	YEATS2	4%
chr13q34	115038036	115038085	CDC16	3%
chr3q26.2	169557364	169557413	LRRC31	3%
chr1p21.2	101007343	101007392	GPR88	2%
chr3q22.1	131083043	131083092	NUDT16P1	2%
chr9q33.3	129647594	129647643	ZBTB34	2%
chr9q33.3	128321925	128321974	MAPKAP1	2%
chr3q22.1	129023910	129023959	HMCES	2%
chr3q22.1	128631728	128631777	ACAD9	2%
chr12q13.3	57924185	57924234	DCTN2	2%
chr20q13.33	61962106	61962155	COL20A1	1%
chr2p21	47747953	47748002	KCNK12	1%
chr9q22.33	101611248	101611297	GALNT12	1%
chr9q31.1	102648734	102648783	RP11-60I3.5	1%
chr9p13.2	36677336	36677385	MELK	1%
chr9q31.1	104087030	104087079	LPPR1	1%
chr12q12	46316836	46316885	SCAF11	1%
chr20q11.21	30422380	30422429	MYLK2	1%

chr17q23.2	62016057	62016106	SCN4A	1%
chr12q12	46119857	46119906	LINC00938	1%

^ mapped to hg19

* Only OncoSNP (Yau et al., 2010) calls rank1+2 considered - see Methods.

Supplementary Table 7. Measures of cluster accuracy, compared to integrative clustering

	iCluster vs Gx alone (T variability)	iCluster vs Gx (fold change)	iCluster vs CN alone	Gx (T variability) vs Gx (fold change)
Adjusted Rand Index (Hubert 1985)	0.13	0.08	0.35	0.47
Variation of Information Index (Meilă 2007)	2.4	2.7	1.7	1.5

Gx = gene expression
CN = copy number
iCluster = integrative clustering, performed in iCluster+ (Mo et al., 2013).
T variability - tumour variability (see Gorlov et al. (2012) and Gorlov et al. (2014))

Supplementary Table 8. Summary of clinical characteristics of 2nd validation cohort (from Taylor et al., 2010).

	Primary tumour		Metastases	
	n = 181	%	n = 37	%
**Age (years)**				
Mean	60.9		60	
Range	37.3 - 83		41-82	
**Pre-operative PSA (ng/ml)**				
< 4	31	25%	4	13%
4 - 10	105	84%	6	19%
> 10	44	35%	22	69%
**Biopsy Gleason Grade**				
5	2	1%	-	-
6	101	56%	2	6%
7	61	34%	16	46%
8	11	6%	8	23%
9	6	3%	9	25%
**Clinical stage**				
cT1c	95	52%	8	22%
cT2c	76	42%	12	33%
cT3	9	5%	3	8%
pT4	-	-	1	3%
not available	-	-	9	25%
**Ethnicity**				
Black	29	16%	2	6%
Asian	4	2%	-	-
White Hispanic	-	0%	2	6%
White non-Hispanic	142	79%	32	89%
unknown	6	3%	-	-

*Only samples with aCGH and mRNA were used in this analysis: 109 primary tumours and 19 metastases.

Genes.SYMBOL	Coef.clust1 - Benign	t.clust1 - Benign	p.value.adj.clust1 - Benign	Genes.IlluminaID
AMACR	2.598	11.51	0	ILMN_1759670
HPN	2.191	14.38	0	ILMN_2358760
HPN	2.175	14.13	0	ILMN_1687235
PCA3	2.142	9.97	0	ILMN_3239648
AGR2	2.053	8.15	0	ILMN_3307841
PLA2G7	1.942	9.23	0	ILMN_1701195
ABCC4	1.931	9.58	0	ILMN_2194009
DLX1	1.832	12.24	0	ILMN_1672094
TRPM4	1.781	10.06	0	ILMN_1679401
SPON2	1.766	6.74	0	ILMN_1676099
ABCC4	1.689	8.93	0	ILMN_1788457
GDF15	1.591	5.29	4.00E-06	ILMN_2188862
TSPAN13	1.554	11.12	0	ILMN_1669881
THBS4	1.527	7.17	0	ILMN_1736078
RAP1GAP	1.5	11.04	0	ILMN_1776519
LEPREL1	-1.507	-5.76	0	ILMN_1657373
SMTN	-1.524	-8.75	0	ILMN_1785618
TCEAL2	-1.529	-10.94	0	ILMN_1765310
BNIPL	-1.53	-8.72	0	ILMN_1699989
PARM1	-1.532	-11.61	0	ILMN_1656560
ZNF827	-1.548	-9.72	0	ILMN_1727574
SCGB3A1	-1.55	-7.79	0	ILMN_1679666
PDK4	-1.566	-8.08	0	ILMN_1684982
SLC2A5	-1.611	-8.46	0	ILMN_1671337
PCP4	-1.615	-8.76	0	ILMN_1682326
FOS	-1.619	-5.05	1.00E-05	ILMN_1669523
KIAA1210	-1.628	-8.33	0	ILMN_1732962
COL17A1	-1.641	-10.68	0	ILMN_1651282
GCNT2	-1.67	-9.22	0	ILMN_1680390
FLRT3	-1.686	-10.56	0	ILMN_1805665
KIAA0353	-1.697	-10.38	0	ILMN_1712075
ANPEP	-1.736	-4.98	1.30E-05	ILMN_1763837
DES	-1.741	-9.68	0	ILMN_1698995
WFDC2	-1.817	-10.51	0	ILMN_3307693
OLFM4	-1.875	-3.96	0.000619	ILMN_2116877
MSMB	-1.884	-5.87	0	ILMN_2376313
MME	-1.904	-7.65	0	ILMN_1678170
MSMB	-1.917	-6.13	0	ILMN_1790906
PRDM8	-2.037	-9.68	0	ILMN_1802082
KRT15	-2.079	-10.15	0	ILMN_1770612
TP63	-2.259	-11.43	0	ILMN_3305055

Genes.SYMBOL	Coef.clust2 - Benign	t.clust2 - Benign	p.value.adj.clust2 - Benign	Genes.IlluminaID
HPN	1.637	10.98	0	ILMN_1687235
PCA3	1.567	7.53	0	ILMN_3239648
HPN	1.556	10.54	0	ILMN_2358760

Genes.SYMBOL	Coef.clust3 - Benign	t.clust3 - Benign	p.value.adj.clust3 - Benign	Genes.IlluminaID
KLK12	3.189	9.89	0	ILMN_1725912
AMACR	2.879	8.63	0	ILMN_1759670
SPON2	2.642	6.82	0	ILMN_1676099
FGFRL1	2.267	13.52	0	ILMN_1795865
TRPM4	2.221	8.49	0	ILMN_1679401
HPN	2.115	9.3	0	ILMN_1687235
CYP2J2	2.113	7.26	0	ILMN_1758731
GDF15	2.11	4.75	6.40E-05	ILMN_2188862
UNC5A	2.107	7.84	0	ILMN_1712913
HPN	2.033	9.03	0	ILMN_2358760
DLX1	2.016	9.11	0	ILMN_1672094
MYL2	2.009	3.92	0.001179	ILMN_2113807
PCA3	1.918	6.04	0	ILMN_3239648
MS4A8	1.913	7.28	0	ILMN_1808249
TRIB1	1.885	6.49	0	ILMN_1803811
MYL1	1.879	4.25	0.000392	ILMN_1700860
MYH7	1.833	3.91	0.001212	ILMN_1669714
KLK12	1.816	8.93	0	ILMN_2411511
TMSB15A	1.794	5.31	7.00E-06	ILMN_1681737
TFF3	1.718	2.97	0.018336	ILMN_1811387
TSPAN13	1.717	8.31	0	ILMN_1669881
CLDN8	1.717	6.21	0	ILMN_2072568
C15orf48	1.684	3.19	0.010379	ILMN_1805410
MB	1.664	4.96	2.90E-05	ILMN_1666109
HOXC4	1.657	7.89	0	ILMN_1719975
RAB17	1.65	8.56	0	ILMN_2052373
ELF5	1.614	8.42	0	ILMN_1813270
ABCC4	1.59	5.69	1.00E-06	ILMN_1788457
ABCC4	1.569	5.27	8.00E-06	ILMN_2194009
FLJ22184	1.554	8	0	ILMN_2387471
TSPAN13	1.534	8.25	0	ILMN_2130525
GNMT	1.53	4.03	0.000811	ILMN_1736238
CLDN8	1.525	6.53	0	ILMN_1746676
SLN	1.503	3.67	0.002579	ILMN_1778595
JAM3	-1.51	-7.46	0	ILMN_1769575
EDNRA	-1.513	-7.99	0	ILMN_1796629
LIMS2	-1.515	-8.06	0	ILMN_1760493
KRT13	-1.516	-3.11	0.012789	ILMN_1721218
ITGA5	-1.522	-5.53	3.00E-06	ILMN_1792679
MSRB3	-1.525	-8.08	0	ILMN_1676088
DPYSL3	-1.528	-8.35	0	ILMN_1679262
RARRES1	-1.528	-4.76	6.00E-05	ILMN_1800091
TGM4	-1.531	-2.66	0.038728	ILMN_1669363
CALD1	-1.531	-8.51	0	ILMN_1730487
HSPB8	-1.532	-6.91	0	ILMN_1791280
SLC14A1	-1.54	-8.21	0	ILMN_1805561
ALDH1A2	-1.541	-8.03	0	ILMN_1748538
PTGS2	-1.55	-4.58	0.000119	ILMN_2054297
HSPB6	-1.553	-5.85	1.00E-06	ILMN_1721283
TNFRSF19	-1.556	-4.92	3.40E-05	ILMN_1704154

ID1	-1.559	-5.24	9.00E-06	ILMN_1664861
CFB	-1.56	-3.26	0.00843	ILMN_1774287
SCGB3A1	-1.565	-5.32	7.00E-06	ILMN_1679666
LAMB3	-1.57	-8.11	0	ILMN_1715684
CCND2	-1.577	-7.02	0	ILMN_2067656
DLK2	-1.577	-7.88	0	ILMN_1676728
CPED1	-1.577	-8.66	0	ILMN_1677038
ANXA1	-1.58	-6.84	0	ILMN_2184184
C2orf40	-1.587	-5.28	8.00E-06	ILMN_1676822
PAGE4	-1.588	-4.88	3.90E-05	ILMN_1782754
AHNAK2	-1.589	-7.75	0	ILMN_3243156
ROR2	-1.594	-10.63	0	ILMN_1694426
ATP2B4	-1.595	-9.19	0	ILMN_1680579
PDE8B	-1.602	-6.17	0	ILMN_2301722
TGFB1I1	-1.625	-7.83	0	ILMN_2389876
MYH11	-1.641	-10.54	0	ILMN_1660086
LGALS1	-1.671	-9.33	0	ILMN_1723978
STOM	-1.676	-9.43	0	ILMN_1766657
TPM1	-1.679	-7.33	0	ILMN_2360710
CLIP3	-1.683	-7.12	0	ILMN_1789733
TP63	-1.683	-9.34	0	ILMN_2138801
ISL1	-1.687	-8.34	0	ILMN_1763390
BNIPL	-1.689	-6.51	0	ILMN_1699989
TPM1	-1.694	-7.37	0	ILMN_1716687
GPR124	-1.696	-8.19	0	ILMN_1773059
CCK	-1.707	-5.62	2.00E-06	ILMN_1712943
KRT5	-1.707	-7.45	0	ILMN_1801632
FLNA	-1.709	-6.57	0	ILMN_1687335
AHNAK2	-1.714	-7.72	0	ILMN_1657111
C12orf75	-1.735	-8.36	0	ILMN_1716382
PTRF	-1.746	-9.01	0	ILMN_1757552
TMEM158	-1.759	-6.33	0	ILMN_1792455
FLNC	-1.769	-6.11	0	ILMN_1715748
CFD	-1.771	-5.19	1.10E-05	ILMN_1777190
SORBS1	-1.78	-7.52	0	ILMN_1749792
ATP2B4	-1.799	-9.21	0	ILMN_2367753
SLC2A5	-1.801	-6.4	0	ILMN_1671337
GPX3	-1.809	-8.07	0	ILMN_1726666
FBLN1	-1.814	-6.14	0	ILMN_1700541
VSIG2	-1.82	-4.94	3.10E-05	ILMN_1666536
KCNAB1	-1.824	-6.4	0	ILMN_1744968
LDHB	-1.827	-8.48	0	ILMN_1728132
MEIS2	-1.836	-9.99	0	ILMN_1695945
COL17A1	-1.84	-8.11	0	ILMN_1651282
ADIRF	-1.842	-6.55	0	ILMN_1680110
PDK4	-1.864	-6.51	0	ILMN_1684982
CES1	-1.9	-6.8	0	ILMN_2359945
AOC1	-1.907	-4.57	0.000123	ILMN_1731433
TCEAL2	-1.938	-9.38	0	ILMN_1765310
KCNMB1	-1.944	-8.8	0	ILMN_1652065

CALD1	-2.014	-6.65	0	ILMN_1717990
CSRP1	-2.06	-10.58	0	ILMN_1811921
CAV1	-2.061	-10.35	0	ILMN_2149226
MYL9	-2.111	-9.1	0	ILMN_1675062
MYLK	-2.125	-8.92	0	ILMN_2364768
PNCK	-2.129	-9.57	0	ILMN_1697189
TAGLN	-2.13	-9.2	0	ILMN_2400935
FLRT3	-2.184	-9.26	0	ILMN_1805665
DES	-2.208	-8.31	0	ILMN_1698995
SMTN	-2.212	-8.59	0	ILMN_1785618
CNN1	-2.216	-9.2	0	ILMN_1810054
KIAA1210	-2.232	-7.73	0	ILMN_1732962
PGM5	-2.297	-9.52	0	ILMN_1709590
PGM5	-2.311	-9.7	0	ILMN_1781388
PGM5	-2.396	-9.33	0	ILMN_2271149
PRDM8	-2.403	-7.73	0	ILMN_1802082
KRT15	-2.48	-8.19	0	ILMN_1770612
TP63	-2.554	-8.75	0	ILMN_3305055
WFDC2	-2.555	-10	0	ILMN_3307693
OLFM4	-2.566	-3.67	0.002634	ILMN_2116877
MSMB	-2.578	-6.71	0	ILMN_1699243
MYLK	-2.634	-9.52	0	ILMN_1691476
PCP4	-2.649	-9.73	0	ILMN_1682326
SYNM	-2.65	-10.97	0	ILMN_1712075
MSMB	-3.216	-6.78	0	ILMN_2376313
MSMB	-3.226	-6.98	0	ILMN_1790906

Genes.SYMBOL	Coef.clust4 - Benign	t.clust4 - Benign	p.value.adj.clust4 - Benign	Genes.IlluminaID
TRPM4	2.136	11.2	0	ILMN_1679401
TFF3	1.892	4.49	0.000126	ILMN_1811387
HPN	1.765	10.65	0	ILMN_1687235
FLJ22184	1.712	12.09	0	ILMN_2387471
HPN	1.688	10.29	0	ILMN_2358760
C1orf64	1.63	5.02	1.60E-05	ILMN_2066088
GDF15	1.506	4.65	6.90E-05	ILMN_2188862
PRDM8	-1.51	-6.66	0	ILMN_1802082
MSMB	-1.548	-5.53	2.00E-06	ILMN_1699243
MSMB	-1.674	-4.97	2.00E-05	ILMN_1790906
TP63	-1.698	-7.98	0	ILMN_3305055
OLFM4	-1.742	-3.42	0.004484	ILMN_2116877
MSMB	-1.748	-5.05	1.50E-05	ILMN_2376313

Genes.SYMBOL	Coef.clust5 - Benign	t.clust5 - Benign	p.value.adj.clust5 - Benign	Genes.IlluminaID
AMACR	2.596	12.68	0	ILMN_1759670
HPN	2.131	15.27	0	ILMN_1687235
HPN	2.106	15.25	0	ILMN_2358760
PCA3	2.081	10.68	0	ILMN_3239648
ABCC4	1.924	10.52	0	ILMN_2194009
TRPM4	1.907	11.88	0	ILMN_1679401
AGR2	1.889	8.27	0	ILMN_3307841
C1orf64	1.749	6.4	0	ILMN_2066088
THBS4	1.74	9	0	ILMN_1736078
GDF15	1.642	6.02	0	ILMN_2188862
COMP	1.596	5.66	1.00E-06	ILMN_1677636
FLJ22184	1.513	12.69	0	ILMN_2387471
RAB17	1.508	12.74	0	ILMN_2052373
COL9A2	1.507	8.05	0	ILMN_1685122
SLC2A5	-1.504	-8.71	0	ILMN_1671337
PDK4	-1.506	-8.57	0	ILMN_1684982
MSMB	-1.526	-5.38	3.00E-06	ILMN_1790906
DES	-1.555	-9.54	0	ILMN_1698995
PRDM8	-1.665	-8.73	0	ILMN_1802082
MME	-1.688	-7.48	0	ILMN_1678170
KRT15	-1.837	-9.88	0	ILMN_1770612
TP63	-1.849	-10.31	0	ILMN_3305055

Supplementary Table 10. Refined gene signature for each patient subtype

iCluster1	iCluster2	iCluster3	iCluster4	iCluster5
ATP6V1B2	AKAP11	AKAP11	ACVR1	AFG3L2
BRF2	ANKRD46	ANKRD46	ANKRD46	ANKRD46
C8orf58	ATP6V1B2	ARF5	C8orf76	ATP6V1B2
CCAR2	C18orf25	ATP6V1B2	CCAR2	BRF2
CHMP7	C8orf58	C8orf58	CHMP4C	C18orf25
COPS7A	CCAR2	C8orf76	CHMP7	C8orf58
CTNS	CDC16	CCAR2	COX10	CCAR2
CXXC1	CHMP4C	CHMP4C	COX4I1	CHMP7
DMTN	CHMP7	COL20A1	CPNE3	CUEDC2
ELP3	CUEDC2	COX4I1	CTNS	CXXC1
ERI1	DCTN2	CPNE3	CXXC1	DMTN
FAM160	DMTN	DMTN	CYB5D2	EBAG9
FAM160B2	ELP3	EBAG9	DEF8	ELAC2
GHITM	ERI1	ELAC2	DMTN	ELP3
GTF2E2	FAM160B2	ELP3	EBAG9	ERI1
INTS9	GHITM	ERI1	ELAC2	FAM160B2
KAT6A	GTF2E2	FSCN3	ELP3	FIG4
KLHDC4	GTF2F2	GTF2E2	ERCC3	GPR88
LSM1	HDDC2	HSBP1	FAM160B2	GTF2E2
MAPKAP1	HDHD2	IMPA1	FUK	HDHD2
MTMR9	HSBP1	INTS9	GHITM	INTS9
NUDT16P1	INTS9	MAPKAP1	GTF2F2	KAT6A
PPP3CC	KCNK12	NSMCE2	IMPA1	LINC00938
PQLC1	LSM1	PPP3CC	INTS9	LSM1
PTK2B	MAP3K7	PTDSS1	KAT6A	MAP3K7
R3HCC1	MAPKAP1	PTK2B	KLHDC4	MAPKAP1
SAT2	MTMR9	R3HCC1	MAPKAP1	MELK
SCAF11	PHF11	RIPK2	MELK	MMS19
SORBS3	PIGN	RP11-60I3.5	MMS19	MTMR9
TM2D2	PPP3CC	SORBS3	MPDU1	PPP3CC
TPD52	PTDSS1	SPIDR	MTMR9	PTDSS1
ZBTB4	PTK2B	TCF25	NSMCE2	PTK2B
	R3HCC1	TPD52	NUDT16P1	R3HCC1
	RARS2	ZBTB34	PIGN	RALBP1
	RCBTB2	ZBTB4	PQLC1	RARS2
	RNGTT	ZNF252P	PTK2B	RNGTT
	SETDB2		R3HCC1	SCAF11
	SORBS3		RBFA	SCN4A
	SPAG7		RCBTB2	SETDB2
	SUGT1		RIPK2	SLC25A11
	TM2D2		SAT2	SORBS3
	TRIM13		SCAF11	TM2D2
	WDR59		SFT2D3	TPD52
	ZBTB4		SLC25A11	ZBTB34
			SORBS3	
			TCF25	
			TRAPPC2L	
			TRIM13	
			YEATS2	
			ZBTB4	

Supplementary Table 11. Published molecular signatures of primary prostate cancer

Study	Signature type	# genes	genes/pathways identified
Cuzick et al. (2011)	RNA	31	cell cycle proliferation
Irshad et al. (2013)	RNA	19	B2M, CAT, CDKN1A, CFH, CLIC4, CLU, CTSH, CXCL1, FGFR1, GPX3, IGF1, ITM2A, LGALS3, MECP2, MSN, NFE2L2, PMP22, SERPING1, TXNIP
Sharma et al. (2013)	DNA	16	PECI, TNFSF10, ABHD2, XRCC3, MAD1L1, SEC61A1, GFM1, TSPAN13, STIL, EIF2B5, TM42F1, NDUFB11, SLC26A2, AGR2, NT5DC3, TRMT12
Ramos-Montoya et al. (2014)	RNA	222	E2F1-mediated cell cycle
Lalonde et al. (2014)	DNA	100	lipid metabolism genes enriched
Oncotype Dx prostate cancer assay	RNA	17	Genomic Prostate Score (GPS) given by expression levels of BGN, COL1A1, SFRP4, TX2, AZGP1, FAM13C, KLK2, SRD5A2, FLNC, GSN, GSTM2, TPM2, ARF1, ATP5E, CLTC, GPS1, PGK1

Supplementary Table 12. Comparison of prognostic power between our 100-gene signature and published PC signatures, based on p-value.

	Performance in Stockholm (all features)	Performance in Taylor et al. (2010) (gene expression)
Cambridge-derived novel 100-gene signature	**8.96E-06**	**0.0213**
Sharma et al. (2013)	0.1086	0.0076
Cuzick et al. (2011)	0.7471	0.4076
Irshad et al. (2014)	0.7146	0.7727
HES6/Ramos-Montoya et al. (2014)	0.0260	0.1384
Lalonde et al (2014)*	0.0891	0.7691
OncotypeDX	0.7766	0.3798

* Only copy number data available
